# IAROA: An Enhanced Attraction–Repulsion Optimisation Algorithm Fusing Multiple Strategies for Mechanical Optimisation Design

**DOI:** 10.3390/biomimetics10090628

**Published:** 2025-09-17

**Authors:** Na Zhang, Ziwei Jiang, Gang Hu, Abdelazim G. Hussien

**Affiliations:** 1 Art College, Xi’an University of Science and Technology, Xi’an 710054, China; 2 Department of Applied Mathematics, Xi’an University of Technology, Xi’an 710054, China; jiangziwei2021@sian.com (Z.J.); hugang@xaut.edu.cn (G.H.); 3 Department of Computer and Information Science, Linköping University, 581 83 Linköping, Sweden; abdelazim.hussien@liu.se

**Keywords:** attraction–repulsion optimisation algorithm, elite dynamic opposite learning strategy, dimension learning-based hunting search strategy, Cauchy distribution inverse cumulative perturbation strategy, engineering optimisation problems

## Abstract

Attraction–Repulsion Optimisation Algorithm (AROA) is a newly proposed metaheuristic algorithm for solving global optimisation problems, which simulates the equilibrium relating to the attraction and repulsion phenomenon that occurs in the natural world, and aims to achieve a good balance between the development exploration phases. Although AROA has a more significant performance compared to other classical algorithms on complex realistic constrained issues, it still has drawbacks in terms of diversity of solutions, convergence precision, and susceptibility to local stagnation. To further improve the global optimisation search and application ability of the AROA algorithm, this work puts forward an Improved Attraction–Repulsion Optimisation Algorithm based on multiple strategies, denoted as IAROA. Firstly, the elite dynamic opposite (EDO) learning strategy is used in the initialisation phase to enrich the information of the initial solution and obtain high-quality candidate solutions. Secondly, the dimension learning-based hunting (DLH) exploration tactics is imported to increase the candidate solution diversity and enhance the trade-off between local and global exploration. Next, the pheromone adjustment strategy (PAS) is used for some of the solutions according to the threshold value, which extends the search range of the algorithm and also accelerates the convergence process of the algorithm. Finally, the introduction of the Cauchy distribution inverse cumulative perturbation strategy (CDICP) improves the local search ability of the algorithm, avoids falling into the local optimum, and improves the convergence and accuracy of the algorithm. To validate the performance of IAROA, algorithms are solved by optimisation with the original AROA and 13 classical highly cited algorithms on the CEC2017 test functions, among six engineering design problems of varying complexity. The experimental results indicate that the proposed IAROA algorithm is superior in terms of optimisation precision, solution stability, convergence, and applicability and effectiveness on different problems, and is highly competitive in solving complex engineering design problems with constraints.

## 1. Introduction

Metaheuristic optimisation algorithms have attracted much attention in recent years as an efficient method for solving complex optimisation problems. It stems from the exploration of the inadequacies of traditional optimisation methods and the need for more flexible and efficient solution strategies. Traditional optimisation methods, which require precise mathematical modelling and depend on the specific structure of the problem, become difficult to apply as the size and complexity of the problem increase. Metaheuristic optimisation algorithms provide new solution ideas and have evolved from simple heuristics to hybrid metaheuristic algorithms combined with advanced techniques. The basic principle of metaheuristic algorithms is to adaptively adjust the search strategy to the problem characteristics during the search process. They do not focus on specific details, but rather on finding an approximate optimal solution by effectively exploring the search space, which is highly flexible and can be widely applied to various optimisation problems.

Heuristic algorithms are commonly categorised into the following four types: algorithms based on evolution, algorithms based on physical principles, algorithms based on group intelligence, and algorithms that simulate human behaviour. Algorithms based on evolution are a type of high-powered optimisation algorithm that mimic evolutionary procedures such as natural selection and genetic variation. Such algorithms include Genetic Algorithms (GA) [1], Differential Evolutionary Arithmetic (DE) [2], Biogeography Based Optimisation (BBO) [3], Immunity Algorithms (IA) [4], and so on. Algorithms based on physical principles are a category of innovative optimisation algorithms that draw on the laws of operation of physical phenomena in nature or are directly motivated by physical principles, and simulate these physical processes to find the optimum or approximate resolution of problems. This class of algorithms includes Gravitational Search Algorithms (GSA) [5], Simulated Annealing Algorithms (SAA) [6], Kepler Optimisation Algorithms (KOA) [7], Light Spectrum Optimizer (LSO) [8], Multi-Verse Optimisers (MVO) [9], Polar Lights Optimisation (PLO) [10], etc. Algorithms based on group intelligence are a category of state-of-the-art optimisation algorithms inspired by group behaviour and group intelligence in nature and society, which find the optimal or approximate solution to a problem by mimicking the mechanisms of interaction, co-operation, and competition between individuals in a group. Such algorithms include Harris Hawk Optimisation (HHO) [11], Mountain Gazelle Optimizer (MGO) [12], Nutcracker Optimization Algorithm (NOA) [13], Honey Badge Algorithm (HBA) [14], Firefly Algorithm (FA) [15], Remora Optimization Algorithm (ROA) [16], Sled Dog Optimizer (SDO) [17], and so on. Human behaviour-based algorithms are optimisation tactics that are deeply inspired by human intelligence and behavioural patterns. They simulate the decision-making process, learning strategies, and problem-solving techniques adopted by humans when facing complicated problems, thus effectively increasing the efficiency and performance of problem-solving. Such algorithms include Preschool Education Optimisation Algorithm (PEOA) [18], Teaching-Learning-Based Optimization Algorithm (TLBO) [19], Hiking Optimisation Algorithm (HOA) [20], Enterprise Development (ED) [21], Football Team Training Algorithm (FTTA) [22].

Inspired by physical phenomena or the existence of some kind of attraction between groups of animals, Karol Cymerys et al. [23] proposed the Attraction–Repulsion Optimisation Algorithm (AROA) and experimentally compared it with high-performance algorithms on the CEC 2014, 2017, and 2020 evaluated functions. The experimental outcomes show that it is highly competitive as well as highlighting the applicability of AROA for solving complex real-world problems.

To further improve the performance of the AROA algorithm, this paper proposes to combine the four strategies and suggests an improved attraction–rejection optimisation algorithm (IAROA). The main contributions of this paper are as follows:

(1) Based on the defects in the primordial AROA algorithm, an improved attraction–rejection optimisation algorithm called IAROA is raised by importing the EDO strategy, DLH strategy, the PAS strategy, and the CDICP strategy.
(2) Some of the typical, recently raised, highly cited, CEC ranking, and refined classical intelligent algorithms are elected as the contrasting algorithms. The experiments are carried out in the 30D, 50D, and 100D CEC2017 test environments, respectively. The outcomes demonstrate that the IAROA algorithm exhibits remarkable superiority in most of the functions in the CEC2017 test set.
(3) IAROA is applied in six engineering design issues of varying complexity, including Alkylation Unit, Industrial refrigeration System, Speed Reducer, Robot gripper problem, and Himmelblau’s Function, and 12 highly cited optimisation algorithms were also picked for contrast.

The remainder of this article is structured as follows: Section 2 first gives a brief overview of the standard AROA, followed by an improved AROA algorithm, detailing the four improvement strategies incorporated, and briefly analysing the algorithmic complexity of IAROA, offering pseudo-code as well as a design flowchart for IAROA. Section 3 briefly describes the comparative algorithms for the experiments and provides the outcome of analysing the evaluation indicators in the CEC2017 experimental environment so as to verify the superiority of IAROA’s performance. Section 4 applies IAROA to six engineering design problems of varying complexity and selects 12 highly cited optimisation algorithms for comparison. At the end, Section 4 will draw the outcomes of the research and present future research orientations and work programmes.

## 2. Theory

### 2.1. Overview of the Attraction–Repulsion Algorithm

AROA is a new intelligent optimisation algorithm proposed by Karol Cymerys et al. [23] in 2024. This algorithm is inspired by a special attraction phenomenon that exists in human or natural animal populations, and the attraction–rejection operator is proposed accordingly. At the same time, the algorithm comprehensively takes into account a variety of factors affecting the position of candidate solutions in the search space, like the attraction of the optimal solution, the memory mechanism, the influence of random solutions from the general population, and the local search operator.

#### 2.1.1. Initialisation

In AROA, the initial positions of all solutions are determined stochastically,

(1) $$x_{i}=rand⊗(x_{\max}-x_{\min})+x_{\min}$$

where ***x***_min_, ***x***_max_ is the search space boundary, and *rand* is a stochastic number with values evenly distributed in [0,1].

#### 2.1.2. Attraction and Repulsion

**Figure 1:**
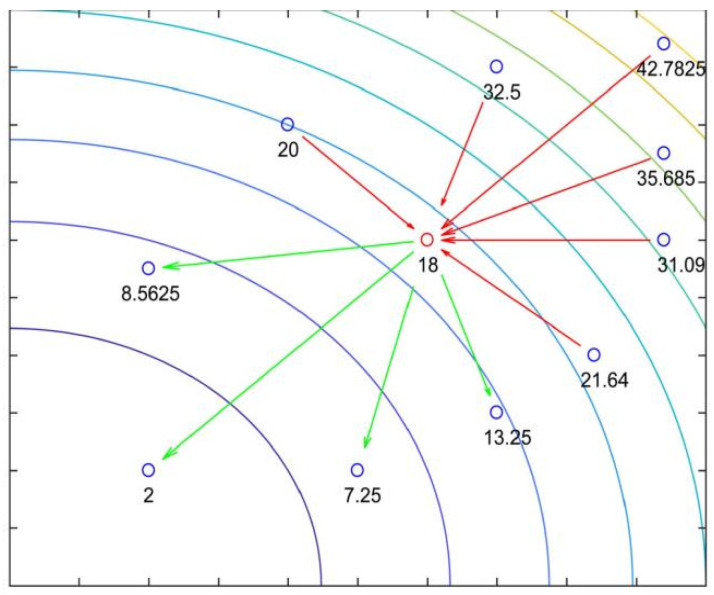
Attraction–repulsion schematic.

In the optimisation procedure, the location of a candidate solution is renewed by information about the fitness of the remaining population realisations. Specifically, the neighbouring solutions exert an attraction or repulsion effect on the candidate solutions, and the extent of this effect depends on the distance from other solutions. The attraction–repulsion concept for the search space of a given region is shown in Figure 1.

Firstly, define a matrix of distances *D*,

(2) $$D=\left[\begin{array}{cccc} d_{1,1} & d_{1,2} & \cdots & d_{1,n}\\ d_{2,1} & d_{2,2} & \cdots & d_{2,n}\\ \cdots & \cdots & \cdots & \cdots \\ d_{n,1} & d_{n,2} & \cdots & d_{n,n} \end{array}\right]$$

secondly, define the distance between the *i*th and the *j*th candidate solution in terms of the squared Euclidean distance,

(3) $$d^{2}\left(x_{i},x_{j}\right)={\sum_{k=1}^{\dim}\left(x_{i}^{k}-x_{j}^{k}\right)}^{2}$$

where *dim* denotes the dimensions, *x_i_* and *x_j_* are the *i*th and *j*th positions of solution severally. *d_i_*_,max_ is the distance from the *i*th solution to the furthest solution among all the candidate solutions. Considering the *i*th solution and the neighbouring *k* candidate solutions, the attraction–repulsion operator is defined as follows,

(4) $$n_{i}=\frac{1}{n}\sum_{j=1}^{k}c\cdot (x_{j}-x_{i})\cdot I(d_{i,j},d_{i,\max})\cdot s(f_{i},f_{j})$$

where *c* is the step size and *I* is the function used to measure the extent to which the *j*th solution has an impact on the other solutions,

(5) $$I(d_{i,j},d_{i,\max})=1-\frac{d_{i,j}}{d_{i,\max}}$$

the function ***s*** is presented in Equation (6),

(6) $$s(f_{i},f_{j})=\left\{\begin{array}{l} 1,f_{i}>f_{j}\\ 0,f_{i}=f_{j}\\ -1,f_{i}<f_{j} \end{array}\right.$$

The number of neighbours *k*, which should decrease with the increasing number of iterations, is thus shown in Equation (7):

(7) $$k=\left[\left(1-\frac{t}{t_{\max}}\right)\cdot n\right]+1$$

#### 2.1.3. Attracted by the Optimal Solution

In AROA, the vector representing the vector attracted to the optimal solution *b_i_* is computed as shown in Equation (8):

(8) $$b_{i}=\left\{\begin{array}{l} c\cdot m\cdot (x_{best}-x_{i}),r_{1}\geq p_{1}\\ c\cdot m\cdot (a_{1}⊗x_{best}-x_{i}),r_{1}<p_{1} \end{array}\right.$$

where *x_best_* is the optimum solution; *r*_1_ is a stochastic digit in [0,1]; *p*_1_ is a probability threshold; *a*_1_ is a vector of stochastic digits within [0,1].

Factor *m* is in charge of maintaining the trade-off between discovery and development, dynamically controlling the impact of the optimum solution. It is defined as shown in Equation (9):

(9) $$m=\frac{1}{2}\left(\frac{\exp(z)-1}{\exp(z)+1}+1\right)$$

where Equation (12) is the transformed hyperbolic tangent function, $z=18\cdot t/t_{\max}-4$.

#### 2.1.4. Local Search Operator

The AROA algorithm also randomly selects a local search operator for each candidate solution, and there are three of these operators. The first operator draws on the principle of Brownian motion, as shown in Equation (10):

(10) $$r_{B}=u_{1}⊗N(0,f_{r_{1}}\cdot (1-\frac{t}{t_{\max}})\cdot (x_{max}-x_{min}))$$

where *f*_*r*_1__ is a decimal number, *N* is a vector of digits randomly generated according to a normal distribution, and *u*_1_ is a two-valued vector.

The second local search operator combines a trigonometric function and a roulette wheel selection mechanism. This operator is shown in Equation (11):

(11) $$r_{tri}=\left\{\begin{array}{l} f_{r2}\cdot u_{2}\cdot (1-\frac{t}{t_{\max}})\cdot \sin\left(2r_{5}\pi \right)⊗\left|a_{2}⊗x_{w}-x_{i}\right|,r_{4}<0.5\\ f_{r2}\cdot u_{2}\cdot (1-\frac{t}{t_{\max}})\cdot \cos\left(2r_{5}\pi \right)⊗\left|a_{2}⊗x_{w}-x_{i}\right|,r_{4}\geq 0.5 \end{array}\right.$$

where *r*_4_,*r*_5_ are stochastic digits in [0,1], *a*_2_ is a vector of random numbers in [0,1], and the solution *x_w_* is represented by the roulette wheel selection method.

To ensure broad coverage of the search space and population diversity, the third local search operator introduces new candidate solutions by randomly selecting positions in the search space, and the related operator is given by Equation (12):

(12) $$r_{R}=u_{3}⊗(2\cdot a_{3}-o)⊗(x_{max}-x_{min})$$

where *u*_1_ is a binomial vector with threshold value tr2, *a*_3_ is a vector of arbitrary counts, and *o* is a unit matrix.

Therefore, the position update is defined, as shown in Equation (13):

(13) $$r_{i}=\left\{\begin{array}{l} \left\{\begin{array}{l} r_{B},r_{3}>0.5\cdot \frac{t}{t_{\max}}+0.25\\ r_{tri},r_{3}\leq 0.5\cdot \frac{t}{t_{\max}}+0.25 \end{array}\right.,r_{2}<p_{2}\\ r_{R},r_{2}\geq p_{2} \end{array}\right.$$

where both *r*_2_ and *r*_3_ are stochastic generated digits in [0,1].

Finally, the position of the candidate is defined as illustrated in Equation (14):

(14) $$x_{i}(t)=x_{i}(t-1)+n_{i}+b_{i}+r_{i}$$

where ***n_i_*** is the one determined by the attraction–repulsion operator, which captures the relationship among the candidate solution and the other candidate solutions, ***b_i_*** is the modelling of the appeal of the optimum candidate, *r*_i_ denotes motion of the local exploration operator.

#### 2.1.5. Population-Based Operations

Two population-based operators are used to interact with every solution. The first arithmetic produces irritations that imitate the forming of vortices, as well as two stochastic solutions to influence the candidate solutions. It is defined as shown in Equation (15):

(15) $$x_{i}=\left\{\begin{array}{l} x_{i}+c_{f}\cdot (u_{4}⊗(a_{4}(x_{\max}-x_{\min})+x_{\min})),r_{6}<e_{f}\\ x_{i}+(c_{f}\cdot (1-r_{7})+r_{7})(x_{r_{8}}-x_{r_{9}}),r_{6}\geq e_{f} \end{array}\right.$$

where *u*_4_ is a binary variable vector with the threshold 1 − *e_f_*, *a*_4_ is a vector of stochastically produced digits, *r*_7_ is a vector of stochastically produced digits in [0,1], *e_f_* takes the value of 0.2, *r*_8_ and *r*_9_ form the index of randomly selecting an individual as well as $c_{f}={\left(1-\frac{t}{t_{\max}}\right)}^{3}$.

The second key stimulus affecting the population is a memory mechanism that records the previous position and fitness of each candidate solution. When a candidate solution fails to improve during the evolutionary process, it “gets back on track” through the memory mechanism and continues to participate in the subsequent search and optimisation process. The definition is shown in Equation (16):

(16) $$x_{i}(t)=\left\{\begin{array}{l} x_{i}(t),f(x_{i}(t))<f(x_{i}(t-1))\\ x_{i}(t-1),f(x_{i}(t))\geq f(x_{i}(t-1)) \end{array}\right.$$

### 2.2. Improved Attraction–Repulsion Algorithm

Although the AROA algorithm has merits like excellent optimisation ability and quick convergence speed, it is inevitably vulnerable to becoming trapped in local optimum, resulting in low computational accuracy and affecting its optimisation ability. To optimise the performance of the AROA, in this section, the AROA is refined by integrating the below four tactics and proposes an improved Attraction–Repulsion Optimisation Algorithm (IAROA).

(1) EDO strategy to enable a more uniform spread of the primary population and to acquire a high-quality starting population.
(2) DLH strategy which scales up the trade-off among local exploitation and global search, while retaining the multiplicity of solutions.
(3) CDICP strategy, which increases the reliability of the algorithm search and the variety of candidate solutions, thus effectively preventing the algorithm from converging to the local optimum prematurely.
(4) PAS strategy to raise the overall running efficiency of the algorithm, both to expedite the convergence procedure and to ensure that the ultimately optimal solution is gained with a higher degree of precision.

#### 2.2.1. Elite Dynamic Opposite Learning Strategy (EDO)

The optimisation performance of the swarm intelligence optimisation algorithm is greatly affected by the quality of the initial solution, and a high-quality initial population can accelerate the convergence speed of the algorithm and help to find the global optimal solution [24]. The standard AROA algorithm tends to initialise the population by a random initialisation method, which can easily lead to poor population diversity and is not conducive to the fast convergence of the algorithm.

Inspired by quasi-opposite-based learning (QOBL) and quasi-reflection-based learning (QRBL), Xu et al. [25] (2020) proposed dynamic opposite learning (DO). The search space of DO exhibits asymmetric properties and can be dynamically adjusted based on random opposites. This dynamically changing property greatly enriches the diversity of the search space, which in turn effectively enhances the exploration capability of the algorithm. Based on this, this paper applies DO to the initialisation phase of AROA, and the mathematical model is shown in Equation (17),

(17) $$\begin{array}{l} X_{op}={\mathrm{LB}}_{op}+{\mathrm{UB}}_{op}-X,\\ X_{RO}=u\cdot X_{op},\\ X_{DO}=X+v\cdot \left(X_{RO}-X\right) \end{array}$$

where ***X_op_*** is the solution generated by reverse learning and *u*,*v* are astochastic digits evenly distributed at [0,1]. These newly produced inverse solutions ***X_op_*** are merged with the primitive solution set ***X*** and the elite portion of them is filtered out by a sorting operation. That is, the solutions are sequenced based on their fitness values, and the top-performing solutions are selected to form the original population. It can effectively improve the variety and quality within the initiative population. The solution generated by backward learning makes the initial population more uniform in distribution. Such an initial population is not only conducive to the fast convergence of this algorithm, but it can also strengthen the global exploration ability of it, thus further enhancing its overall performance.

#### 2.2.2. Dimension Learning-Based Hunting Search Strategy

Dimensional Learning Heuristic (DLH) [26], proposed by Nadimi-Shahrakiden et al. (2021), provides us with a novel and efficient optimisation idea. Under the framework of DLH, DLH constructs a neighbourhood for each candidate solution, which can promote information sharing among all candidate solutions and broaden the search scope. In addition, DLH introduces the influence of stochastic solutions on candidate solutions, balances local and global exploration phases, and enhances its flexibility to maintain the diversity of solution populations. In this section, the mathematical model is shown in Equation (18),

(18) $${x_{\_dlh}}_{i}^{d}\left(t+1\right)=x_{i}^{d}\left(t\right)+rand\cdot \left(x_{neighbor\left(d\right)}^{d}(t)-x_{r_{1}}^{d}\right)$$

where $x_{neighbor\left(d\right)}^{d}(t)$ is the random domain solution selected by the Euclidean distance among the candidate solutions, and a radius between *x_i_*(*t*) and the candidate solution, *r*_1_ is the index of one stochastic solution.

After obtaining the candidate populations generated by DLH, the final population in generation *t* + 1 is determined by greedy selection:

(19) $$x_{i}^{t+1}=\left\{\begin{array}{ll} {x_{\_dlh}}_{i}^{t} & \;f({x_{\_dlh}}_{i}^{t})\leq f(newx_{i}^{t})\\ newx_{i}^{t} & \;f({x_{\_dlh}}_{i}^{t})>f(newx_{i}^{t}) \end{array}\right.$$

where $newx_{i}^{t}$ denotes the location of the *i*th candidate solution in the *t*th generation of candidate species solutions generated by the attracting and repelling operators, *i* = 1, 2,⋯, *n*.

#### 2.2.3. Cauchy Distribution Inverse Cumulative Perturbation Strategy

**Figure 2:**
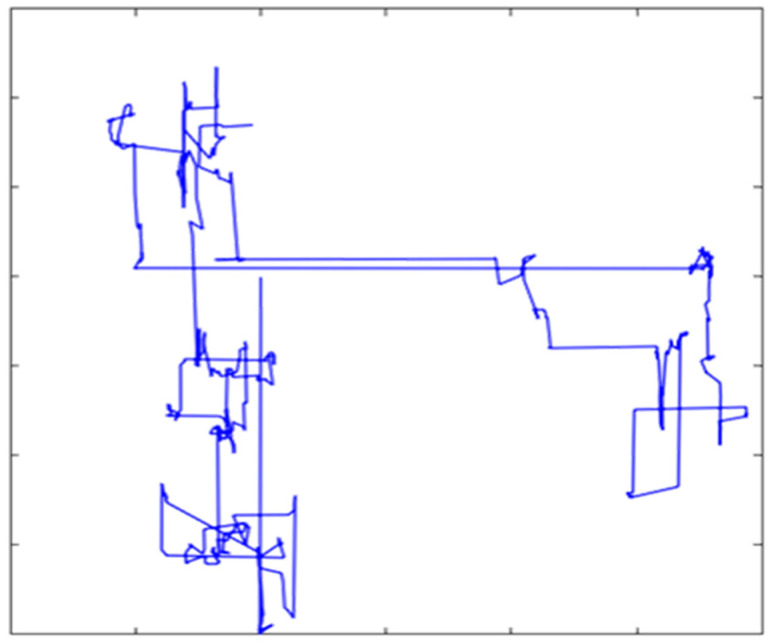
Step size of the Cauchy inverse cumulative distribution operator.

The Cauchy distribution inverse accumulation [27] is a new tangent function proposed by Wang et al. (2022). The step size generated by this function is generally more average, with occasional large step sizes. The flight trajectory diagram of the CDICP in a given search area is shown in Figure 2. When used as a scale to control the step size, it can effectively enhance the stability of the algorithm search. In most cases, the AROA algorithm has a limited scope of exploration. In this study, we take advantage of this property of the CDICP to perform tangent flight perturbation operations on all candidate solutions at each iteration, to expand the distribution range of the solutions in the search space and deepen the degree of exploration of the solution space. This can not only increase the diversity of solutions and accelerate the convergence speed of the algorithm, but also alleviate the problem that the AROA algorithm tends to fall into local optimality in the late iterations. The specific operation is shown in Equations (20) and (21) below:

(20) $$f^{-1}\left(p;a,b\right)=a+b\tan\left(\pi \left(p-\frac{1}{2}\right)\right)$$

(21) $$x_{i}\left(t+1\right)=\left\{\begin{array}{l} x_{i}\left(t\right)+\left(x_{best}-x_{i}\left(t\right)\right)f^{-1}\left(p;a,b\right),p<0.5\\ x_{i}\left(t\right)+\left(x_{mean}-x_{i}\left(t\right)\right)f^{-1}\left(p;a,b\right),p\geq 0.5 \end{array}\right.$$

where *p* is an astochastic digit in [0,1], and *d* is the dimension. The standard Cauchy distribution location parameter *a* takes the value 0, and a scale parameter *b* of 0.01. *x_best_* is the current optimal solution.

#### 2.2.4. Pheromone Adjustment Strategy (PAS)

The Black Widow Optimisation Algorithm (BWOA) was proposed by Hayyolalam V. et al. (2020) inspired by black widow spiders’ predation [28], which simulates the efficient search and strategy selection capabilities exhibited by black widow spiders during predation and aims to solve complex optimisation problems. Pheromone is another trait of black widow populations that plays an important role in the mating process. Its definition is shown in Equation (22):

(22) $$pheromone(i)=\frac{f_{\max}-f(i)}{f_{\max}-f_{\min}}$$

when this pheromone is too small, the individual will be replaced by a new individual, modelled as shown in Equation (23):

(23) $$x_{i}\left(t\right)=x^{*}\left(t\right)+\frac{1}{2}\left[x_{r_{1}}\left(t\right)-{\left(-1\right)}^{\sigma }\cdot x_{r_{2}}\left(t\right)\right]$$

where $x^{*}\left(t\right)$ is the optimum solution, *r*_1_,*r*_2_ are the indexes of two stochastic individuals, *σ* taken as 0 or 1.

This section introduces the PAS strategy in the population-based operation session. Specifically, when the pheromone level of a candidate solution is monitored to be lower than a preset threshold (which is set to be 0.3 in this section), a replacement operation is carried out for the candidate solution according to Equation (23). Through this dynamic adjustment of the pheromone level of the candidate solution, the algorithm is guided to explore the solution space more efficiently, which in turn accelerates the convergence speed of the algorithm and improves the accuracy of the optimal solution obtained in the end.

#### 2.2.5. Steps of the Improved Attraction–Repulsion Optimisation Algorithm

**Figure 3:**
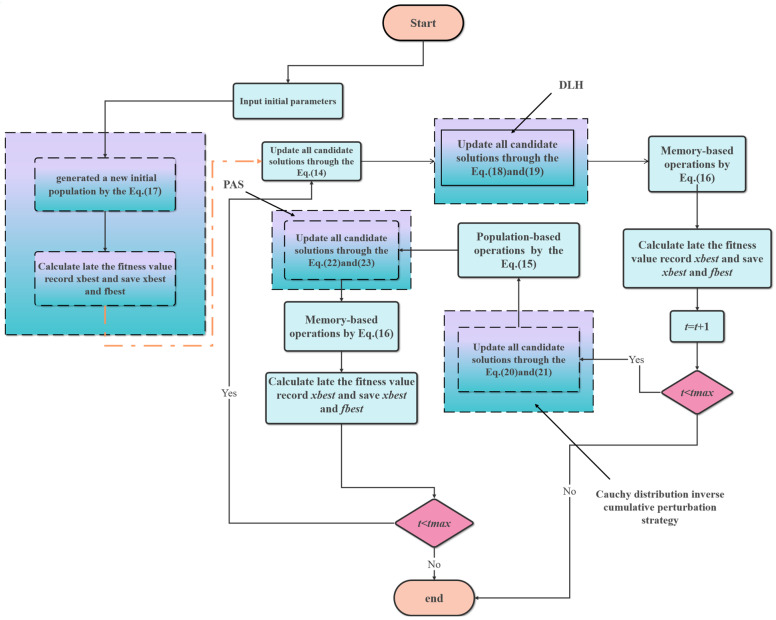
Flowchart of the IAROA algorithm.

In this paper, the improved AROA algorithm (IAROA) is proposed by adding several improvement strategies, namely, EDO strategy, DLH mechanism, CDICP strategy, and PAS strategy, to the original AROA. The four different improvement strategies not only improve the performance of the original AROA, but also enhance the optimisation capability significantly. Figure 3 shows the flowchart of the IAROA algorithm. The steps of the IAROA algorithm are shown below:

Step 1. Set the population size *N*, maximum Iterations *t*_max_, variable dimension *dim*, execute EDO, and update the initial solution with Equation (17);

Step 2. According to the fitness value, all candidate solutions are arranged from good to worse, and the placement of the elite solutions are renewed to acquire the initialised candidate solutions;

Step 3. Calculate the fitness value *F* of every candidate solution, record the optimum fitness value *f_best_* and the corresponding optimal individual *x_best_*;

Step 4. Use Equation (14) to refresh all candidate solutions;

Step 5. Execute DLH to renew the candidate solution locations using Equations (18) and (19) for these solutions;

Step 6. Compare the fitness values of the solution before performing DLH with the solution after performing the operation and choose the individual corresponding to the smaller fitness value to renew the present candidate solution position;

Step 7. Judge whether the maximum iterations *t*_max_ have been achieved. If yes, carry out Step 14, or, if not, continue to carry out Step 8;

Step 8. Perform CDICP to renew the candidate solution locations using Equations (20) and (21) for these solutions;

Step 9. The fitness values of the solution before the perturbation are compared with the solution fitness values after the perturbation, and the location of the current candidate solution is renewed by selecting the location corresponding to the smaller fitness value;

Step 10. Renew all candidate solutions with Equation (15);

Step 11. The PAS is executed to refresh the candidate solution locations by using Equations (22) and (23) for these solutions;

Step 12. If the fitness value of the existing optimum solution is smaller than the optimum value *f_best_*, then use the fitness value of the existing optimal solution as the new optimum value *f_best_*, and at the same time, make the current optimum solution ***x****_best_* as the new optimum individual;

Step 13. Judge whether the maximum iterations *t*_max_ have been achieved. If yes, carry out Step 14, or, if not, go back to Step 4;

Step 14. Export the optimum solution location ***x****_best_* and its fitness value *f_best_*.

Corresponding to the above procedures, Algorithm 1 gives the pseudo-code of the IAROA algorithm. The flow diagram of IAROA is depicted in Figure 3.

**Algorithm 1:** IAROA algorithm

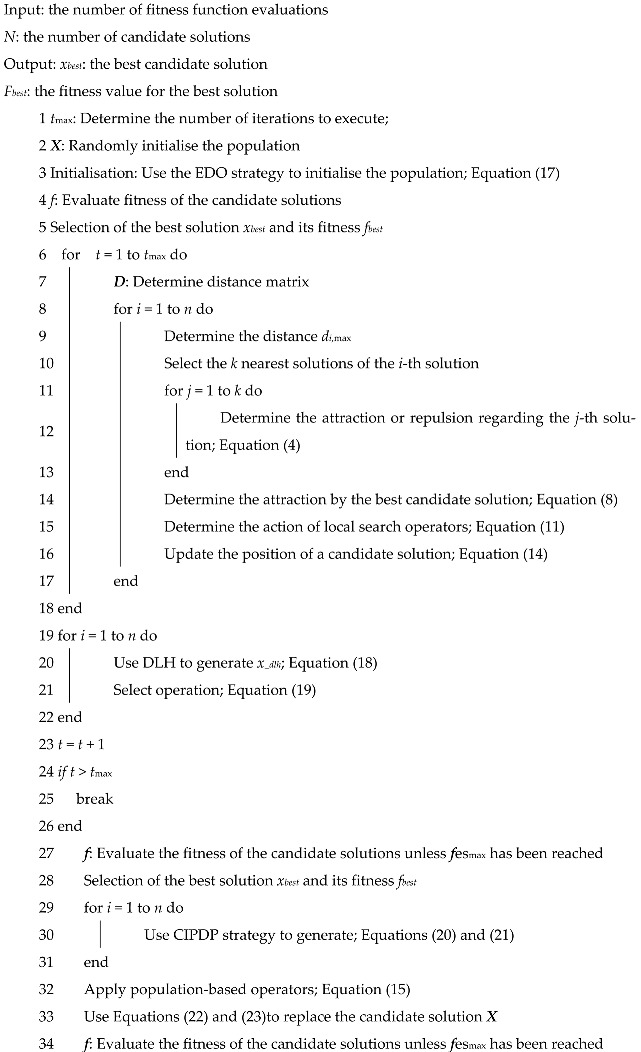



### 2.3. Time Complexity of the IAROA Algorithm

The computational complexity of IAROA is affected by the population size n, the individual dimensions and the maximum iterations. The procedures relevant to this value in IAROA include the population initialisation O(INI), the position retrieval formula O(PRF) in IAROA, the population-based operations O(PBO) of IAROA, the elite dynamic opposite learning strategy O(EDO), the Cauchy distribution inverse cumulative perturbation strategy O(CDICP), the dimension learning-based hunting search strategy O(DLH), and pheromone-adjusted strategy O(PAS). The specific computational procedure is as follows:

(24) $$\begin{array}{cc} O(IAROA) & =O\left(\mathrm{INI}\right)+O\left(PUF\right)+O\left(PBO\right)+O\left(EDO\right)+O\left(CDICP\right)+O\left(DLH\right)+O\left(PAS\right)\\ & =O(nd+nd)+\left(O\left(Tnd\right)+O\left(Tnd\right)+O\left(Tnd\right)+O\left(Tnd\right)+O\left(0.3Tnd\right)\right)/2\\ & =O(nd+2.15Tnd) \end{array}$$

### 2.4. Test Functions and Comparison Algorithms

In this section, fifteen intelligent optimisation algorithms, including AROA, are simulated and the results are evaluated to assess the performance of IAROA using the 30/50/100 dimensional CEC2017 test set. There are four major groups: the first group mainly selects intelligent algorithms that have been cited with high frequency in experimental applications, such as Marine Predators Algorithm (MPA, 2020) [29], Equilibrium Optimizer (EO, 2020) [30], Sparrow Search Algorithm (SSA, 2020) [31], and Grey Wolf Optimizer (GWO, 2014) [32]; and the second group mainly focuses on the ones that raised in the recent years as better intelligent optimisation algorithms: Archimedes Optimization Algorithm (AOA, 2020) [33], African Vulture Optimisation Algorithm (AVOA, 2021) [34], Dung Beetle Optimizer (DBO, 2022) [35], and Nutcracker Optimisation Algorithm (NOA, 2023); and the third group is mainly selected to be high-performance as well as highly competitive in the CEC competitions, such as LSAHDE-cnEpSin [36] and LSHADE-SPACMA [37]; the fourth group mainly selects the classical algorithm, Particle Swarm Optimisation Algorithm (PSO, 1995) [38] with the improvement algorithms of the three representative ones: the SRPSO [39], the XPSO [40], and the TAPSO [41]. The parameter settings of the algorithms employed in the experiment are presented in Table 1.

### 2.5. Analysis of Optimisation Results Under the CEC2017 Test Set

The IEEE International Conference on Evolutionary Computation (IEEE CEC) is a seminal conference in the field of evolutionary computation that fairly assesses the optimisation performance of algorithms by holding optimisation competitions. In these competitions, all novel evolutionary algorithms and population-based algorithms are tested using a uniform single-objective benchmark problem.

CEC2017 is a competition held in 2017, the core of which is to provide a set of test function sets for multimodal optimisation problems to assess and to contrast a variety of evolutionary algorithmic performance designs. The test function set mainly consisted of 29 test functions (the F2 has been officially deleted due to stability issues). The test set consists of four types:

(1) Single-peak shift-rotation functions (F1 and F3) have a global unique optimal solution and are ideal tools for inspecting algorithm development capabilities.
(2) Multi-peak shift-rotation functions (F4–F10) have multiple local optimum solutions and are suitable for testing the discovery ability of the algorithms.
(3) Hybrid functions (F11–F20) enable a comprehensive assessment of the algorithm’s ability to trade off between development and discovery due to their complex mathematical spatial properties.
(4) Composite functions (F21–F30) incorporate the hybrid function as a basic function.

In all the comparison tests, to make a reasonable comparison of the properties of the algorithms, we set up a uniform group of base parameter configurations: population scale *n* set to 100, maximum iterations *T* set to 1000, upper bound *ub* set to 100, and lower bound *lb* set to −100. Every algorithm was operated separately for *m* = 30, and the results were counted in the table.

#### 2.5.1. Optimisation Accuracy Analysis

Tables S1–S3 exhibits the experimental outcomes of Mean, std, *p*-value [42], and MR for IAROA and its contrasting algorithms after 30 separate operations on the CEC2017 test set prepared with setup dimensions of 30, 50, and 100, respectively, where the mean of the optimum performer on each function is highlighted in black and bold. Tables S1–S3 have been included in the Supplementary Material.

Combining the experimental results in Tables S1–S3, firstly, for the single-peak shift functions F1 and F3, IAROA’s search accuracy and std are significantly better than the original AROA in all three dimensions. For function F1, IAROA ranks third, second, and fifth in all three dimensions, and its overall performance is slightly inferior to that of the two competition-winning algorithms or MPA. For function F3, IAROA ranks first in all three dimensions.

In the tests of the multi-peak shift rotation function F4–F10, IAROA achieved 4, 6, and 7 first places in 30, 50, and 100 dimensions, respectively. In all three dimensions, the overall performance of IAROA has a more pronounced improvement compared to the original AROA, indicating that the DLH and the CDICP can sustain the large-scale exploration of space by the population in the early part of the iteration and the diversity of the population in the later part of the iteration, which leads to a significant improvement in the stability of IAROA. In the 30-dimensional experiment, IAROA ranks third on function F8, slightly inferior to LSHADE_SPACMA and LSHADE_cnEpSin, and still has room for improvement. As the dimensionality increases, the solving capability of IAROA improves remarkably, indicating that it performs well in handling complicated high-dimensional problems.

In the tests of hybrid functions F11–F20, IAROA achieved 9, 6, and 4 first places on 30D, 50D, and 100D, respectively. On 30D, IAROA does not perform as well as the two competitive ranking algorithms and the MPA algorithm on function F12, though it ranks first in the rest of the function tests. When the dimension is raised to 100D, the performance and stability of the IAROA algorithm are deficient, especially in the test of function F19, where IAROA ranks only fifth, and its performance metrics lag behind those of the two competition-winning algorithms, NOA and the original AROA algorithm.

In the test scenarios of composite functions F21–F30, the IAROA algorithm achieves 5, 8, and 9 first places under 30, 50, and 100D, respectively. In all three dimensions, IAROA has a significant advantage over the original AROA algorithm in terms of solution accuracy and std. In the 30D function test, IAROA’s metrics do not perform as well as the LSHADE_SPACMA algorithm or the LSHADE_cnEpSin algorithm on three functions. And in functions F26 and F27, IAROA’s mean and std are not as good as the MPA algorithm. When the dimension is raised to 50D, IAROA underperforms only on functions F25 and F26, while ranking first in the rest of the functions tested. In the 100D function F28, IAROA ranks second, behind the LSHADE_SPACMA algorithm. However, in the other function tests, IAROA outperforms all the algorithms involved in the comparison in terms of mean, best, and std. This is a good indication that when dealing with composite function problems, IAROA is less affected by dimensionality changes than the other compared algorithms. It can maintain competitive optimisation performance even when the function dimension increases and the computational process becomes more complex.

So as to assess whether IAROA has a remarkable enhancement in optimisation performance compared to the other compared algorithms, the Wilcoxon rank sum test [43] was adopted for statistically analysing the results. This test is suitable for judging whether the difference between two algorithms is significant or not. According to the principle of statistics, the null hypothesis can be rejected and the difference is considered significant when the *p*-value is less than 0.05, a criterion derived from the literature [44]. By contrasting the statistical examination outcomes in Tables S1–S3, we discover that IAROA significantly outperforms the other compared algorithms on most of the tested functions, with a *p*-value well below 0.05. Only on a very small number of functions is there no remarkable discrepancy between IAROA and the individual algorithms in terms of the solving results. Therefore, it can be drawn that IAROA is substantially different from the other comparative algorithms in terms of optimisation outcomes, and its optimisation finding is substantially better.

#### 2.5.2. Convergence Analysis

**Figure 4:**
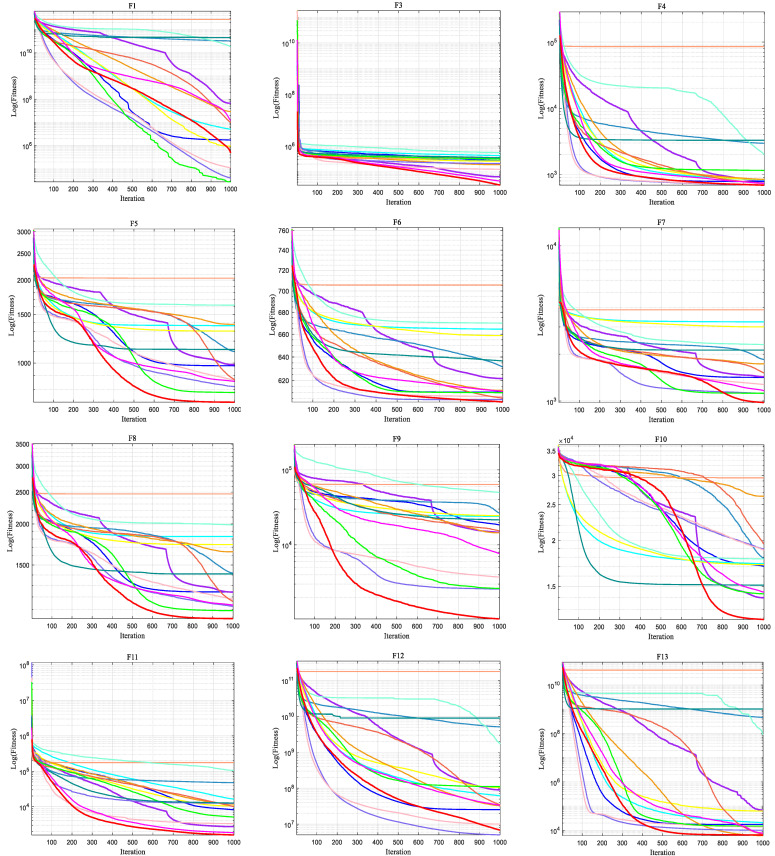

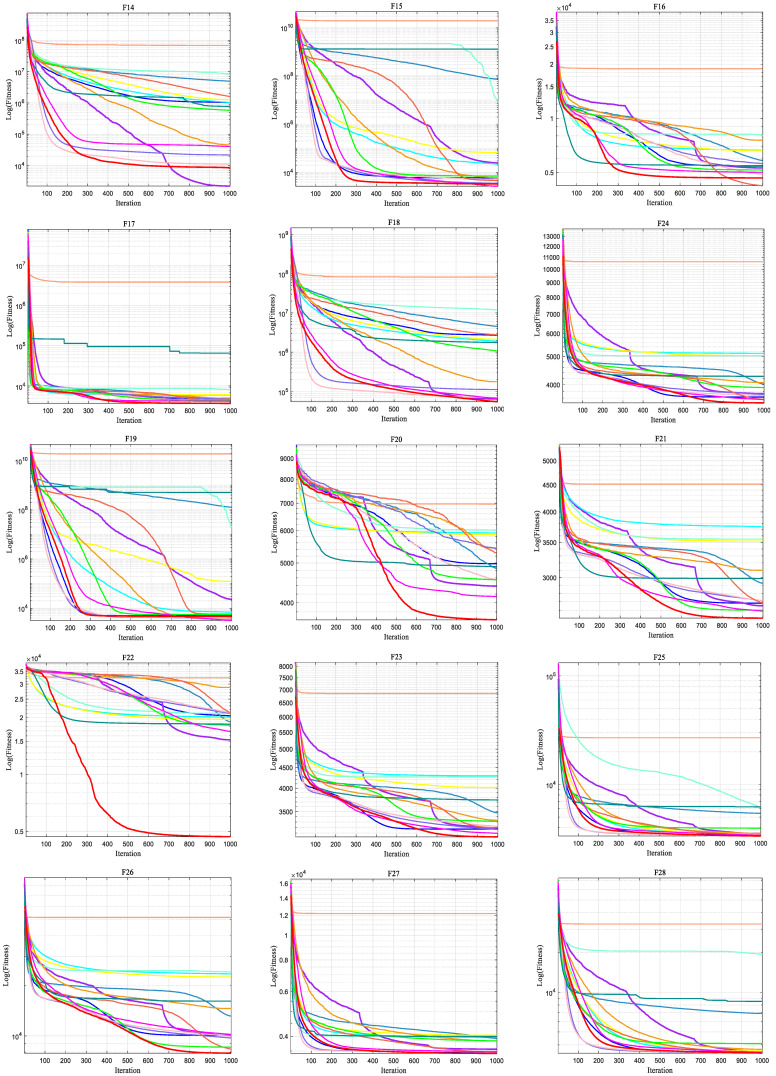

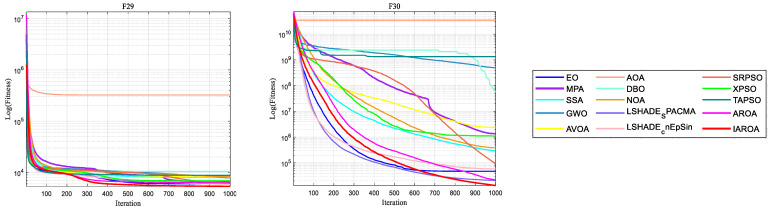
Convergence curves of IAROA with other algorithms for solving the 100D CEC2017.

Mean and std can only display the optimisation precision and stability of the algorithm. However, the convergence speed of the algorithm during the iteration procedure can only be revealed from the convergence curves. Figure 4 displays the mean of IAROA versus the other contrasting algorithms in the 100D CEC2017, with the horizontal coordinate being the iterations and the vertical coordinate being the logarithm of the fitness.

Figure 4 presents the convergence curves of IAROA with other comparison algorithms in the 100D CEC2017 test set environment. By observing this figure, it can be seen that in most of the function tests, IAROA shows fast convergence throughout the search process and achieves the lowest convergence accuracy, which fully demonstrates that IAROA has excellent global search capability. From the convergence curves of the multi-peak shift rotation functions F4–F10, IAROA is able to reach a stable convergence state for these tested functions quickly, which reflects the good convergence ability of the algorithm. In particular, the convergence curves of IAROA for the functions F3, F5, F7–F10, F20–F22 show a “steep” shape, which indicates that IAROA has a strong ability to remove local optima. In addition, for the hybrid functions (F11–F20) and the composite functions (F21–F30), although the convergence precision of IAROA on F12–F16, F19, and F28 is only slightly improved compared with the AROA, there is still the problem of falling into local optimums, which leads to the problem of premature convergence.

#### 2.5.3. Box Plot Analysis

A box-and-line diagram is a statistical diagram used to present the spread of digits [45]. It features and characterises data through five critical statistics—maximum value, minimum value, median, upper quartile, and lower quartile. In boxplots, data spots that are outside the whiskers (i.e., outside the upper and lower quartiles) are treated as outliers, and these outliers are typically highlighted with specific marker symbols (e.g., circles or asterisks).

**Figure 5:**
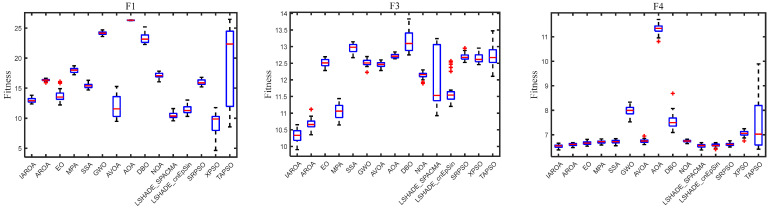

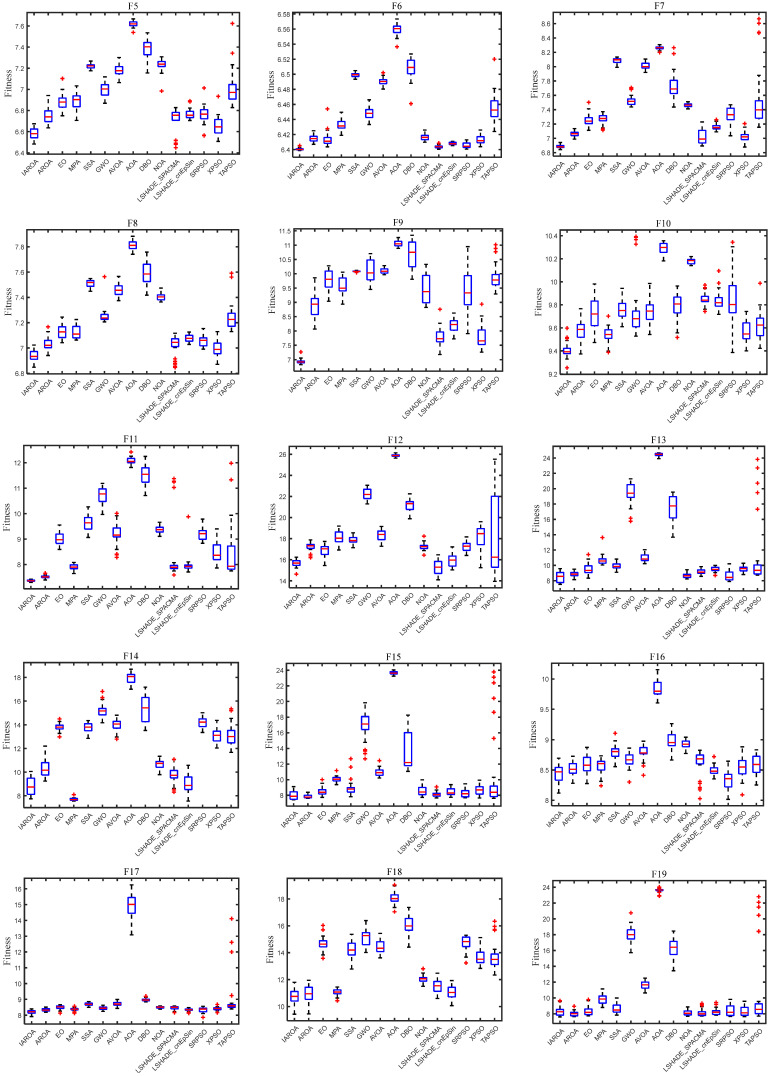

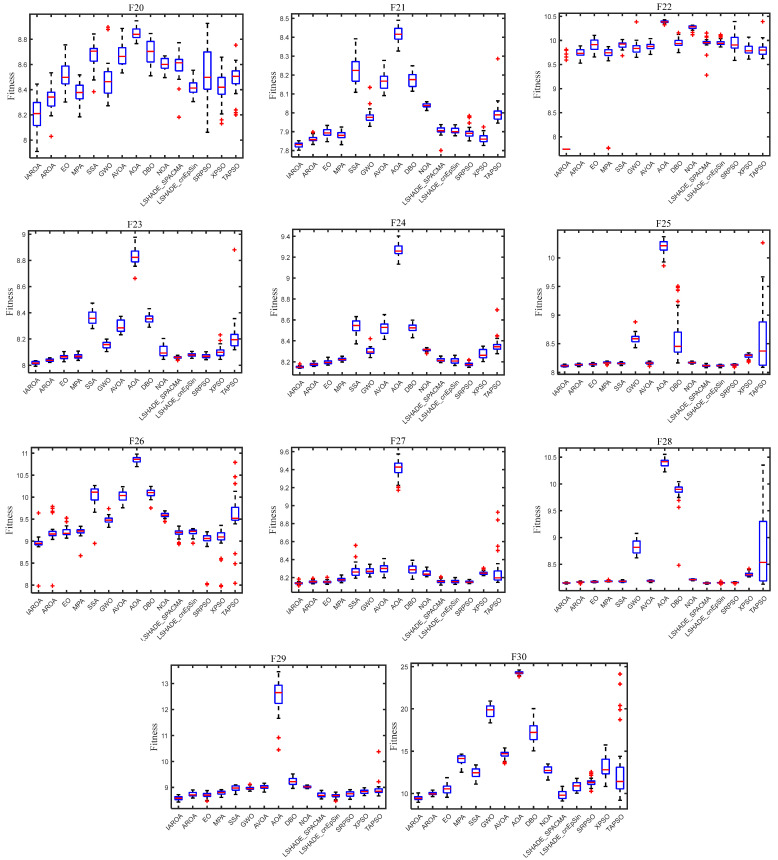
Boxplot of IAROA versus other algorithms solving the 100-dimensional CEC2017.

In order to better understand the data distribution of IAROA and each comparison algorithm’s outcome on the function solution, box-and-line plots were used to statistically analyse the results, as shown in Figure 5. Figure 5 presents the box-and-line plots of IAROA and each comparison algorithm for the 100D condition of the CEC2017 test set. Although the IAROA box is longer, the distribution of outcomes is wider on some functions. However, in most of the functions, the boxplot box is narrower and the median line is lower, indicating that the optimisation results have a concentrated data distribution, less fluctuation, and superior stability.

Figure 5 presents a radar plot of the IAROA algorithm’s ranking against the other contrasting algorithms, where red shading denotes the IAROA algorithm and the rest of the colours denote the contrasting algorithms. According to the properties of radargrams, the less shadowing area of the colour region signifies the superior performance of this algorithm. It is obvious from the diagram that the shadow area of the IAROA is the smallest in comparison with the other contrasting algorithms, so it can be concluded that IAROA has the best performance.

### 2.6. Ablation Experiment

**Figure 6:**
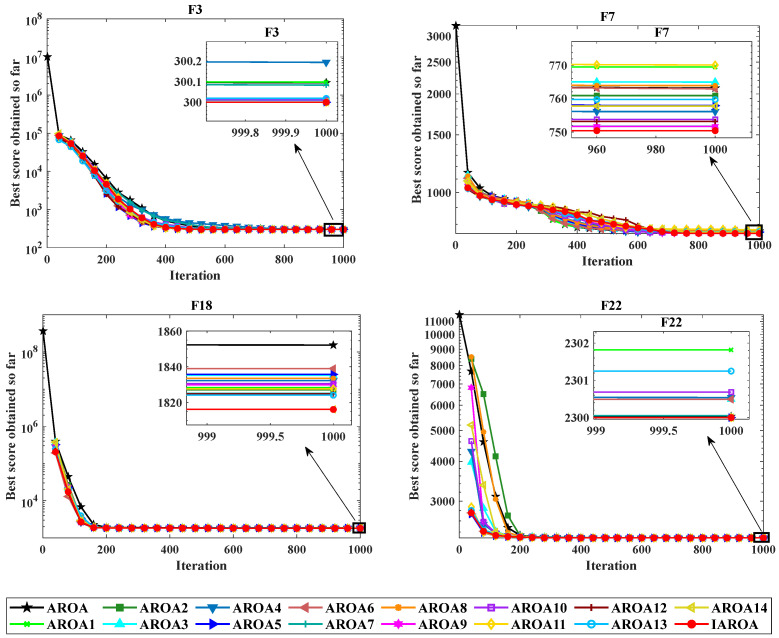
Line graph of the results of the partial ablation experiments.

In this section, in order to verify whether the four strategies added will have an impact on the original AROA algorithm, the mean value is used as a criterion to carry out ablation experiments on AROA with respect to these four strategies. Among them, Table S4 shows the different variants formed by AROA under the effect of the four different strategies; Table S5 presents the results obtained from the ablation experiments. Figure 6 presents a line graph of the results of some of the ablation experiments. By analysing the data in Table S5, it can be found that the performance of the original AROA is improved after adding different strategies to it. Specifically, the overall performance of the AROA variant with three strategies is better than that of the AROA variant with only two strategies; and the overall performance of the AROA variant with all four strategies is not only better than that of the AROA variant with two strategies, but also better than that of the AROA variant with three strategies, which is an outcome that fully proves that the gradual addition of strategies effectively improves the performance of the original AROA. Tables S4 and S5 have been included in the Supplementary Material.

## 3. Practice

Engineering design optimisation problems are designed to solve real-life problems by optimising economic indicators and related parameters, and they belong to the type of constrained optimisation problems that are very challenging in the real world. This type of problem is difficult to solve because of the complexity of the objective function and the existence of a large number of constraints. At the same time, the higher the number of design variables and constraints, the higher the computational complexity of the engineering problem. In this study, six engineering design problems with varying degrees of complexity are selected, which allow for a more comprehensive comparative test of algorithm performance and stability. Twelve algorithms, including AROA, were chosen for the experiments and compared with IAROA. When solving these engineering problems, the population size of all the optimisation algorithms is set to 50, the maximise number of iterations is set to 500, and the number of independent runs is set to 30, and the best, mean, worst, and standard deviation of the design results are used as the indexes for evaluating the solving ability of each algorithm. The code for the engineering design can be obtained from this link: https://github.com/jiangziwei9621/IAROA (accessed on 25 August 2025).

### 3.1. Industrial Chemical Processes

#### Alkylation Unit

**Figure 7:**
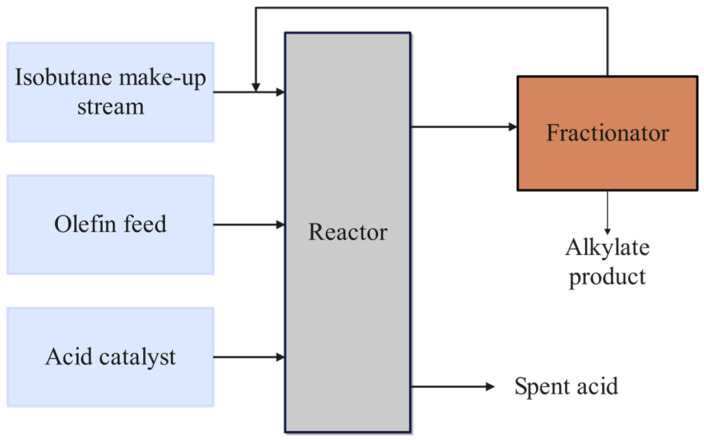
Schematic diagram of the Alkylation unit.

The major purpose of the Alkylation unit is to maximise the octane of the olefin feed in an acidic environment [46], so as to maximise the profit. This target function focuses on the Alkylation output and consists of 7 variables and 14 inequality constraints. The process flow diagram of the simplified Alkylation unit is depicted in Figure 7. The specific operation is illustrated below:

Minimise:

$$f\left(x\right)=0.035x_{1}x_{6}+1.715x_{1}+10.0x_{2}+4.0565x_{3}-0.063x_{3}x_{5}$$

Subject to:

$$\begin{array}{l} g_{1}\left(x\right)=0.0059553571x_{6}^{2}x_{1}+0.88392857x_{3}-0.1175625x_{6}x_{1}-x_{1}\leq 0,\\ g_{2}\left(x\right)=1.1088x_{1}+0.1303533x_{1}x_{6}-0.0066033x_{1}x_{6}^{2}-x_{3}\leq 0,\\ g_{3}\left(x\right)=6.66173269x_{6}^{2}-56.596669x_{4}+172.39878x_{5}-10,000-191.20592\leq 0,\\ g_{4}\left(x\right)=1.08702x_{6}-0.03762x_{6}^{2}+0.32175x_{4}+56.85075-x_{5}\leq 0,\\ g_{5}\left(x\right)=0.006198x_{3}x_{4}x_{7}+2462.3121x_{2}-25.125634x_{2}x_{4}-x_{3}x_{4}\leq 0,\\ g_{6}\left(x\right)=161.18996x_{3}x_{4}+5000.0x_{2}x_{4}-489,510.0x_{2}-x_{3}x_{4}x_{7}\leq 0,\\ g_{7}\left(x\right)=0.33x_{7}+44.333333-x_{5}\leq 0,\\ g_{8}\left(x\right)=0.022556x_{5}-1.0-0.007595x_{7}\leq 0,\\ g_{9}\left(x\right)=0.00061x_{3}-1.0-0.0005x_{1}\leq 0,\\ g_{10}\left(x\right)=0.819672x_{1}-x_{3}+0.819672\leq 0,\\ g_{11}\left(x\right)=24,500.0x_{2}-250.0x_{2}x_{4}-x_{3}x_{4}\leq 0,\\ g_{12}\left(x\right)=1020.4082x_{2}x_{4}+1.2244898x_{3}x_{4}-100,000x_{2}\leq 0,\\ g_{13}\left(x\right)=6.25x_{1}x_{6}+6.25x_{1}-7.625x_{3}-100,000\leq 0,\\ g_{14}\left(x\right)=1.22x_{3}-x_{1}x_{6}-x_{1}+1.0\leq 0. \end{array},$$

where:

$$1000\leq x_{1}\leq 2000,0\leq x_{2}\leq 100,2000\leq x_{3}\leq 4000,0\leq x_{4}\leq 100,0\leq x_{5}\leq 100,0\leq x_{6}\leq 20,0\leq x_{7}\leq 200.$$

From Table 2, the best and mean of IAROA are both optimum, while the best derived by IAROA is up to the theoretical optimum, which indicates that IAROA has strong competitiveness in the field of industrial chemical process. In addition, the std of IAROA is much smaller than that of other algorithms, which indicates that IAROA has high reliability and robustness compared with other comparative algorithms in solving the problem of optimal operation of an Alkylation plant. From Table 3, the optimal solution vector (2000.0000, 0.0000, 2576.4003, 0.0000, 58.1607, 1.2600, 41.2298), corresponding to the optimal fitness value, is −4529.119739.

### 3.2. Mechanical Engineering Issues

#### 3.2.1. Speed Reducer

**Figure 8:**
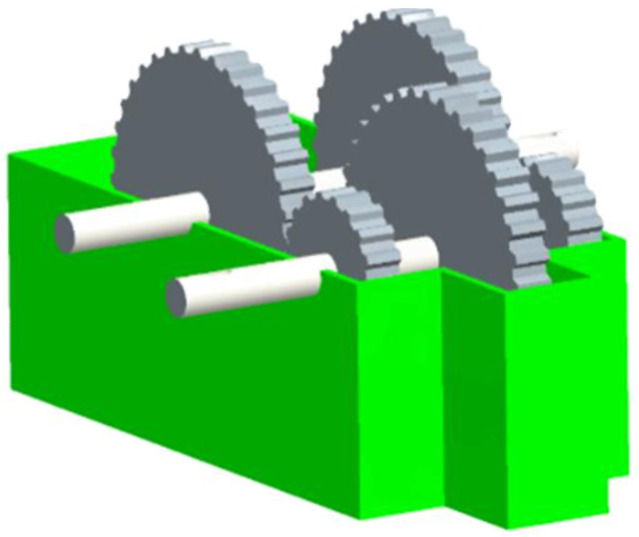
Schematic diagram of the Speed Reducer.

**Figure 9:**
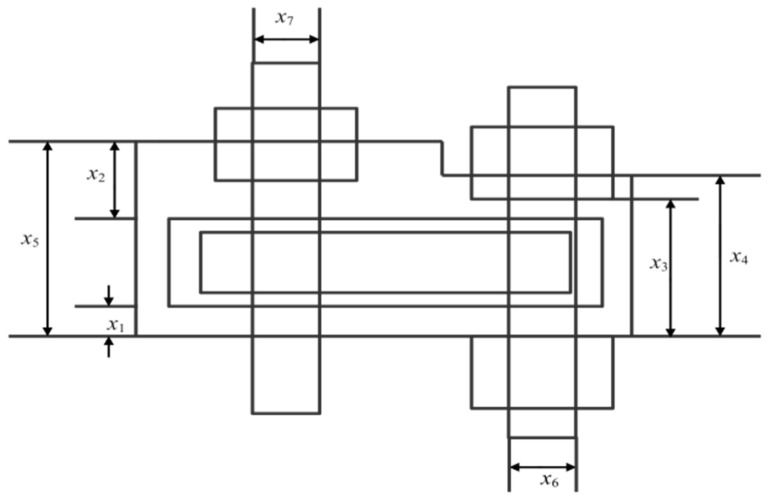
Schematic diagram of Speed Reducer parameters.

Speed Reducer is a gear between an aeroplane engine and a screw propeller [47], which is one of the essential components of a mechanical transmission system, whose structure is shown schematically in Figure 8. The design optimisation problem of the gearbox refers to the minimisation of the total quality of the gearbox on the basis of meeting the functional requirements of decreasing the rotational speed and raising the torque. The relevant parameters involved include face width $x_{1}$, tooth module $x_{2}$, number of pinion teeth $x_{3}$, length of the first shaft between bearings $x_{4}$, length of the second shaft between bearings $x_{5}$, diameter of the first shaft $x_{6}$, and diameter of the second shaft $x_{7}$, as shown in Figure 9. The specific operation is illustrated below:

Minimise:

$$\begin{array}{l} f\left(x\right)=0.7854x_{2}^{2}x_{1}\left(14.9324x_{3}-43.0934+3.3333x_{3}^{2}\right)\\ +0.7854\left(x_{5}x_{7}^{2}+x_{4}x_{6}^{2}\right)-1.508x_{1}\left(x_{7}^{2}+x_{6}^{2}\right)+7.477\left(x_{7}^{3}+x_{6}^{3}\right) \end{array}$$

Subject to:

$$\begin{array}{l} g_{1}\left(x\right)=-x_{1}x_{2}^{2}x_{3}+27\leq 0,\\ g_{2}\left(x\right)=-x_{1}x_{2}^{2}x_{3}^{2}+397.5\leq 0,\\ g_{3}\left(x\right)=-x_{2}x_{6}^{4}x_{3}x_{4}^{-3}+1.93\leq 0,\\ g_{4}\left(x\right)=-x_{2}x_{7}^{4}x_{3}x_{5}^{-3}+1.93\leq 0,\\ g_{5}\left(x\right)=10x_{6}^{-3}\sqrt{16.91\times 10^{6}+{\left(745x_{4}x_{2}^{-1}x_{3}^{-1}\right)}^{2}}-1100\leq 0,\\ g_{6}\left(x\right)=10x_{7}^{-3}\sqrt{157.5\times 10^{6}+{\left(745x_{5}x_{2}^{-1}x_{3}^{-1}\right)}^{2}}-850\leq 0,\\ g_{7}\left(x\right)=x_{2}x_{3}-40\leq 0, \end{array}$$

$$\begin{array}{l} g_{8}\left(x\right)=-x_{1}x_{2}^{-1}+5\leq 0,\\ g_{9}\left(x\right)=x_{1}x_{2}^{-1}-12\leq 0,\\ g_{10}\left(x\right)=1.5x_{6}-x_{4}+1.9\leq 0,\\ g_{11}\left(x\right)=1.1x_{7}-x_{5}+1.9\leq 0, \end{array}$$

where:

$$2.6\leq x_{1}\leq 3.6,0.7\leq x_{2}\leq 0.8,17\leq x_{3}\leq 28,7.3\leq x_{4},x_{5}\leq 8.3,2.9\leq x_{6}\leq 3.9,5\leq x_{7}\leq 5.5$$

As can be seen from Table 4, IAROA, SSA, DBO, and TAPSO all seek the best optimal and reach the theoretical optimum, but the mean of IAROA is the smallest, which manifests that the entirety quality of solutions obtained by IAROA is high. And the std of IAROA is much smaller than the other algorithms, which indicates that IAROA has strong stability and robustness compared with other comparative algorithms in solving the speed reducer problem. As shown in Table 5, IAROA, DBO, SSA, and TAPSO have similar optimal solution vectors (3.500000, 0.700000, 17.000000, 7.300000, 7.715320, 3.350541, 5.286654), correlating to an optimal fitness value of 2994.424465757.

#### 3.2.2. Industrial Refrigeration System

Modern industry continues to refine the process of development, electronics, pharmaceuticals, and many other industries for the refrigeration environment of the parameters of the control precision requirements. At the same time, the industrial refrigeration system, as a result of industrial energy consumption as a whole, accounted for a high proportion, thus becoming the key to the optimal allocation of energy and precise control. The optimum design problem for industrial refrigeration systems [48,49] can be defined as a nonlinear inequality-bound optimisation question. The question contains 14 parameters (*x*_1_~*x*_14_) and 15 nonlinear restraints (*g*_1_~*g*_14_). The specific operation is illustrated below:

Minimise:

$$\begin{array}{l} f\left(x\right)=63,098.88x_{2}x_{4}x_{12}+5441.5x_{2}^{2}x_{12}+115,055.5x_{2}^{1.664}x_{6}+6172.27x_{2}^{2}x_{6}\\ +63,098.88x_{1}x_{3}x_{11}+5441.5x_{1}^{2}x_{11}+115,055.5x_{1}^{1.664}x_{5}+6172.27x_{1}^{2}x_{5}\\ +140.53x_{1}x_{11}+281.29x_{3}x_{11}+70.26x_{1}^{2}+281.29x_{1}x_{3}+281.29x_{3}^{2}\\ +14,437x_{8}^{1.8812}x_{12}^{0.3424}x_{10}x_{14}^{-1}x_{1}^{2}x_{7}x_{9}^{-1}+20,470.2x_{7}^{2.893}x_{11}^{0.316}x_{1}^{2} \end{array}$$

Subject to:

$$\begin{array}{l} g_{1}\left(x\right)=1.524x_{7}^{-1}\leq 0,\\ g_{2}\left(x\right)=1.524x_{8}^{-1}\leq 0,\\ g_{3}\left(x\right)=0.07789x_{1}-2x_{7}^{-1}x_{9}-1\leq 0,\\ g_{4}\left(x\right)=7.05305x_{9}^{-1}x_{1}^{2}x_{10}x_{8}^{-1}x_{2}^{-1}x_{14}^{-1}-1\leq 0,\\ g_{5}\left(x\right)=0.0833x_{13}^{-1}x_{14}-1\leq 0,\\ g_{6}\left(x\right)=47.136x_{2}^{0.333}x_{10}^{-1}x_{12}-1.33x_{8}x_{13}^{2.1195}+62.08x_{13}^{2.1195}x_{12}^{-1}x_{8}^{0.2}x_{10}^{-1}-1\leq 0,\\ g_{7}\left(x\right)=0.0477x_{10}x_{8}^{1.8812}x_{12}^{0.3424}-1\leq 0,\\ g_{8}\left(x\right)=0.0488x_{9}x_{7}^{1.893}x_{11}^{0.316}-1\leq 0,\\ g_{9}\left(x\right)=0.0099x_{1}x_{3}^{-1}-1\leq 0,\\ g_{10}\left(x\right)=0.0193x_{2}x_{4}^{-1}-1\leq 0,\\ g_{11}\left(x\right)=0.0298x_{1}x_{5}^{-1}-1\leq 0,\\ g_{12}\left(x\right)=0.056x_{2}x_{6}^{-1}-1\leq 0,\\ g_{13}\left(x\right)=2x_{9}^{-1}-1\leq 0,\\ g_{14}\left(x\right)=2x_{10}^{-1}-1\leq 0,\\ g_{15}\left(x\right)=x_{12}x_{11}^{-1}-1\leq 0, \end{array}$$

where:

$$0.001\leq x_{i}\leq 5,i=1,\ldots ,14$$

As can be seen from Table 6, IAROA’s best is optimal and reaches the theoretical optimal value, and IAROA’s mean is the smallest in contrast to the others, which means that the overall quality of solutions obtained by IAROA is significantly improved, i.e., IAROA has a more competitive edge in the field of mechanical engineering. However, the std of MPA is smaller than that of IAROA, which indicates that the stability and robustness of IAROA are not sufficient in solving industrial refrigeration problems. From Table 7, the optimal solution vector of IAROA is (0.001, 0.001, 0.001, 0.001, 0.001, 0.001, 0.001, 0.001, 0.001, 1.524, 5.00, 2.00, 0.001, 0.001, 0.001, 0.007293, 0.087556), and the corresponding optimal fitness value is 0.032213002.

#### 3.2.3. Welded Beam Design

**Figure 10:**
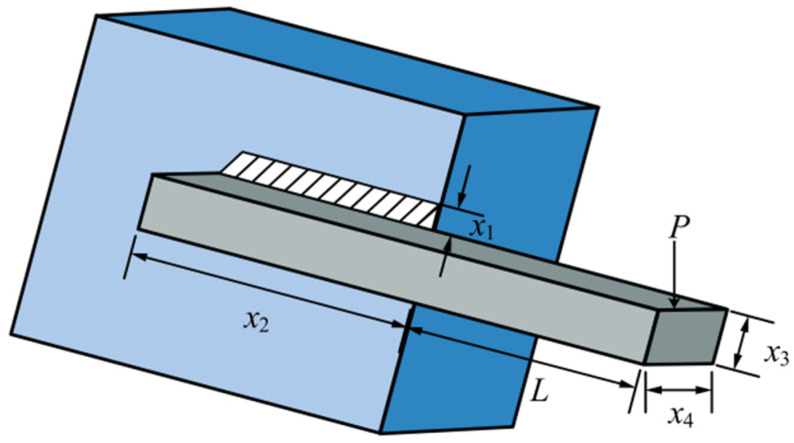
Schematic diagram of a welded beam.

Welded beam problem [50,51], as a typical case of classical optimal design in the field of mechanical and structural engineering, has key practical applications in many aspects such as steel structure design, mechanical manufacturing, and architectural engineering etc. The aim of this problem is to find the solution with the lowest cost under the constraints of specific conditions. The schematic is shown in Figure 10. The optimisation of the welded beam involves four associated design parameters (*t*,*h*,*l*,*b*) and four nonlinear inequality constraints (*g*_1_~*g*_5_). The specific mathematical model is shown below:

$$Consider\;X=[x_{1},x_{2},x_{3},x_{4}]=[t,h,l,b]$$

Minimise:

$$f\left(x\right)=0.04811x_{3}x_{4}\left(x_{2}+14\right)+1.10471x_{1}^{2}x_{2}$$

Subject to:

$$\begin{array}{l} g_{1}\left(x\right)=x_{1}-x_{4}\leq 0,\\ g_{2}\left(x\right)=\delta \left(x\right)-\delta_{\max}\leq 0,\\ g_{3}\left(x\right)=P-P_{c}\left(x\right)\leq 0,\\ g_{4}\left(x\right)=\tau \left(x\right)-\tau_{\max}\leq 0,\\ g_{5}\left(x\right)=\sigma \left(x\right)-\sigma_{\max}\leq 0, \end{array}$$

where:

$$0.125\leq x_{1}\leq 2,0.1\leq x_{2},x_{3}\leq 10,0.1\leq x_{4}\leq 2$$

Other parameters are computed as follows:

$$\begin{array}{l} \tau =\sqrt{{\tau^{\prime }}^{2}+{\tau^{\prime \prime }}^{2}+2\tau^{\prime }\tau^{\prime \prime }\frac{x_{2}}{2R}},\tau^{\prime \prime }=\frac{RM}{J},\tau^{\prime }=\frac{P}{\sqrt[{}]{2}x_{2}x_{1}},\\ R=\sqrt{\frac{x_{2}^{2}}{4}+{\left(\frac{x_{1}+x_{3}}{2}\right)}^{2}},J=2\left(\frac{x_{2}^{2}}{4}+{\left(\frac{x_{1}+x_{3}}{2}\right)}^{2}\right)\sqrt{2}x_{1}x_{2},\\ \sigma \left(x\right)=\frac{6PL}{x_{3}^{2}x_{4}},\delta \left(x\right)=\frac{6PL^{3}}{Ex_{3}^{2}x_{4}},P_{c}\left(x\right)=\frac{4.013Ex_{3}x_{4}^{3}}{6L^{2}}\left(1-\frac{x_{3}}{2L}\sqrt{\frac{E}{4G}}\right),\\ L=14\mathrm{in},P=6000\mathrm{lb},E=30\times 10^{6}\mathrm{psi},\sigma_{\max}=30,000\mathrm{psi}, \end{array}$$

$$\tau_{\max}=13,600\mathrm{psi},G=12\times 10^{6}\mathrm{psi},\delta_{\max}=0.25\mathrm{in}.$$

As indicated in Table 8, the best, mean, worst, and std of IAROA are all optimal as compared to other contrasting algorithms, and the std of IAROA is much smaller than the other compared algorithms, which suggests that IAROA has much stability and robustness for solving the design problem of welded beams, i.e., IAROA has a strong competitiveness in the field of mechanical engineering. From Table 9, the optimal solution vector of IAROA is (0.19883230722, 3.33736529865, 9.19202432248, 0.19883230722) and the corresponding optimal fitness value is 1.67021772630, which demonstrates that IAROA possesses a superior performance in addressing the actual engineering issues.

#### 3.2.4. Robot Gripper Problem

**Figure 11:**
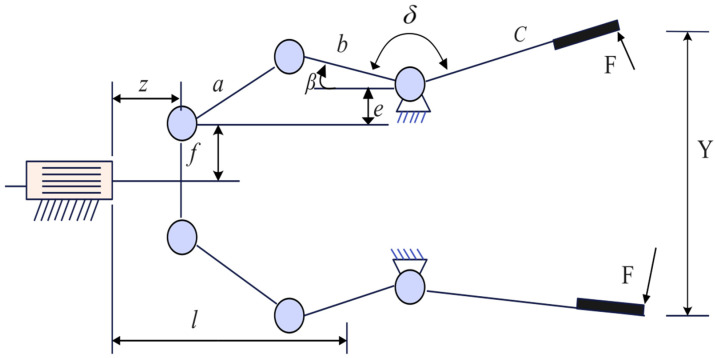
Schematic diagram of the robot gripper.

Grippers are essential end-effectors in robotics and are widely used in various fields [52]. The optimal design of the gripper is a classic nonlinear engineering robot gripper problem designed to enhance the gripping precision of the robot. The schematic of the gripper is shown in Figure 11. The problem has four design parameters (*a*,*b*,*c*,*e*,*f*,*l*,*δ*) and three nonlinear restraints (*g*_1_~*g*_7_). The specific operation is illustrated below:

$$Consider\;X=[x_{1},x_{2},x_{3},x_{4},x_{5},x_{6},x_{7}]=[a,b,c,e,f,l,\delta ]$$

Minimise:

$$f\left(x\right)=-\min_{z}F_{k}\left(x,z\right)+\max_{z}F_{k}\left(x,z\right)$$

Subject to:

$$\begin{array}{l} g_{1}\left(x\right)=-Y_{\min}+y\left(x,Z_{\max}\right)\leq 0,\\ g_{2}\left(x\right)=-y\left(x,Z_{\max}\right)\leq 0,\\ g_{3}\left(x\right)=Y_{\max}-y\left(x,0\right)\leq 0,\\ g_{4}\left(x\right)=y\left(x,0\right)-Y_{G}\leq 0,\\ g_{5}\left(x\right)=l^{2}+e^{2}-{\left(a+b\right)}^{2}\leq 0,\\ g_{6}\left(x\right)=b^{2}-{\left(a-e\right)}^{2}-{\left(l-Z_{\max}\right)}^{2}\leq 0,\\ g_{7}\left(x\right)=Z_{\max}-l\leq 0, \end{array}$$

where:

0≤e≤50,100≤c≤200,10≤f,a,b≤150,1≤δ≤3.14,100≤l≤300.

Other parameters are computed as follows:

$$\begin{array}{l} \alpha =\cos^{-1}\left(\frac{a^{2}+g^{2}-b^{2}}{2ag}\right)+\varphi ,g=\sqrt{e^{2}+{\left(z-l\right)}^{2}},\\ \beta =\cos^{-1}\left(\frac{b^{2}+g^{2}-a^{2}}{2bg}\right)-\varphi ,\varphi =\tan^{-1}\left(\frac{e}{l-z}\right), \end{array}$$

$$\begin{array}{l} y\left(x,z\right)=2\left(f+e+c\sin\left(\beta +\delta \right)\right),F_{k}=\frac{Pb\sin\left(\alpha +\beta \right)}{2c\cos\left(\alpha \right)},Y_{\min}=50,\\ Y_{\max}=100,Y_{G}=150,Z_{\max}=100,P=100. \end{array}$$

From Table 10, the best, mean, worst, and std of IAROA are all optimum and reach the theoretical optimum, which demonstrates that the solution performance of IAROA is superior, and that IAROA has strong dependability and robustness in tackling the robot gripper matter. From Table 11, the optimum solution vector of IAROA is (150.0000, 149.8828, 200.0000, 0.0000, 149.9999, 100.9430, 2.2974), and the corresponding optimum fitness value is 2.543790469.

#### 3.2.5. Himmelblau Function

The nonlinear planning problem was generalised by Himmelblau in the solution of a mechanical design issue [53], where the machine manufacturing problem is a minimisation problem with an optimisation goal equation, such that the consumption of steel is minimised. The problem includes five relevant design variables and six nonlinear inequality constraints. The specific mathematical model is shown below:

Minimise:

$$f\left(x\right)=5.3578547x_{3}^{2}+0.8356891x_{1}x_{5}+37.293239x_{1}-40792.141$$

Subject to:

$$\begin{array}{l} g_{1}\left(x\right)=-G1\leq 0,\\ g_{2}\left(x\right)=G1-92\leq 0,\\ g_{3}\left(x\right)=90-G2\leq 0,\\ g_{4}\left(x\right)=G2-110\leq 0,\\ g_{5}\left(x\right)=20-G3\leq 0,\\ g_{6}\left(x\right)=G3-25\leq 0, \end{array}$$

where:

$$\begin{array}{l} 78\leq x_{1}\leq 102,33\leq x_{2}\leq 45,27\leq x_{3}\leq 45,27\leq x_{4}\leq 45,27\leq x_{5}\leq 45,\\ G1=85.334407+0.0056858x_{2}x_{5}+0.0006262x_{1}x_{4}-0.0022053x_{3}x_{5},\\ G2=80.51249+0.0071317x_{2}x_{5}+0.0029955x_{1}x_{2}+0.0021813x_{3}^{2},\\ G3=9.300961+0.0047026x_{3}x_{5}+0.00125447x_{1}x_{3}+0.0019085x_{3}x_{4}. \end{array}$$

As can be seen from Table 12, IAROA, AROA, SSA, and DBO all find the smallest best and reach the theoretical optimum. However, IAROA has the same mean and worst as best, while its standard deviation std is much smaller than that of AROA, SSA, and DBO. This indicates that the solutions obtained by IAROA are more stable and of better quality, and show strong stability and robustness in solving the Himmelblau nonlinear programming problem. From Table 13, the optimal solution vector derived by IAROA is (78.0000000, 33.0000000, 29.9952560, 45.0000000, 36.7758129) and the corresponding optimal fitness value is −30,665.538671784.

## 4. Conclusions and Expectations

In this study, we produce an improved Attraction–Repulsion Optimisation Algorithm based on multi-strategy, denoted as IAROA. Firstly, based on the original AROA, four improvement strategies, EDO, CDICP, DLH, and PAS, are introduced, aiming to increase the population diversity and the precision of optimisation search in AROA. Secondly, this paper selects four types of metaheuristic optimisation algorithms, namely classical algorithms, algorithms with outstanding performance in recent years, algorithms on the top list of the CEC competition, and improved classical algorithms, as the objects of comparison, and adopts the comprehensive CEC2017 test set to construct an experimental environment to examine the performance of IAROA. The experimental outcomes show that IAROA has excellent optimisation performance. Finally, in order to further evaluate the performance of IAROA in solving real-world problems, six real-world engineering constrained optimisation problems with varying degrees of complexity are also selected for experiments in this paper. The outcomes show that IAROA demonstrates significant superiority and robustness in engineering practice.

In our future work, we will apply the proposed IAROA algorithm to solve optimisation problems in more fields by selecting suitable strategies based on the characteristics of different optimisation problems or combining them with other algorithms, such as energy prediction [54] and path planning.

## Figures and Tables

**Table 1:** Parameter settings for some of the comparison algorithms.

Arithmetic	Parameterisation
AROA	*c* = 0.95, *f*_*r*_1__ = 0.15, *f*_*r*_2__ = 0.15, *p*_1_ = 0.6, *p*_2_ = 0.8, *e_f_* = 0.4, *tr*1 = 0.9, *tr*2 = 0.85, *tr*3 = 0.9
IAROA	*c* = 0.98, *f*_*r*_1__ = 0.15, *f*_*r*_2__ = 0.15, *p*_1_ = 0.6, *p*_2_ = 0.8, *e_f_* = 0.2, *tr*1 = 0.9, *tr*2 = 0.85, *tr*3 = 0.9
MPA	*FADs* = 0.2, *P* = 0.5
EO	*a*_1_ = 2, *a*_2_ = 1, *GP* = 0.5
SSA	*P_percent* = 0.2
AVOA	*p*_1_*=* 0.6, *p*_2_ *=* 0.4, *p*_3_ *=* 0.6, *alpha* = 0.8, *betha* = 0.2, *gamma* = 2.5
AOA	*MOA_max* = 1, *MOA_min* = 0.2, *µ* = 0.5, *a* = 5
NOA	*Alpha* = 0.05, *Pa*2 = 0.2, *Prb* = 0.2
DBO	*P_percent* = 0.2,
LSHADE_SPACMA	*L_Rate* = 0.8, *num_prbs* = 30
LSHADE_cnEpSin	*µF* = 0.5, *µCR* = 0.5, *H = 5*, *pb* = 0.4, *ps* = 0.5
SRPSO	*ϖ*_min_ = 0.5, *ϖ*_max_ = 1.05, *c*1 = 1.49445, *c*2 = 1.49445
XPSO	*elite_ratio* = 0.5
TAPSO	*tatio* = 0.5

**Table 2:** Comparative results of optimal operation of Alkylation units.

Arithmetic	Best	Mean	Worst	Std
IAROA	−4529.119739	−4529.118781	−4529.104906	0.002852721
AROA	−4529.119724	−4333.335038	321.1617524	891.8735161
EO	−4528.336676	1.85265E+12	5.55795E+13	1.01474E+13
MPA	−4529.119356	−4528.975121	−4526.301432	0.532510698
SSA	−4520.736764	1.75014E+17	5.24861E+18	9.5825E+17
GWO	−4507.618354	1.22554E+18	1.41438E+19	3.43021E+18
AVOA	−4339.874892	1.71088E+17	5.1306E+18	9.36702E+17
AOA	5.25688E+17	9.81415E+20	8.48024E+21	1.90575E+21
DBO	−4526.325004	9.45899E+18	2.47028E+20	4.49258E+19
NOA	−4491.895714	−4238.302978	−2996.590641	357.074788
SRPSO	242.7751343	7.29395E+15	5.65979E+16	1.58177E+16
XPSO	307.5008681	1.72755E+15	1.22294E+16	3.29314E+15
TAPSO	95.76530005	5.64566E+19	1.63764E+21	2.98727E+20

**Table 3:** Optimal design for optimal operation of Alkylation units.

Arithmetic	Norm							Best
	*x*_1_	*x*_2_	*x*_3_	*x*_4_	*x*_5_	*x*_6_	*x*_7_	
IAROA	2000.0000	0.0000	2576.4003	0.0000	58.1607	1.2600	41.2298	−4529.1197
AROA	2000.0000	0.0000	2576.2814	0.0000	58.1602	1.2595	41.0656	−4529.1197
EO	2000.0000	0.0000	2520.7881	0.0000	57.9240	1.0236	41.0957	−4528.3367
MPA	2000.0000	0.0000	2576.6928	0.0000	58.1620	1.2613	41.6599	−4529.1194
SSA	2000.0000	0.0000	2393.8182	0.0000	57.3913	0.5062	39.3992	−4520.7368
GWO	2000.0000	0.0000	2846.6975	0.0000	59.3727	2.5429	45.0267	−4507.6184
AVOA	1978.1237	0.0000	3245.5427	0.0000	61.5237	5.2220	52.0448	−4339.8749
AOA	1370.2034	0.0000	2000.0000	0.0000	60.4692	2.9016	118.3750	5.257E+17
DBO	2000.0000	0.0000	2471.1994	0.0000	57.7148	0.8180	40.0673	−4526.3250
NOA	2000.0000	0.0000	2898.0294	0.0000	59.6468	2.8014	45.6220	−4491.8957
SRPSO	1546.5284	99.9386	2489.1299	89.1439	91.0258	5.2212	141.4753	242.7751
XPSO	1255.2243	83.1251	2122.5650	88.9856	93.2456	12.9129	145.2599	307.5009
TAPSO	1212.0246	99.9832	2000.0000	92.2010	94.3667	14.2142	148.5907	95.7653

**Table 4:** Speed Reducer comparison results.

Arithmetic	Best	Mean	Worst	Std
IAROA	2994.424465757	2994.424465757	2994.424465757	8.27383E−13
AROA	2994.424465796	2994.428042917	2994.462009308	0.007962375
EO	2994.424467407	2994.940560954	3007.389939194	2.394794727
MPA	2994.432990414	2994.464360154	2994.552827818	0.032564995
SSA	2994.424465757	2994.424465761	2994.424465816	0.000000013
GWO	3005.081581813	3012.799534176	3027.361758088	6.172417742
AVOA	2994.485468413	3001.600561104	3012.584810106	4.779087615
AOA	3089.654773885	3168.842990181	3227.788857380	43.609182558
DBO	2994.424465757	3059.233600655	3202.582182010	61.362527570
NOA	2994.424478453	2994.424560776	2994.424773818	0.000072466
SRPSO	2994.424502755	2994.424588091	2994.424802393	0.000068117
XPSO	3010.085574331	3023.140078015	3028.962368880	4.128647703
TAPSO	2994.424465757	3084.211466321	4302.119178611	259.168749641

**Table 5:** Optimal design of Speed Reducer.

Arithmetic	Norm							Best
	*x*_1_	*x*_2_	*x*_3_	*x*_4_	*x*_5_	*x*_6_	*x*_7_	
IAROA	3.500000	0.700000	17.000000	7.300000	7.715320	3.350541	5.286654	2994.424466
AROA	3.500000	0.700000	17.000000	7.300000	7.715320	3.350541	5.286654	2994.424466
EO	3.500000	0.700000	17.000000	7.300000	7.715320	3.350541	5.286654	2994.424467
MPA	3.500003	0.700000	17.000002	7.300000	7.715535	3.350545	5.286656	2994.432990
SSA	3.500000	0.700000	17.000000	7.300000	7.715320	3.350541	5.286654	2994.424466
GWO	3.503537	0.700000	17.000000	7.467445	7.851442	3.352099	5.293569	3005.081582
AVOA	3.500000	0.700000	17.000000	7.302007	7.717221	3.350545	5.286655	2994.485468
AOA	3.600000	0.700000	17.000000	8.300000	8.300000	3.375762	5.329721	3089.654774
DBO	3.500000	0.700000	17.000000	7.300000	7.715320	3.350541	5.286654	2994.424466
NOA	3.500000	0.700000	17.000000	7.300000	7.715320	3.350541	5.286654	2994.424478
SRPSO	3.500000	0.700000	17.000000	7.300000	7.715320	3.350541	5.286654	2994.424503
XPSO	3.501907	0.700329	17.002960	7.715093	7.885609	3.357101	5.292655	3010.085574
TAPSO	3.500000	0.700000	17.000000	7.300000	7.715320	3.350541	5.286654	2994.424466

**Table 6:** Comparative results for industrial refrigeration.

Arithmetic	Best	Mean	Worst	Std
IAROA	0.032213002	0.036734092	0.156321137	0.022643996
AROA	0.032232241	1.56031E+14	9.36186E+14	3.54861E+14
EO	0.032220672	9.36186E+13	9.36186E+14	2.85657E+14
MPA	0.033126578	0.045639262	0.070884567	0.009299800
SSA	0.03221639	2.18444E+14	9.36186E+14	4.02732E+14
GWO	0.04034639	2.19203E+14	9.42846E+14	4.04133E+14
AVOA	0.042434402	6.86537E+14	9.36187E+14	4.21075E+14
AOA	5.098532627	4.67991E+14	1.682E+15	5.97876E+14
DBO	0.044763203	7.79872E+14	3.38785E+15	7.14765E+14
NOA	0.032670028	0.049593042	0.078531071	0.014427310
SRPSO	0.050958848	9.36186E+13	9.36186E+14	2.85657E+14
XPSO	0.499612786	4.74863E+13	1.42459E+15	2.60093E+14
TAPSO	0.032226552	1.01416E+15	9.12181E+15	1.91438E+15

**Table 7:** Optimal design of industrial refrigeration.

Arithmetic	Norm						
	*x*_1_	*x*_2_	*x*_3_	*x*_4_	*x*_5_	*x*_6_	*x*_7_
IAROA	0.001000	0.001000	0.001000	0.001000	0.001000	0.001000	1.524000
AROA	0.001000	0.001000	0.001001	0.001000	0.001000	0.001001	1.524129
EO	0.001000	0.001000	0.001000	0.001003	0.001000	0.001000	1.524003
MPA	0.001000	0.001238	0.001015	0.001109	0.001088	0.001144	1.524008
SSA	0.001000	0.001000	0.001000	0.001000	0.001000	0.001000	1.524001
GWO	0.001000	0.001129	0.001123	0.001195	0.003270	0.001181	1.524347
AVOA	0.001000	0.001000	0.001000	0.002072	0.001000	0.002144	1.524001
AOA	0.001000	0.001000	0.001000	1.679435	0.169433	1.727573	5.000000
DBO	0.001000	0.001000	0.001000	0.001000	0.001000	0.001000	1.524000
NOA	0.001000	0.001000	0.001000	0.001000	0.001000	0.001000	1.524038
SRPSO	0.001001	0.001072	0.001718	0.001499	0.001305	0.001031	1.545509
XPSO	0.000000	0.001293	0.002440	0.089629	0.877626	0.094048	2.783794
TAPSO	0.001000	0.001000	0.001000	0.001000	0.001000	0.001000	1.524000
**Norm**							**Best**
***x*** **_8_**	***x*** **_9_**	***x*** **_10_**	***x*** **_11_**	***x*** **_12_**	***x*** **_13_**	***x*** **_14_**	
1.524000	5.000000	2.000000	0.001000	0.001000	0.007293	0.087556	0.032213002
1.524278	4.999994	2.000334	0.001010	0.001010	0.007326	0.087951	0.032232241
1.524041	4.999927	2.000003	0.001000	0.001000	0.007292	0.087537	0.032220672
1.524005	5.000000	2.000139	0.001019	0.001016	0.007348	0.088213	0.033126578
1.524004	4.999997	2.000009	0.001000	0.001000	0.007292	0.087543	0.03221639
1.524921	4.985041	2.013069	0.005317	0.005052	0.015337	0.183778	0.04034639
1.524001	4.933572	2.328689	0.007956	0.005692	0.017713	0.212635	0.042434402
2.277239	3.416799	2.374662	0.001000	0.001000	0.002130	0.008816	5.098532627
1.524000	5.000000	5.000000	0.002072	0.002072	0.015851	0.190286	0.044763203
1.528605	4.970139	2.015543	0.001003	0.001000	0.007315	0.087398	0.032670028
1.534080	4.744826	4.191221	0.005251	0.004998	0.021267	0.252935	0.050958848
3.399624	2.524434	2.778828	0.149176	0.030801	0.024767	0.204583	0.499612786

**Table 8:** Comparison results of welded beams.

Arithmetic	Best	Mean	Worst	Std
IAROA	1.6702177263	1.670217726	1.670217726	3.81709E−13
AROA	1.6702177264	1.670509295	1.674240477	0.000792958
EO	1.6702179939	1.671599635	1.697186403	0.004923826
MPA	1.6702185592	1.670226507	1.670242132	0.000006517
SSA	1.6702178503	1.779812944	2.262797186	0.148506619
GWO	1.6712384796	1.676213570	1.697783459	0.005098199
AVOA	1.6736509403	1.758871592	1.816849475	0.051331019
AOA	1.9338394264	2.339173265	2.742053530	0.191872482
DBO	1.6702306649	1.754227932	1.902804769	0.072558174
NOA	1.6702197253	1.670244842	1.670346272	2.93581E−05
SRPSO	1.6702187308	1.670411051	1.673644428	0.000631020
XPSO	1.6702177263	1.670240491	1.670717093	9.15251E−05
TAPSO	1.6702178263	2.014143679	5.453527824	0.713119231

**Table 9:** Optimal design of welded beams.

Arithmetic	Norm				Best
	*x*_1_	*x*_2_	*x*_3_	*x*_4_	
IAROA	0.19883230722	3.33736529865	9.19202432248	0.19883230722	1.67021772630
AROA	0.19883230718	3.33736529935	9.19202432278	0.19883230722	1.67021772640
EO	0.19883231922	3.33736478516	9.19202537600	0.19883232586	1.67021799390
MPA	0.19883198519	3.33737379241	9.19202575982	0.19883230055	1.67021855920
SSA	0.19883233882	3.33736492830	9.19202356992	0.19883233999	1.67021785030
GWO	0.19821666038	3.35150485273	9.19247421285	0.19883104333	1.67123847960
AVOA	0.19937272345	3.33255027593	9.17231614750	0.19968767089	1.67365094030
AOA	0.12500000000	5.50547860699	10.00000000000	0.19594970586	1.93383942640
DBO	0.19883154106	3.33734606069	9.19215184203	0.19883171314	1.67023066490
NOA	0.19883190377	3.33737354767	9.19202287301	0.19883253486	1.67021972530
SRPSO	0.19883170350	3.33737819322	9.19202411741	0.19883233679	1.67021873080
XPSO	0.19883230722	3.33736529865	9.19202432248	0.19883230722	1.67021772630
TAPSO	0.19883231440	3.33736530865	9.19202379059	0.19883233024	1.67021782630

**Table 12:** Himmelblau function comparison results.

Arithmetic	Best	Mean	Worst	Std
IAROA	−30,665.538671784	−30,665.538671784	−30,665.538671784	8.9876841E−12
AROA	−30,665.538671784	−30,665.538661737	−30,665.538518081	3.5086010E−05
EO	−30,665.538671783	−30,665.524005855	−30,665.315217247	0.042360026
MPA	−30,665.538566739	−30,665.538095716	−30,665.537366849	0.000325091
SSA	−30,665.538671784	−30,665.538647089	−30,665.538285515	9.1271724E−05
GWO	−30,662.724520364	−30,657.279460554	−30,641.165298064	4.795902492
AVOA	−30,665.537797521	−30,661.905410462	−30,609.047187836	10.961911294
AOA	−30,573.492828232	−29,587.801638904	−28,883.198992723	405.704030987
DBO	−30,665.538671784	−30,659.523848894	−30,491.114101749	31.815822115
NOA	−30,665.537122546	−30,665.529190299	−30,665.495723935	0.011777849
SRPSO	−30,665.535954249	−30,659.819894304	−30,495.698990493	30.997549900
XPSO	−30,644.778298218	−30,612.308790981	−30,529.963003225	23.667554290
TAPSO	−30,665.538671784	−30,607.422932739	−29,893.249053707	189.359224254

**Table 13:** Optimal design of Himmelblau function.

Arithmetic	Norm					Best
	*x*_1_	*x*_2_	*x*_3_	*x*_4_	*x*_5_	
IAROA	78.0000000	33.0000000	29.9952560	45.0000000	36.7758129	−30,665.5386718
AROA	78.0000000	33.0000000	29.9952560	45.0000000	36.7758129	−30,665.5386718
EO	78.0000000	33.0000000	29.9952560	45.0000000	36.7758129	−30,665.5386718
MPA	78.0000000	33.0000000	29.9952565	45.0000000	36.7758122	−30,665.5385667
SSA	78.0000000	33.0000000	29.9952560	45.0000000	36.7758129	−30,665.5386718
GWO	78.0000000	33.0000000	30.0066260	45.0000000	36.7629096	−30,662.7245204
AVOA	78.0000000	33.0000015	29.9952616	45.0000000	36.7757988	−30,665.5377975
AOA	78.0000000	33.0000000	30.2498287	45.0000000	36.9272888	−30,573.4928282
DBO	78.0000000	33.0000000	29.9952560	45.0000000	36.7758129	−30,665.5386718
NOA	78.0000000	33.0000005	29.9952607	45.0000000	36.7758137	−30,665.5371225
SRPSO	78.0000184	33.0000045	29.9952664	45.0000000	36.7757842	−30,665.5359542
XPSO	78.0136130	33.0715995	30.0817785	44.9701113	36.6528608	−30,644.7782982
TAPSO	78.0000000	33.0000000	29.9952560	45.0000000	36.7758129	−30,665.5386718

**Table 10:** Robot gripper comparison results.

Arithmetic	Best	Mean	Worst	Std
IAROA	2.543790469	2.589878551	3.036007330	0.097775114
AROA	2.543968130	2.876128644	3.651941325	0.303358096
EO	2.547173857	3.498080150	5.966433367	0.752739873
MPA	2.546031208	2.838837273	3.153007707	0.175297596
SSA	2.792611983	6.313794528	72.686677135	12.588411554
GWO	3.092374187	3.823935171	4.564037027	0.376625847
AVOA	2.591978421	4.156581140	6.767453071	0.931277604
AOA	3.705794959	5.111113547	16.101476709	2.264300497
DBO	2.552505703	4.789698560	8.338582727	1.435788140
NOA	3.061661302	3.444272265	3.781554059	0.175854864
SRPSO	3.354019627	3.983458789	4.941396283	0.351937577
XPSO	3.036056335	4.425171790	5.368783114	0.512379516
TAPSO	2.644273848	6.579609477	49.625244808	9.957733009

**Table 11:** Optimal design of robotic gripper.

Arithmetic	Norm							Best
	*x*_1_	*x*_2_	*x*_3_	*x*_4_	*x*_5_	*x*_6_	*x*_7_	
IAROA	150.0000	149.8828	200.0000	0.0000	149.9999	100.9430	2.2974	2.543790469
AROA	150.0000	149.8825	200.0000	0.0003	150.0000	100.9528	2.2978	2.543968130
EO	150.0000	149.8810	200.0000	0.0000	150.0000	101.1322	2.3172	2.547173857
MPA	149.9669	149.8394	200.0000	0.0094	149.1333	101.0302	2.2982	2.546031208
SSA	148.9886	141.6340	200.0000	7.0801	136.4111	110.8249	2.3244	2.792611983
GWO	149.8509	149.7731	199.1393	0.0000	25.6465	103.5958	1.6901	3.092374187
AVOA	150.0000	149.8724	200.0000	0.0000	150.0000	102.3170	2.3702	2.591978421
AOA	150.0000	98.6045	200.0000	50.0000	150.0000	129.6142	2.8907	3.705794959
DBO	150.0000	149.8779	200.0000	0.0000	136.4369	101.4306	2.2325	2.552505703
NOA	148.1264	139.7663	199.8558	7.6498	143.0177	125.3845	2.4155	3.061661302
SRPSO	149.9801	123.2550	184.1166	26.2918	149.4296	115.4428	2.7775	3.354019627
XPSO	143.0801	138.5940	191.5218	4.1952	15.9860	110.4247	1.6847	3.036056335
TAPSO	150.0000	142.5250	200.0000	7.3194	149.9913	103.5358	2.4202	2.644273848

## Data Availability

All data generated or analysed during this study were included in this published article.

## Supplementary Materials

The following supporting information can be downloaded at https://www.mdpi.com/article/10.3390/biomimetics10090628/s1, Figure S1: Line chart of various algorithms under 30-dimensional cec2017; Figure S2: Line chart of various algorithms under 50-dimensional cec2017; Figure S3: The boxplots of various algorithms under 30-dimensional cec2017; Figure S4: The boxplots of various algorithms under 50-dimensional cec2017; Figure S5: The radar images of various algorithms; Table S1: Comparison results of various algorithms under 30-dimensional cec2017; Table S2: Comparison results of various algorithms under 50-dimensional cec2017; Table S3: Comparison results of various algorithms under 100-dimensional cec2017; Table S4: The different variants of AROA under four different strategies; Table S5: Results of AROA ablation experiments based on four strategies.
