# Supplementary material for: IAROA: An Enhanced Attraction–Repulsion Optimisation Algorithm Fusing Multiple Strategies for Mechanical Optimisation Design
Source: Biomimetics (Basel). 2025 Sep 17;10(9):628. doi: 10.3390/biomimetics10090628 (PMC12467200; doi:10.3390/biomimetics10090628)
Supplement: Supplementary file 1 [file biomimetics-10-00628-s001.zip › biomimetics-3773413-supplementary.pdf]

Supplementary materials

Figures S1,S2 show the convergence graphs of IAROA and other algorithms on (30, 50, dimensions of CEC2017).

Figures S3,S4show the boxplots of IAROA and other algorithms on (30, 50 dimensions of CEC2017).

Figure S5 shows the radar images of IAROA and other novel high-performance algorithms.

Tables S 1, S 2, and S 3 show the experimental results of IAROA and other algorithms on the 30, 50, and 100 dimensions of CEC2017.

Tables S4 show the different variants of AROA under four different strategies.

Tables S5 show results of AROA ablation experiments based on four strategies.

All IAROA codes are available at this link (https://github.com/jiangziwei9621/IAROA)

Table S1. Comparison of results of IAROA with other algorithms (30-dimensional CEC2017).

F	Index	IAROA	AROA	EO	MPA	SSA	GWO	AVOA	AOA	DBO	NOA	LSHADE_ SPACMA	LSHADE_ cnEpSin	SRPSO	XPSO	TAPSO
1	Mean	2.2959E+02	2.7634E+03	4.0426E+03	3.9158E+03	3.4938E+03	1.1098E+09	3.6021E+03	4.4521E+10	2.7280E+06	2.1368E+03	**1.0000E+02**	1.0000E+02	3.1312E+03	1.6731E+03	5.4440E+08
	std	3.6027E+02	2.2157E+03	4.8410E+03	3.2176E+03	4.8219E+03	8.5515E+08	4.1298E+03	6.5484E+09	5.4994E+06	1.9822E+03	1.4211E-14	5.1873E-04	3.3226E+03	2.0382E+03	2.5412E+09
	P-value	-	2.3715E-10	1.3367E-05	7.3891E-11	3.0811E-08	3.0199E-11	4.4205E-06	4.4205E-06	3.3384E-11	1.7769E-10	5.1812E-12	3.0199E-11	8.8910E-10	2.6243E-03	1.3594E-07
	Rank	3	8	7	12	9	14	6	15	13	5	1	2	10	4	11
3	Mean	**3.0000E+02**	3.0009E+02	4.0065E+03	3.0051E+02	2.7855E+04	3.3261E+04	6.6835E+03	7.8117E+04	5.9636E+04	2.5421E+03	1.2112E+04	3.1602E+02	1.0596E+04	3.5880E+03	2.6307E+03
	std	2.6734E-06	4.9895E-02	1.4685E+03	7.1814E-01	7.7738E+03	9.4196E+03	3.3597E+03	9.4747E+03	2.0742E+04	6.5956E+02	3.1179E+04	7.9143E+01	3.7362E+03	1.9532E+03	2.7358E+03
	P-value	-	3.0199E-11	3.0199E-11	3.0199E-11	3.0199E-11	3.0199E-11	3.0199E-11	3.0199E-11	3.0199E-11	3.0199E-11	1.1051E-06	3.0199E-11	3.0199E-11	3.0199E-11	3.0199E-11
	Rank	1	4	9	5	12	13	10	15	14	7	2	3	11	8	6
4	Mean	**4.2591E+02**	4.4126E+02	4.9104E+02	4.8851E+02	4.9259E+02	5.5408E+02	4.9206E+02	1.1050E+04	5.4807E+02	4.7978E+02	4.5450E+02	4.6664E+02	4.9955E+02	5.3032E+02	9.7153E+02
	std	2.8376E+01	3.2268E+01	2.9798E+01	3.4858E+00	2.0784E+01	3.4160E+01	3.3199E+01	3.0068E+03	5.5850E+01	2.5419E+01	1.6220E+01	2.4476E+01	1.4156E+01	1.9111E+01	1.4984E+03
	P-value	-	1.0035E-03	2.6695E-09	3.0199E-11	8.1527E-11	3.0199E-11	2.6695E-09	2.6695E-09	3.0199E-11	4.1997E-10	1.1143E-03	5.5329E-08	3.0199E-11	3.0199E-11	5.0922E-08
	Rank	1	3	10	7	6	14	8	15	12	5	2	4	11	13	9
5	Mean	5.2584E+02	5.4676E+02	5.6211E+02	5.7664E+02	7.3189E+02	6.0546E+02	7.0096E+02	8.3253E+02	6.8764E+02	6.0381E+02	**5.2409E+02**	5.2639E+02	5.5127E+02	5.4441E+02	5.8367E+02
	std	7.0716E+00	1.3078E+01	1.9213E+01	1.6920E+01	5.5195E+01	4.3864E+01	4.4153E+01	3.0759E+01	4.8811E+01	1.1708E+01	5.7014E+00	5.5654E+00	1.8159E+01	1.2395E+01	2.2341E+01
	P-value	-	7.0881E-08	1.2057E-10	4.0772E-11	3.0199E-11	3.0199E-11	3.0199E-11	3.0199E-11	3.0199E-11	3.0199E-11	5.8945E-01	3.8710E-01	4.1997E-10	8.4848E-09	3.3384E-11
	Rank	2	5	7	8	14	10	13	15	12	11	1	3	6	4	9

6	Mean	**6.0000E+02**	6.0003E+02	6.0002E+02	6.0178E+02	6.4265E+02	6.0511E+02	6.4205E+02	6.6968E+02	6.2709E+02	6.0002E+02	6.0001E+02	6.0001E+02	6.0010E+02	6.0008E+02	6.0441E+02
	std	1.8599E-04	1.1158E-02	7.8283E-02	8.6665E-01	9.5021E+00	2.1209E+00	7.2315E+00	6.7726E+00	1.0689E+01	7.9127E-03	2.3869E-02	1.4399E-02	2.1341E-01	9.1184E-02	7.1464E+00
	P-value	-	3.0199E-11	3.3384E-11	3.0199E-11	3.0199E-11	3.0199E-11	3.0199E-11	3.0199E-11	3.0199E-11	3.0199E-11	3.1830E-01	7.6950E-08	3.0199E-11	3.0199E-11	3.0199E-11
	Rank	1	7	4	9	13	11	14	15	12	6	2	3	5	8	10
7	Mean	7.5493E+02	7.6971E+02	7.8961E+02	8.1349E+02	1.2004E+03	8.4850E+02	1.1305E+03	1.3390E+03	9.1803E+02	8.5210E+02	**7.4447E+02**	7.5843E+02	8.1189E+02	7.8213E+02	8.2562E+02
	std	5.8666E+00	1.2454E+01	1.7340E+01	1.9584E+01	9.9825E+01	4.6343E+01	1.0235E+02	4.7843E+01	6.4586E+01	1.4386E+01	4.4204E+00	5.8821E+00	4.5822E+01	2.0209E+01	5.6213E+01
	P-value	-	1.3853E-06	4.9752E-11	3.0199E-11	3.0199E-11	3.0199E-11	3.0199E-11	3.0199E-11	3.0199E-11	3.0199E-11	1.3111E-08	4.3584E-02	8.9934E-11	4.9980E-09	3.0199E-11
	Rank	2	4	6	8	14	10	13	15	12	11	1	3	7	5	9
8	Mean	8.2822E+02	8.4600E+02	8.6605E+02	8.7271E+02	9.7055E+02	8.7117E+02	9.4309E+02	1.0783E+03	9.8580E+02	8.9512E+02	8.2144E+02	8.2671E+02	8.5119E+02	8.4845E+02	8.7957E+02
	std	6.8559E+00	1.3528E+01	2.2145E+01	1.4068E+01	2.4502E+01	1.4564E+01	3.5552E+01	2.9177E+01	4.4638E+01	1.5611E+01	**6.2041E+00**	4.2822E+00	1.6316E+01	1.7383E+01	2.2422E+01
	P-value	-	3.3520E-08	5.4941E-11	3.0199E-11	3.0199E-11	4.9752E-11	3.0199E-11	3.0199E-11	3.0199E-11	3.0199E-11	2.6795E-04	2.5188E-01	5.4617E-09	9.0632E-08	3.0199E-11
	Rank	3	4	7	8	13	9	12	15	14	11	1	2	6	5	10
9	Mean	**9.0008E+02**	9.0341E+02	9.0666E+02	9.8693E+02	5.3089E+03	1.4795E+03	5.1862E+03	6.3637E+03	5.4642E+03	9.0041E+02	9.0036E+02	9.0073E+02	9.0182E+02	9.0366E+02	1.4873E+03
	std	1.8145E-01	3.3450E+00	7.5074E+00	4.2970E+01	5.5618E+02	5.0424E+02	8.0467E+02	8.0696E+02	2.3077E+03	3.5596E-01	5.1246E-01	7.7254E-01	1.3235E+00	3.5831E+00	6.2016E+02
	P-value	-	4.3702E-11	4.3702E-11	2.9284E-11	2.9284E-11	2.9284E-11	2.9284E-11	2.9284E-11	2.9284E-11	6.3950E-08	1.4389E-02	2.6656E-07	1.2912E-10	2.9265E-11	2.9284E-11
	Rank	1	6	8	9	13	11	12	15	14	3	2	4	5	7	10
10	Mean	**3.1703E+03**	3.6080E+03	4.6249E+03	3.7052E+03	5.3612E+03	3.9463E+03	5.3903E+03	7.2148E+03	5.4318E+03	5.7685E+03	3.5534E+03	3.4953E+03	3.9413E+03	3.8709E+03	4.3084E+03
	std	4.2785E+02	5.7853E+02	6.5961E+02	4.3945E+02	7.6039E+02	6.8446E+02	7.3586E+02	7.7682E+02	7.5782E+02	2.8929E+02	2.9140E+02	3.1133E+02	1.0005E+03	6.0500E+02	5.0910E+02
	P-value	-	2.4994E-03	1.4643E-10	5.6073E-05	3.0199E-11	1.0188E-05	3.3384E-11	3.3384E-11	4.9752E-11	3.0199E-11	3.1821E-04	1.1738E-03	1.1058E-04	1.1674E-05	2.0338E-09
	Rank	1	4	10	5	12	8	11	15	13	14	3	2	6	7	9
11	Mean	**1.1121E+03**	1.1269E+03	1.1561E+03	1.1467E+03	1.2713E+03	1.4878E+03	1.2481E+03	6.9145E+03	1.4995E+03	1.1809E+03	1.1503E+03	1.1573E+03	1.1914E+03	1.2018E+03	1.3285E+03
	std	1.0786E+01	2.6526E+01	3.4410E+01	1.8292E+01	4.6698E+01	4.3375E+02	6.1918E+01	2.7888E+03	1.7482E+02	2.1249E+01	2.2731E+01	3.0391E+01	4.3391E+01	4.3460E+01	5.2874E+02
	P-value	-	5.0912E-06	5.0723E-10	3.8202E-10	3.0199E-11	3.0199E-11	3.0199E-11	3.0199E-11	3.0199E-11	7.3891E-11	2.8716E-10	5.0723E-10	6.6955E-11	6.6955E-11	3.0199E-11
	Rank	1	2	6	3	12	13	11	15	14	7	4	5	8	9	10
12	Mean	1.5058E+04	4.7700E+04	2.5703E+05	1.3524E+04	8.3490E+05	2.7776E+07	2.3569E+06	1.0823E+10	3.5083E+07	4.8162E+04	**8.5477E+03**	1.2432E+04	4.8814E+05	5.3077E+05	1.7895E+08
	std	8.0917E+03	3.8336E+04	2.0842E+05	8.8144E+03	8.0283E+05	2.5124E+07	1.3667E+06	3.3704E+09	6.7607E+07	1.5305E+04	5.7779E+03	1.7733E+04	3.8827E+05	1.0336E+06	9.7047E+08
	P-value	-	3.5201E-07	6.0658E-11	3.0418E-01	3.0199E-11	3.0199E-11	3.0199E-11	3.0199E-11	3.0199E-11	5.4941E-11	5.8737E-04	1.3272E-02	3.0199E-11	8.8411E-07	7.6950E-08
	Rank	4	5	9	3	11	14	12	15	13	6	1	2	10	8	7
13	Mean	**1.3943E+03**	7.8203E+03	1.5538E+04	1.5587E+03	1.1106E+04	4.2985E+06	6.7164E+04	2.1842E+09	1.6091E+06	3.5325E+03	1.8933E+03	2.3493E+03	1.8605E+04	1.6115E+04	1.3400E+05

	std	2.4496E+02	6.5490E+03	1.6223E+04	7.0540E+01	1.0591E+04	1.7887E+07	3.2897E+04	1.7753E+09	2.8390E+06	5.4894E+02	3.0106E+02	9.4859E+02	1.7600E+04	2.6724E+04	6.0551E+05
	P-value	-	8.1527E-11	9.9186E-11	6.5183E-09	4.9752E-11	3.0199E-11	3.0199E-11	3.0199E-11	3.0199E-11	3.0199E-11	1.6947E-09	6.1210E-10	6.6955E-11	8.9934E-11	4.0772E-11
	Rank	1	6	8	2	9	13	12	15	14	5	3	4	10	7	11
14	Mean	**1.4196E+03**	1.4313E+03	2.4241E+04	1.4377E+03	2.6491E+04	3.6750E+05	4.3782E+04	5.3644E+05	1.0987E+05	1.4731E+03	1.4652E+03	1.4711E+03	1.2112E+04	1.3293E+04	2.5190E+04
	std	1.0104E+01	9.7392E+00	1.7322E+04	6.7522E+00	2.1323E+04	4.8104E+05	3.7873E+04	5.9011E+05	9.8667E+04	8.0370E+00	2.3149E+01	2.2765E+01	1.1741E+04	1.1260E+04	4.5079E+04
	P-value	-	1.5292E-05	3.0199E-11	1.1737E-09	3.0199E-11	3.0199E-11	3.0199E-11	3.0199E-11	3.0199E-11	3.0199E-11	3.0199E-11	3.0199E-11	3.0199E-11	3.0199E-11	3.0199E-11
	Rank	1	2	10	3	11	14	12	15	13	6	4	5	7	8	9
15	Mean	**1.5104E+03**	1.5774E+03	6.3837E+03	1.5518E+03	9.2029E+03	3.8526E+05	2.0785E+04	2.7485E+04	4.4244E+04	1.7226E+03	1.6320E+03	1.6741E+03	9.1539E+03	5.9671E+03	6.4314E+03
	std	5.0855E+00	8.7727E+01	4.2362E+03	1.2371E+01	9.7250E+03	6.9375E+05	1.2300E+04	1.3019E+04	5.9722E+04	3.5359E+01	6.9069E+01	9.9422E+01	8.7917E+03	4.3267E+03	6.5147E+03
	P-value	-	1.7769E-10	3.0199E-11	3.0199E-11	3.0199E-11	3.0199E-11	3.0199E-11	3.0199E-11	3.0199E-11	3.0199E-11	3.0199E-11	3.0199E-11	3.0199E-11	3.0199E-11	3.0199E-11
	Rank	1	3	9	2	11	15	12	14	13	6	4	5	10	8	7
16	Mean	**1.8965E+03**	2.1130E+03	2.2374E+03	2.0969E+03	2.8730E+03	2.3930E+03	2.8375E+03	4.2644E+03	2.9515E+03	2.2501E+03	2.0858E+03	1.8890E+03	2.1891E+03	2.2333E+03	2.5214E+03
	std	1.5323E+02	1.6951E+02	3.3669E+02	1.2972E+02	3.0410E+02	1.9846E+02	3.2822E+02	5.3598E+02	3.8370E+02	1.1049E+02	2.1117E+02	1.7132E+02	2.2841E+02	2.7424E+02	2.5186E+02
	P-value	-	1.6351E-05	6.2828E-06	1.0188E-05	3.0199E-11	1.3289E-10	3.3384E-11	3.3384E-11	3.6897E-11	1.1737E-09	3.5638E-04	8.6499E-01	5.4620E-06	1.1937E-06	6.6955E-11
	Rank	1	5	7	3	13	10	12	15	14	9	4	2	6	8	11
17	Mean	**1.7538E+03**	1.8170E+03	1.9166E+03	1.7908E+03	2.4643E+03	1.9909E+03	2.4121E+03	2.7391E+03	2.4241E+03	1.8189E+03	1.7776E+03	1.7843E+03	1.8495E+03	1.8499E+03	2.0981E+03
	std	3.3022E+01	7.1302E+01	1.3137E+02	4.1341E+01	2.8111E+02	1.5923E+02	2.3363E+02	3.5926E+02	2.1883E+02	2.5325E+01	2.0323E+01	3.7871E+01	8.0780E+01	7.4172E+01	2.0346E+02
	P-value	-	8.1975E-07	3.9648E-08	3.3242E-06	3.3384E-11	1.4643E-10	3.0199E-11	3.0199E-11	3.0199E-11	7.1186E-09	3.3242E-06	4.7445E-06	7.7725E-09	6.5183E-09	7.3891E-11
	Rank	1	5	9	4	14	10	12	15	13	6	2	3	7	8	11
18	Mean	**1.8266E+03**	1.8447E+03	2.5164E+05	1.8491E+03	3.3447E+05	6.4400E+05	6.6299E+05	4.9840E+06	4.9010E+05	2.6601E+03	1.9791E+03	2.0116E+03	2.5377E+05	1.5061E+05	2.5096E+05
	std	8.6659E+00	1.8492E+01	2.5079E+05	8.2383E+00	3.0309E+05	7.4323E+05	7.5961E+05	4.7596E+06	9.0709E+05	3.9162E+02	7.5308E+01	7.4913E+01	1.6945E+05	1.1633E+05	3.4522E+05
	P-value	-	7.2208E-06	3.0199E-11	4.6159E-10	3.0199E-11	3.0199E-11	3.0199E-11	3.0199E-11	3.0199E-11	3.0199E-11	3.0199E-11	3.0199E-11	3.0199E-11	3.0199E-11	3.0199E-11
	Rank	1	2	9	3	10	13	14	15	12	6	4	5	11	7	8
19	Mean	**1.9067E+03**	1.9143E+03	6.6548E+03	1.9311E+03	9.5109E+03	8.6306E+05	1.6271E+04	1.5228E+06	3.9073E+05	1.9783E+03	1.9821E+03	1.9774E+03	9.2386E+03	8.5408E+03	9.6078E+04
	std	2.3222E+00	5.2844E+00	5.3092E+03	5.6973E+00	8.8974E+03	1.2305E+06	1.5545E+04	1.0230E+05	7.0297E+05	1.2838E+01	4.1421E+01	3.6745E+01	9.9586E+03	8.1873E+03	4.7677E+05
	P-value	-	1.6947E-09	3.0199E-11	3.0199E-11	3.0199E-11	3.0199E-11	3.0199E-11	3.0199E-11	3.0199E-11	3.0199E-11	3.0199E-11	3.0199E-11	3.0199E-11	3.0199E-11	3.0199E-11
	Rank	1	2	7	3	10	14	12	15	13	5	6	4	9	8	11
20	Mean	**2.0898E+03**	2.1673E+03	2.2211E+03	2.1525E+03	2.6791E+03	2.3572E+03	2.7221E+03	2.7303E+03	2.6283E+03	2.1871E+03	2.1539E+03	2.0925E+03	2.2024E+03	2.2090E+03	2.4063E+03
	std	6.0735E+01	5.4199E+01	1.2496E+02	5.3019E+01	1.8927E+02	1.5392E+02	2.1077E+02	1.6892E+02	2.3267E+02	4.9264E+01	6.0055E+01	3.9306E+01	7.3903E+01	7.0434E+01	1.8904E+02

	P-value	-	1.5964E-07	2.3168E-06	5.8737E-04	3.0199E-11	3.1589E-10	3.0199E-11	3.0199E-11	3.0199E-11	3.3242E-06	1.7479E-05	3.8710E-01	3.0811E-08	7.1186E-09	1.3289E-10
	Rank	1	5	8	3	13	10	14	15	12	6	4	2	7	9	11
	Mean	**2.3239E+03**	2.3359E+03	2.3535E+03	2.3039E+03	2.5136E+03	2.3752E+03	2.4892E+03	2.6267E+03	2.5035E+03	2.3894E+03	2.3272E+03	2.3271E+03	2.3464E+03	2.3491E+03	2.3866E+03
21	std	7.3739E+00	1.0001E+01	1.2703E+01	8.0158E+01	4.9378E+01	1.9562E+01	3.8306E+01	5.0025E+01	3.4887E+01	1.4044E+01	4.9034E+00	4.6923E+00	1.2911E+01	1.4417E+01	3.1130E+01
	P-value	-	1.0277E-06	2.1544E-10	2.0095E-01	3.0199E-11	4.0772E-11	3.0199E-11	3.0199E-11	3.0199E-11	3.0199E-11	3.0339E-03	2.3800E-03	8.8910E-10	8.1014E-10	4.9752E-11
	Rank	1	4	8	5	14	9	12	15	13	11	2	3	6	7	10
	Mean	2.3000E+03	2.3007E+03	3.6227E+03	2.3013E+03	5.2934E+03	4.7578E+03	5.2956E+03	8.3607E+03	5.3469E+03	2.3000E+03	**2.3001E+03**	2.3003E+03	2.9049E+03	2.5310E+03	3.2762E+03
22	std	1.3852E-08	1.1872E+00	1.7687E+03	2.4406E+00	2.3886E+03	1.7397E+03	2.2481E+03	8.3914E+02	2.1169E+03	4.1967E-03	4.4792E-01	8.5842E-01	1.2388E+03	8.8423E+02	1.8349E+03
	P-value	-	3.0199E-11	1.4128E-01	3.0199E-11	3.0199E-11	3.0199E-11	3.0199E-11	3.0199E-11	3.0199E-11	3.0199E-11	7.7540E-11	1.0075E-07	3.0199E-11	6.6975E-05	6.6250E-01
	Rank	3	9	8	10	11	13	12	15	14	5	1	2	7	4	6
	Mean	2.6733E+03	2.6824E+03	2.7086E+03	2.6893E+03	2.9127E+03	2.7452E+03	2.9447E+03	3.3715E+03	2.8649E+03	2.7389E+03	**2.6736E+03**	2.6735E+03	2.6988E+03	2.7007E+03	2.7700E+03
23	std	1.0681E+01	1.2678E+01	2.0340E+01	3.2651E+01	6.9664E+01	4.6208E+01	9.4667E+01	1.1627E+02	6.0763E+01	1.8005E+01	6.6081E+00	7.4707E+00	2.0777E+01	1.7238E+01	3.5906E+01
	P-value	-	1.5638E-02	8.1014E-10	1.6238E-01	3.0199E-11	3.3384E-11	3.0199E-11	3.0199E-11	3.0199E-11	3.3384E-11	8.5338E-01	8.8830E-01	8.1975E-07	2.0152E-08	3.0199E-11
	Rank	2	4	8	5	13	9	14	15	12	10	1	3	6	7	11
	Mean	2.8484E+03	2.8568E+03	2.8795E+03	2.8824E+03	3.1210E+03	2.9093E+03	3.1451E+03	3.6939E+03	3.0167E+03	2.9035E+03	2.8448E+03	2.8431E+03	2.8674E+03	2.8747E+03	2.9600E+03
24	std	7.8981E+00	1.2991E+01	1.8528E+01	1.5982E+01	7.8723E+01	4.8506E+01	7.4340E+01	1.6549E+02	4.5331E+01	1.0209E+01	4.6303E+00	**5.2123E+00**	1.8002E+01	3.9809E+01	5.2466E+01
	P-value	-	5.8282E-03	8.1014E-10	1.6132E-10	3.0199E-11	3.0199E-11	3.0199E-11	3.0199E-11	3.0199E-11	3.0199E-11	5.5546E-02	2.8913E-03	2.0023E-06	5.8587E-06	3.0199E-11
	Rank	3	4	7	8	13	9	14	15	12	10	2	1	5	6	11
	Mean	**2.8869E+03**	2.8872E+03	2.8868E+03	2.8867E+03	2.8987E+03	2.9633E+03	2.8992E+03	4.9894E+03	2.9179E+03	2.8859E+03	2.8872E+03	2.8871E+03	2.8874E+03	2.9075E+03	2.9646E+03
25	std	1.9533E+00	1.7593E+00	3.8036E+00	1.1318E+00	2.1092E+01	3.7220E+01	1.9213E+01	6.1037E+02	2.4592E+01	1.9233E+00	5.3345E-01	4.2759E-01	3.0767E+00	1.6100E+01	2.5594E+02
	P-value	-	1.2477E-04	3.2553E-01	1.0407E-04	1.2235E-01	3.0199E-11	6.0971E-03	6.0971E-03	1.0702E-09	4.3764E-01	6.5486E-04	1.1738E-03	1.1143E-03	9.9186E-11	2.6015E-08
	Rank	1	8	4	6	9	14	10	15	13	2	5	3	7	12	11
	Mean	3.4184E+03	3.8884E+03	4.1961E+03	**2.9001E+03**	6.2703E+03	4.4862E+03	6.5021E+03	1.0006E+04	6.0212E+03	3.6359E+03	3.8019E+03	3.7313E+03	3.4901E+03	3.5628E+03	4.4388E+03
F26	std	5.1469E+02	4.3463E+02	2.1290E+02	8.0186E-02	1.1997E+03	2.6458E+02	9.4392E+02	9.8160E+02	6.6961E+02	8.2236E+02	8.9821E+01	1.7982E+02	5.8988E+02	7.0873E+02	1.0318E+03
	P-value	-	5.2650E-05	4.9980E-09	6.6273E-01	2.2273E-09	8.9934E-11	1.4643E-10	1.4643E-10	3.0199E-11	4.6756E-02	3.2651E-02	1.4128E-01	4.1191E-01	9.7052E-01	3.7690E-04
	Rank	2	8	9	1	12	11	14	15	13	7	6	4	3	5	10
	Mean	3.2034E+03	3.2061E+03	3.2164E+03	**3.2036E+03**	3.2653E+03	3.2385E+03	3.2733E+03	4.4414E+03	3.2595E+03	3.2200E+03	3.2088E+03	3.2095E+03	3.2142E+03	3.2447E+03	3.2519E+03
27	std	1.3063E+01	1.0722E+01	9.6772E+00	5.8157E+00	4.2219E+01	1.5339E+01	3.0757E+01	2.7977E+02	3.4863E+01	7.7773E+00	8.2609E+00	1.0395E+01	1.2800E+01	1.4400E+01	9.4922E+01
	P-value	-	1.1536E-01	4.9426E-05	3.3285E-01	1.3289E-10	8.8910E-10	4.5043E-11	4.5043E-11	7.3803E-10	1.6062E-06	1.3832E-02	2.0681E-02	1.1143E-03	2.1544E-10	9.2603E-09

	Rank	2	3	7	1	13	10	14	15	12	8	4	5	6	11	9
28	Mean	**3.1145E+03**	3.1348E+03	3.2086E+03	3.2063E+03	3.2130E+03	3.3688E+03	3.2319E+03	6.4318E+03	3.3392E+03	3.1954E+03	3.1614E+03	3.1557E+03	3.2070E+03	3.1681E+03	3.2678E+03
	std	3.7612E+01	4.9378E+01	1.9859E+01	9.3792E+00	1.9049E+01	4.5406E+01	2.1901E+01	7.6270E+02	7.2603E+01	1.7448E+01	5.9263E+01	5.6128E+01	1.5447E+01	7.1820E+01	1.9875E+02
	P-value	-	3.8053E-07	1.4294E-08	1.5581E-08	7.1186E-09	3.0199E-11	1.7769E-10	1.7769E-10	3.0199E-11	3.2555E-07	7.9583E-01	2.1540E-06	2.0152E-08	7.7272E-02	1.4294E-08
	Rank	1	2	7	9	10	14	12	15	13	5	4	3	8	6	11
29	Mean	**3.3380E+03**	3.3683E+03	3.5746E+03	3.4934E+03	4.1335E+03	3.7146E+03	4.0703E+03	6.0107E+03	4.1167E+03	3.6697E+03	3.4026E+03	3.4139E+03	3.4805E+03	3.5414E+03	3.8553E+03
	std	1.7957E+01	4.5331E+01	1.8630E+02	9.4907E+01	2.6302E+02	2.0106E+02	2.2573E+02	7.2036E+02	2.7766E+02	9.2398E+01	3.1303E+01	3.7917E+01	9.4385E+01	1.1108E+02	2.1524E+02
	P-value	-	3.1573E-05	1.5465E-09	8.1527E-11	3.0199E-11	3.0199E-11	3.0199E-11	3.0199E-11	3.0199E-11	3.0199E-11	2.6695E-09	1.2057E-10	4.9752E-11	3.3384E-11	3.0199E-11
	Rank	1	2	7	6	14	10	13	15	12	9	3	4	5	8	11
30	Mean	**5.0471E+03**	5.3496E+03	9.0666E+03	6.3297E+03	1.3412E+04	7.7555E+06	8.4798E+04	3.8355E+08	6.9622E+05	1.9139E+04	5.3924E+03	5.5623E+03	1.3246E+04	2.0971E+04	4.4807E+06
	std	1.1353E+02	2.5512E+02	5.0671E+03	5.7989E+02	5.3631E+03	4.4525E+06	5.1648E+04	5.7512E+08	1.0995E+06	4.3190E+03	2.8887E+02	3.4004E+02	8.1160E+03	1.4250E+04	2.4483E+07
	P-value	-	5.5329E-08	4.0772E-11	3.3384E-11	3.0199E-11	3.0199E-11	3.0199E-11	3.0199E-11	3.0199E-11	3.0199E-11	8.4848E-09	7.3803E-10	3.0199E-11	3.0199E-11	4.0772E-11
	Rank	1	2	6	5	9	14	12	15	13	11	3	4	8	10	7
MeanRank		**1.5517**	4.4138	7.6207	5.3793	11.6552	11.6897	12.0345	14.9655	12.8966	7.3448	2.8276	3.2759	7.3448	7.4828	9.5172
Rank		**1**	4	9	5	11	12	13	15	14	6	2	3	6	8	10

Table S2. Comparison of results of IAROA with other algorithms (50- dimensional CEC2017).

F	Index	IAROA	AROA	EO	MPA	SSA	GWO	AVOA	AOA	DBO	NOA	LSHADE_ SPACMA	LSHADE_ cnEpSin	SRPSO	XPSO	TAPSO
1	Mean	2.2124E+03	8.5679E+04	3.8506E+03	3.7332E+05	9.4401E+03	4.8940E+09	5.4193E+03	1.0550E+11	1.9370E+08	1.4182E+04	**3.3768E+02**	2.0276E+03	5.1916E+03	4.3777E+03	1.0136E+10
	std	2.0357E+03	2.9524E+04	5.2514E+03	3.3855E+05	5.2781E+03	2.4363E+09	7.3047E+03	1.0054E+10	1.1227E+08	6.6844E+03	4.4945E+02	2.5462E+03	4.3051E+03	5.6946E+03	2.6275E+10
	P-value	-	3.0199E-11	4.0354E-01	3.0199E-11	4.9980E-09	3.0199E-11	9.0490E-02	9.0490E-02	3.0199E-11	4.5043E-11	3.3242E-06	3.6322E-01	5.2640E-04	2.4581E-01	3.6439E-02
	Rank	2	11	4	12	9	14	6	15	13	10	1	3	7	5	8
3	Mean	**3.2658E+02**	3.9300E+02	4.5331E+04	1.1987E+03	1.5379E+05	8.9279E+04	5.8757E+04	1.6769E+05	2.1887E+05	3.4111E+04	5.0937E+04	4.9219E+03	8.1669E+04	6.1673E+04	4.6336E+04
	std	2.0369E+01	5.2011E+01	9.7930E+03	5.8981E+02	3.4733E+04	2.1260E+04	1.3310E+04	1.8170E+04	5.4447E+04	7.5633E+03	9.0847E+04	7.8170E+03	1.5735E+04	1.3017E+04	1.6669E+04
	P-value	-	1.2870E-09	3.0199E-11	3.0199E-11	3.0199E-11	3.0199E-11	3.0199E-11	3.0199E-11	3.0199E-11	3.0199E-11	3.0199E-11	3.0199E-11	3.0199E-11	3.0199E-11	3.0199E-11
	Rank	1	2	7	3	13	12	10	14	15	5	6	4	11	9	8
4	Mean	**4.6391E+02**	4.7883E+02	5.2822E+02	5.5230E+02	5.5959E+02	8.9853E+02	5.7246E+02	2.8350E+04	7.4743E+02	5.3238E+02	5.0089E+02	5.2986E+02	5.6880E+02	6.5663E+02	3.5796E+03
	std	3.7256E+01	3.9491E+01	5.7427E+01	3.8073E+01	5.1360E+01	1.5594E+02	5.0726E+01	5.0641E+03	1.2861E+02	3.9133E+01	5.6359E+01	4.8542E+01	4.1165E+01	4.9121E+01	9.9246E+03

	P-value	-	2.0095E-01	1.8682E-05	1.4110E-09	4.1825E-09	3.0199E-11	1.1737E-09	1.1737E-09	3.0199E-11	1.2541E-07	1.9527E-03	1.4918E-06	6.7220E-10	3.6897E-11	1.0666E-07
	Rank	1	2	5	7	8	14	10	15	13	6	3	4	9	12	11
5	Mean	**5.6401E+02**	6.2175E+02	6.5362E+02	6.7165E+02	8.8468E+02	6.9608E+02	8.3637E+02	1.1332E+03	9.4403E+02	7.8365E+02	5.6401E+02	5.8245E+02	6.2122E+02	6.0358E+02	7.0472E+02
	std	1.5503E+01	2.4998E+01	2.9753E+01	3.3593E+01	2.3195E+01	4.8812E+01	4.9521E+01	4.0558E+01	7.8880E+01	2.3932E+01	1.8317E+01	1.0764E+01	2.4106E+01	2.7687E+01	4.5487E+01
	P-value	-	1.6132E-10	3.6897E-11	3.0199E-11	3.0199E-11	3.0199E-11	3.0199E-11	3.0199E-11	3.0199E-11	3.0199E-11	7.2827E-01	4.4205E-06	2.6099E-10	1.3594E-07	3.0199E-11
	Rank	1	6	7	8	13	9	12	15	14	11	2	3	5	4	10
6	Mean	**6.0010E+02**	6.0042E+02	6.0037E+02	6.0788E+02	6.6247E+02	6.1198E+02	6.5267E+02	6.9039E+02	6.4641E+02	6.0047E+02	6.0016E+02	6.0037E+02	6.0058E+02	6.0077E+02	6.1012E+02
	std	2.4298E-02	2.0733E-01	2.8967E-01	1.7290E+00	8.6328E+00	3.2024E+00	6.9174E+00	5.2891E+00	9.7707E+00	1.7124E-01	1.4771E-01	2.0890E-01	5.9952E-01	4.3992E-01	5.0777E+00
	P-value	-	3.0199E-11	3.0103E-07	3.0199E-11	3.0199E-11	3.0199E-11	3.0199E-11	3.0199E-11	3.0199E-11	3.0199E-11	3.6322E-01	7.3803E-10	1.3111E-08	3.0199E-11	3.0199E-11
	Rank	1	6	3	9	14	11	13	15	12	7	2	4	5	8	10
7	Mean	8.0765E+02	8.5148E+02	9.0850E+02	9.1778E+02	1.7598E+03	1.0383E+03	1.5315E+03	1.9188E+03	1.2166E+03	1.0598E+03	**8.0152E+02**	8.4175E+02	9.4216E+02	8.4914E+02	1.0134E+03
	std	1.1349E+01	2.0641E+01	4.8294E+01	3.7455E+01	6.8035E+01	7.4655E+01	1.1161E+02	5.6339E+01	1.3708E+02	1.5463E+01	2.1452E+01	1.6611E+01	8.7549E+01	2.4991E+01	2.2190E+02
	P-value	-	1.4643E-10	3.0199E-11	3.0199E-11	3.0199E-11	3.0199E-11	3.0199E-11	3.0199E-11	3.0199E-11	3.0199E-11	5.7460E-02	3.1589E-10	3.6897E-11	3.8249E-09	3.0199E-11
	Rank	2	5	6	7	14	10	13	15	12	11	1	3	8	4	9
8	Mean	**8.6613E+02**	9.2095E+02	9.5069E+02	9.7444E+02	1.2007E+03	1.0068E+03	1.1415E+03	1.4576E+03	1.2233E+03	1.0838E+03	8.6483E+02	8.8408E+02	9.0395E+02	8.9724E+02	9.9722E+02
	std	1.4239E+01	2.6004E+01	3.7334E+01	3.7725E+01	3.2757E+01	5.9917E+01	5.6784E+01	4.2631E+01	6.4997E+01	3.0888E+01	1.9634E+01	1.3803E+01	2.0422E+01	2.2995E+01	4.2115E+01
	P-value	-	7.3803E-10	7.3891E-11	3.3384E-11	3.0199E-11	3.0199E-11	3.0199E-11	3.0199E-11	3.0199E-11	3.0199E-11	7.5059E-01	3.5708E-06	3.1967E-09	3.8053E-07	3.0199E-11
	Rank	1	6	7	8	13	10	12	15	14	11	2	3	5	4	9
9	Mean	**9.0267E+02**	9.8321E+02	1.1695E+03	1.7692E+03	1.3282E+04	4.9983E+03	1.3138E+04	2.6036E+04	1.4941E+04	1.0476E+03	9.1711E+02	9.5598E+02	1.2270E+03	9.6213E+02	5.8725E+03
	std	3.8158E+00	1.0076E+02	4.5960E+02	3.6305E+02	7.1976E+02	2.2066E+03	1.6402E+03	2.9887E+03	4.6047E+03	1.2200E+02	1.1449E+01	5.7433E+01	1.1922E+03	4.6259E+01	2.0636E+03
	P-value	-	4.9752E-11	5.4941E-11	3.0199E-11	3.0199E-11	3.0199E-11	3.0199E-11	3.0199E-11	3.0199E-11	3.3384E-11	2.9215E-09	4.9752E-11	1.4643E-10	4.5043E-11	3.0199E-11
	Rank	1	6	7	9	13	10	12	15	14	8	2	4	3	5	11
10	Mean	**5.0824E+03**	6.1059E+03	7.5063E+03	6.0589E+03	8.3859E+03	7.6729E+03	8.0089E+03	1.2989E+04	8.9209E+03	1.1047E+04	6.7355E+03	6.8163E+03	7.4015E+03	6.4504E+03	7.0521E+03
	std	7.4657E+02	6.9053E+02	9.1159E+02	5.7565E+02	9.4846E+02	2.2991E+03	1.2877E+03	7.3463E+02	1.2840E+03	4.5703E+02	3.3436E+02	6.5637E+02	2.0526E+03	7.9640E+02	1.0117E+03
	P-value	-	5.4620E-06	1.3289E-10	3.3242E-06	3.0199E-11	3.4971E-09	3.1589E-10	3.1589E-10	3.0199E-11	3.0199E-11	9.9186E-11	4.1997E-10	3.3520E-08	1.8731E-07	5.4617E-09
	Rank	1	3	10	2	12	9	11	15	13	14	5	6	8	4	7
11	Mean	**1.1521E+03**	1.1902E+03	1.2529E+03	1.2134E+03	1.3532E+03	3.8585E+03	1.3435E+03	2.1632E+04	2.0263E+03	1.3286E+03	1.2757E+03	1.2771E+03	1.2578E+03	1.3058E+03	2.1494E+03
	std	1.2140E+01	2.0829E+01	6.0655E+01	2.0068E+01	6.1989E+01	1.4171E+03	5.2375E+01	2.8906E+03	2.0985E+02	2.8286E+01	3.8130E+01	4.7209E+01	3.4875E+01	5.1692E+01	2.4033E+03
	P-value	-	1.5465E-09	1.0937E-10	4.9752E-11	3.0199E-11	3.0199E-11	3.0199E-11	3.0199E-11	3.0199E-11	3.0199E-11	3.0199E-11	3.0199E-11	3.3384E-11	3.0199E-11	3.0199E-11

	Rank	1	2	4	3	12	14	11	15	13	10	6	7	5	8	9
12	Mean	**1.1400E+05**	4.9300E+05	1.7292E+06	3.7366E+06	4.9536E+06	4.3956E+08	1.6296E+07	6.3618E+10	1.7566E+08	1.4667E+06	1.6830E+05	2.2902E+05	3.1174E+06	1.6448E+06	3.6467E+09
	std	7.6608E+04	3.1360E+05	1.2586E+06	2.1367E+06	2.7863E+06	4.5917E+08	1.3560E+07	1.1966E+10	1.5944E+08	6.4769E+05	1.1467E+05	2.4635E+05	1.6284E+06	3.1791E+06	1.1944E+10
	P-value	-	5.4617E-09	3.3384E-11	3.0199E-11	3.0199E-11	3.0199E-11	3.0199E-11	3.0199E-11	3.0199E-11	3.0199E-11	3.9167E-02	1.4423E-03	3.0199E-11	1.9568E-10	5.4941E-11
	Rank	1	4	7	10	11	14	12	15	13	6	2	3	9	5	8
13	Mean	4.0489E+03	**3.3197E+03**	9.0873E+03	9.9784E+03	1.5874E+04	1.1046E+08	9.4124E+04	2.4742E+10	6.1761E+06	1.7596E+04	5.2989E+03	5.9209E+03	8.0534E+03	9.6348E+03	6.4094E+06
	std	4.2105E+03	3.1031E+03	7.3751E+03	1.4566E+03	1.1697E+04	1.7021E+08	4.3592E+04	7.1311E+09	1.0038E+07	6.8604E+03	1.4588E+03	1.5412E+03	7.2347E+03	6.3823E+03	3.5035E+07
	P-value	-	4.6427E-01	2.9590E-05	2.6015E-08	7.6950E-08	3.0199E-11	3.0199E-11	3.0199E-11	3.0199E-11	6.7220E-10	1.1747E-04	1.2493E-05	3.3386E-03	5.8587E-06	3.5708E-06
	Rank	2	1	6	9	10	14	12	15	13	11	3	4	5	7	8
14	Mean	**1.4449E+03**	1.4869E+03	9.0677E+04	1.5149E+03	1.5711E+05	6.6143E+05	3.2819E+05	2.7290E+07	1.3164E+06	1.7911E+03	1.6685E+03	1.6481E+03	8.1073E+04	6.0267E+04	1.6844E+05
	std	8.1149E+00	4.7972E+01	6.5888E+04	1.9335E+01	9.3192E+04	6.4353E+05	1.6587E+05	2.7212E+07	1.7972E+06	6.6469E+01	7.0980E+01	4.4503E+01	5.9595E+04	4.6129E+04	4.9249E+05
	P-value	-	6.1210E-10	3.0199E-11	3.0199E-11	3.0199E-11	3.0199E-11	3.0199E-11	3.0199E-11	3.0199E-11	3.0199E-11	3.0199E-11	3.0199E-11	3.0199E-11	3.0199E-11	3.0199E-11
	Rank	1	2	10	3	11	13	12	15	14	6	5	4	9	7	8
15	Mean	4.3753E+03	9.2054E+03	1.3680E+04	2.0513E+03	1.5304E+04	1.5653E+07	4.6342E+04	1.1657E+09	7.7659E+06	3.5499E+03	**1.9952E+03**	2.0938E+03	8.1484E+03	6.6840E+03	1.0135E+04
	std	4.1217E+03	4.2379E+03	6.1929E+03	6.5874E+01	7.1870E+03	2.5959E+07	2.3344E+04	1.5394E+09	2.6812E+07	4.5017E+02	1.3393E+02	1.8654E+02	6.9253E+03	6.4292E+03	7.6677E+03
	P-value	-	7.7387E-06	1.5964E-07	9.0688E-03	1.6980E-08	3.0199E-11	4.5043E-11	4.5043E-11	3.0199E-11	5.7460E-02	1.2362E-03	9.8834E-03	6.0971E-03	7.0127E-02	5.5611E-04
	Rank	4	9	10	3	11	13	12	15	14	5	1	2	7	6	8
16	Mean	2.5424E+03	2.6228E+03	2.9263E+03	2.5956E+03	4.0134E+03	2.9696E+03	3.8123E+03	6.7094E+03	4.2960E+03	2.9166E+03	2.6308E+03	**2.4076E+03**	2.4480E+03	2.7232E+03	3.1304E+03
	std	3.0561E+02	3.1440E+02	3.7869E+02	2.1847E+02	5.1274E+02	3.8025E+02	5.4599E+02	1.4344E+03	6.4991E+02	2.7566E+02	3.1826E+02	1.9814E+02	2.9130E+02	3.3079E+02	4.0817E+02
	P-value	-	3.4783E-01	1.7836E-04	2.9047E-01	4.9752E-11	2.4327E-05	1.9568E-10	1.9568E-10	4.0772E-11	1.4298E-05	1.1536E-01	6.3533E-02	2.0095E-01	3.3874E-02	4.4440E-07
	Rank	3	5	8	4	13	10	12	15	14	9	6	1	2	7	11
17	Mean	**2.3660E+03**	2.5411E+03	2.7461E+03	2.5639E+03	3.4329E+03	2.7713E+03	3.6318E+03	6.1407E+03	3.8990E+03	2.7580E+03	2.5880E+03	2.4009E+03	2.5604E+03	2.7469E+03	3.1224E+03
	std	1.4404E+02	2.1070E+02	3.0161E+02	1.8213E+02	3.6611E+02	3.2504E+02	3.5454E+02	1.6442E+03	3.8969E+02	1.3736E+02	2.0161E+02	1.7531E+02	2.7521E+02	2.5220E+02	3.5983E+02
	P-value	-	1.2362E-03	7.0430E-07	1.9963E-05	3.0199E-11	1.0277E-06	3.0199E-11	3.0199E-11	3.0199E-11	1.6132E-10	4.9426E-05	5.8945E-01	5.0842E-03	6.0104E-08	9.9186E-11
	Rank	1	5	7	4	12	8	13	15	14	10	6	2	3	9	11
18	Mean	2.5405E+03	4.4354E+03	1.2459E+06	**2.2857E+03**	1.5621E+06	5.0183E+06	2.1497E+06	7.2992E+07	5.8948E+06	2.9910E+04	9.3637E+03	7.6080E+03	1.5905E+06	6.3784E+05	1.5265E+06
	std	6.6279E+02	1.6863E+03	7.2673E+05	1.3050E+02	1.2661E+06	9.2520E+06	1.4780E+06	5.0592E+07	8.7637E+06	8.1903E+03	6.8198E+03	7.8103E+03	1.2077E+06	1.1055E+06	5.9606E+06
	P-value	-	7.0881E-08	3.0199E-11	4.2896E-01	3.0199E-11	3.0199E-11	3.0199E-11	3.0199E-11	3.0199E-11	3.0199E-11	9.9186E-11	7.1186E-09	3.0199E-11	3.0199E-11	3.0199E-11
	Rank	2	3	9	1	10	14	12	15	13	6	5	4	11	8	7

19	Mean	**2.0274E+03**	1.3649E+04	1.9990E+04	2.0264E+03	1.9193E+04	2.8401E+06	3.2912E+04	8.7138E+08	3.7282E+06	2.8712E+03	2.0929E+03	2.1012E+03	1.5606E+04	1.3694E+04	1.9558E+04
	std	2.7482E+02	4.9565E+03	1.0955E+04	2.2151E+01	1.4101E+04	5.9069E+06	1.4506E+04	8.5261E+08	5.4324E+06	4.0918E+02	6.0937E+01	7.1031E+01	1.0791E+04	9.6425E+03	2.7579E+04
	P-value	-	3.0199E-11	3.3384E-11	1.5292E-05	4.9752E-11	3.0199E-11	3.0199E-11	3.0199E-11	3.0199E-11	5.0723E-10	9.5332E-07	9.5332E-07	9.9186E-11	6.0658E-11	6.0658E-11
	Rank	1	8	11	2	10	14	12	15	13	5	3	4	7	6	9
20	Mean	**2.3442E+03**	2.5461E+03	2.7942E+03	2.6116E+03	3.6196E+03	2.8771E+03	3.5458E+03	3.5108E+03	3.5535E+03	2.7089E+03	2.7898E+03	2.4990E+03	2.5792E+03	2.7070E+03	2.9050E+03
	std	1.7944E+02	2.0668E+02	3.0081E+02	1.7324E+02	2.9858E+02	3.4185E+02	2.8000E+02	3.0949E+02	2.9064E+02	1.4220E+02	2.4141E+02	1.3184E+02	2.7551E+02	2.5622E+02	2.7904E+02
	P-value	-	3.5638E-04	1.2541E-07	1.8608E-06	3.0199E-11	1.6947E-09	3.0199E-11	3.0199E-11	3.0199E-11	5.9673E-09	2.1947E-08	8.5641E-04	4.2175E-04	6.5261E-07	7.3803E-10
	Rank	1	3	9	5	15	10	13	12	14	7	8	2	4	6	11
21	Mean	**2.3647E+03**	2.3904E+03	2.4206E+03	2.4374E+03	2.7498E+03	2.4925E+03	2.7068E+03	3.0780E+03	2.7337E+03	2.5447E+03	2.3862E+03	2.3925E+03	2.4073E+03	2.3943E+03	2.4869E+03
	std	1.3217E+01	1.8657E+01	2.9776E+01	2.6797E+01	1.0466E+02	5.2066E+01	7.7501E+01	6.8826E+01	6.2300E+01	1.7521E+01	1.6016E+01	1.1511E+01	2.7399E+01	1.9341E+01	7.2533E+01
	P-value	-	8.1975E-07	6.1210E-10	4.9752E-11	3.0199E-11	3.0199E-11	3.0199E-11	3.0199E-11	3.0199E-11	3.0199E-11	1.1937E-06	1.0105E-08	1.2023E-08	4.6856E-08	3.3384E-11
	Rank	1	3	7	8	14	10	12	15	13	11	2	4	6	5	9
22	Mean	**2.7188E+03**	6.0285E+03	8.7077E+03	2.5342E+03	1.0236E+04	9.0444E+03	1.0005E+04	1.5606E+04	1.0419E+04	9.3370E+03	6.4105E+03	7.5925E+03	7.2663E+03	6.8932E+03	9.1500E+03
	std	1.2792E+03	2.7348E+03	1.7743E+03	1.2464E+03	1.0535E+03	1.5564E+03	7.8029E+02	5.8473E+02	1.0834E+03	4.8806E+03	2.9896E+03	2.2334E+03	2.7629E+03	3.1935E+03	1.0041E+03
	P-value	-	1.2870E-09	6.1210E-10	8.3520E-08	3.0199E-11	3.0199E-11	3.0199E-11	3.0199E-11	3.0199E-11	7.3803E-10	1.1737E-09	2.0338E-09	3.8202E-10	1.2477E-04	3.0199E-11
	Rank	1	3	8	2	13	9	12	15	14	11	5	7	6	4	10
23	Mean	**2.7786E+03**	2.8204E+03	2.8513E+03	2.8526E+03	3.3406E+03	2.9581E+03	3.3470E+03	4.2100E+03	3.2690E+03	2.9986E+03	2.8208E+03	2.8137E+03	2.8382E+03	2.8569E+03	3.0244E+03
	std	1.5763E+01	2.5709E+01	3.4207E+01	2.3867E+01	1.0362E+02	6.0163E+01	1.0606E+02	1.7993E+02	9.6441E+01	2.3854E+01	1.3679E+01	1.5570E+01	3.4446E+01	3.7307E+01	8.2971E+01
	P-value	-	5.5329E-08	4.5043E-11	3.0199E-11	3.0199E-11	3.0199E-11	3.0199E-11	3.0199E-11	3.0199E-11	3.0199E-11	9.9186E-11	8.1014E-10	2.3715E-10	2.1544E-10	3.0199E-11
	Rank	1	4	6	8	14	9	13	15	12	10	3	2	5	7	11
24	Mean	**2.9582E+03**	2.9910E+03	3.0118E+03	3.0212E+03	3.5045E+03	3.1479E+03	3.5264E+03	4.7012E+03	3.4057E+03	3.1506E+03	2.9818E+03	2.9820E+03	3.0088E+03	3.0417E+03	3.2662E+03
	std	1.3632E+01	2.1077E+01	2.7045E+01	2.8151E+01	1.0959E+02	1.1164E+02	1.4882E+02	2.0844E+02	1.0650E+02	2.3620E+01	1.4971E+01	1.4585E+01	2.8036E+01	5.9683E+01	2.0989E+02
	P-value	-	3.9648E-08	3.4742E-10	7.3891E-11	3.0199E-11	3.0199E-11	3.0199E-11	3.0199E-11	3.0199E-11	3.0199E-11	8.8411E-07	4.8011E-07	1.6947E-09	1.6947E-09	3.0199E-11
	Rank	1	4	5	7	13	9	14	15	12	10	3	2	6	8	11
25	Mean	3.0292E+03	3.0636E+03	3.0820E+03	3.0366E+03	3.0788E+03	3.3997E+03	3.0936E+03	1.4859E+04	3.2655E+03	3.0889E+03	3.0184E+03	**3.0196E+03**	3.0445E+03	3.1432E+03	3.5772E+03
	std	3.8583E+01	2.6898E+01	2.6765E+01	2.4178E+01	3.5472E+01	1.7995E+02	2.4222E+01	1.7489E+03	4.9426E+02	2.2436E+01	4.0204E+01	3.0737E+01	2.5754E+01	3.8810E+01	1.7175E+03
	P-value	-	4.0840E-05	7.0881E-08	5.5923E-01	5.8587E-06	3.0199E-11	3.1967E-09	3.1967E-09	4.1997E-10	4.9980E-09	5.6922E-01	1.9579E-01	6.1452E-02	3.0199E-11	3.7704E-04
	Rank	3	6	9	4	8	14	11	15	13	10	2	1	5	12	7
26	Mean	3.7110E+03	4.8374E+03	5.1311E+03	3.4219E+03	9.2811E+03	6.0484E+03	9.3685E+03	1.6386E+04	9.3332E+03	6.0479E+03	4.4988E+03	4.5130E+03	4.5371E+03	**3.5946E+03**	6.4075E+03

	std	7.8103E+02	8.2825E+02	4.0858E+02	7.8075E+02	2.9194E+03	5.9083E+02	2.4808E+03	8.5925E+02	1.0087E+03	1.1792E+03	1.5608E+02	1.7476E+02	7.1012E+02	8.7550E+02	2.2299E+03
	P-value	-	2.6015E-08	8.1527E-11	4.8252E-01	4.5726E-09	3.0199E-11	2.9215E-09	2.9215E-09	3.0199E-11	2.9215E-09	8.1465E-05	4.3531E-05	6.5261E-07	5.0120E-02	1.1077E-06
	Rank	2	7	8	3	12	11	13	15	14	10	5	4	6	1	9
27	Mean	3.2957E+03	3.3236E+03	3.3267E+03	3.3365E+03	3.6866E+03	3.5138E+03	3.7430E+03	6.5293E+03	3.6312E+03	3.4810E+03	3.3093E+03	3.3102E+03	3.3259E+03	3.5290E+03	3.5566E+03
	std	5.0341E+01	6.1999E+01	4.0931E+01	4.6535E+01	1.7129E+02	6.8300E+01	1.7037E+02	5.2380E+02	9.4779E+01	5.6782E+01	5.0437E+01	4.1401E+01	5.5845E+01	9.1812E+01	3.4421E+02
	P-value	-	6.7869E-02	1.1143E-03	1.5846E-04	3.3384E-11	9.9186E-11	3.3384E-11	3.3384E-11	3.3384E-11	1.7769E-10	2.3985E-01	8.7710E-02	2.0681E-02	7.3891E-11	1.1737E-09
	Rank	1	4	6	7	12	10	14	15	13	9	3	2	5	11	8
28	Mean	3.2872E+03	3.3101E+03	3.3157E+03	3.3037E+03	3.3351E+03	3.8883E+03	3.3430E+03	1.1800E+04	4.9025E+03	3.3416E+03	3.2921E+03	3.2976E+03	3.2960E+03	3.4151E+03	3.8629E+03
	std	2.2368E+01	2.3294E+01	3.2613E+01	2.7963E+01	2.2738E+01	1.7809E+02	3.1701E+01	1.1301E+03	1.8934E+03	3.0614E+01	2.1771E+01	1.8738E+01	2.4458E+01	6.3007E+01	1.1470E+03
	P-value	-	6.2828E-06	1.9963E-05	3.3874E-02	2.3715E-10	3.0199E-11	1.4643E-10	1.4643E-10	3.0199E-11	1.8500E-08	9.2344E-01	1.2967E-01	5.2640E-04	8.9934E-11	6.7650E-05
	Rank	1	6	7	5	9	14	11	15	13	10	2	3	4	12	8
29	Mean	3.4915E+03	3.5819E+03	3.7589E+03	4.0440E+03	5.1910E+03	4.3791E+03	4.9909E+03	2.1824E+04	5.4293E+03	4.1952E+03	3.5096E+03	3.5578E+03	3.8367E+03	4.0716E+03	4.2582E+03
	std	1.5408E+02	1.9964E+02	3.0472E+02	2.2333E+02	4.5376E+02	2.7615E+02	4.2107E+02	7.6443E+03	6.4197E+02	1.7059E+02	1.1492E+02	1.4804E+02	2.1188E+02	2.6873E+02	4.7909E+02
	P-value	-	1.5798E-01	2.6806E-04	1.4643E-10	3.0199E-11	3.0199E-11	3.0199E-11	3.0199E-11	3.0199E-11	3.3384E-11	8.7663E-01	1.1882E-01	6.5277E-08	6.1210E-10	3.8202E-10
	Rank	1	3	5	7	13	11	12	15	14	10	2	4	6	8	9
30	Mean	6.4927E+05	7.7803E+05	1.1223E+06	2.1737E+06	1.1932E+06	1.0153E+08	4.1268E+06	2.8580E+09	2.3349E+07	6.1480E+06	9.5302E+05	9.8145E+05	8.8498E+05	4.5335E+06	1.4202E+07
	std	6.3270E+04	6.8184E+04	3.2569E+05	8.4225E+05	3.5593E+05	3.7218E+07	1.2274E+06	2.4899E+09	1.6524E+07	8.9253E+05	1.9534E+05	3.1816E+05	1.5387E+05	1.7234E+06	4.9424E+07
	P-value	-	8.3520E-08	9.9186E-11	3.0199E-11	4.5043E-11	3.0199E-11	3.0199E-11	3.0199E-11	3.0199E-11	3.0199E-11	4.1825E-09	5.0922E-08	1.4294E-08	3.0199E-11	5.4941E-11
	Rank	1	2	6	9	7	14	10	15	13	12	5	4	3	11	8
MeanRank		1.4138	4.5172	7.0345	5.8276	11.6897	11.5172	11.7931	14.8621	13.3103	9.0000	3.4828	3.4483	6.0345	7.0000	9.0690
Rank		1	4	8	5	12	11	13	15	14	9	3	2	6	7	10

Table S3. Comparison of results of IAROA with other algorithms (100- dimensional CEC2017).

F	Index	IAROA	AROA	EO	MPA	SSA	GWO	AVOA	AOA	DBO	NOA	LSHADE_ SPACMA	LSHADE_ cnEpSin	SRPSO	XPSO	TAPSO
1	Mean	4.7934E+05	1.2469E+07	1.7393E+06	6.7142E+07	5.0690E+06	3.2152E+10	9.6221E+05	2.6694E+11	1.8227E+10	2.9054E+07	4.1081E+04	1.1321E+05	9.9769E+06	2.8107E+04	4.4383E+10
	std	2.1319E+05	1.8176E+06	2.4044E+06	2.7941E+07	2.1147E+06	9.2415E+09	1.6564E+06	1.2968E+10	1.8449E+10	1.2398E+07	2.4488E+04	9.0082E+04	4.9340E+06	3.4586E+04	8.2108E+10
	P-value	-	3.0199E-11	5.9706E-05	3.0199E-11	3.0199E-11	3.0199E-11	1.8575E-03	1.8575E-03	3.0199E-11	3.0199E-11	3.0199E-11	2.1544E-10	3.0199E-11	3.0199E-11	1.9112E-02
	Rank	5	9	6	12	7	14	4	15	13	11	2	3	8	1	10
3	Mean	3.0465E+04	4.3900E+04	2.7121E+05	6.4269E+04	4.2631E+05	2.7394E+05	2.5846E+05	3.3441E+05	5.5516E+05	1.8825E+05	2.0948E+05	1.2490E+05	3.2646E+05	3.1468E+05	3.5007E+05

	std	5.3637E+03	7.4092E+03	3.0819E+04	1.3138E+04	5.8500E+04	2.4496E+04	2.1538E+04	1.9387E+04	2.0888E+05	1.7075E+04	1.9607E+05	6.1544E+04	3.2363E+04	4.2300E+04	1.0892E+05
	P-value	-	4.1825E-09	3.0199E-11	3.3384E-11	3.0199E-11	3.0199E-11	3.0199E-11	3.0199E-11	3.0199E-11	3.0199E-11	3.0199E-11	3.0199E-11	3.0199E-11	3.0199E-11	3.0199E-11
	Rank	1	2	8	3	14	9	7	13	15	5	6	4	12	10	11
4	Mean	**6.9028E+02**	7.3498E+02	7.8172E+02	8.1586E+02	8.2574E+02	2.9658E+03	8.4610E+02	8.5570E+04	2.0165E+03	8.4722E+02	6.9743E+02	7.1250E+02	7.4013E+02	1.1543E+03	3.2892E+03
	std	4.5625E+01	3.8161E+01	5.4325E+01	5.0846E+01	6.1086E+01	5.5097E+02	6.3636E+01	1.6332E+04	8.5015E+02	3.6310E+01	4.9146E+01	4.3930E+01	4.2531E+01	1.2353E+02	4.6495E+03
	P-value	-	3.0059E-04	6.0104E-08	2.1544E-10	6.1210E-10	3.0199E-11	7.3891E-11	7.3891E-11	3.0199E-11	3.3384E-11	5.9969E-01	1.0233E-01	1.7836E-04	3.0199E-11	2.9590E-05
	Rank	1	4	6	7	8	14	9	15	13	10	2	3	5	12	11
5	Mean	**7.2010E+02**	8.5760E+02	9.7718E+02	9.8682E+02	1.3650E+03	1.0991E+03	1.3057E+03	2.0329E+03	1.6214E+03	1.3807E+03	8.2024E+02	8.6708E+02	8.6883E+02	7.8094E+02	1.1189E+03
	std	3.7353E+01	6.2780E+01	7.3026E+01	6.9892E+01	3.0266E+01	7.1593E+01	6.8986E+01	5.5912E+01	1.5359E+02	7.3776E+01	9.4202E+01	4.1550E+01	7.5192E+01	7.0464E+01	2.1819E+02
	P-value	-	4.5043E-11	3.0199E-11	3.0199E-11	3.0199E-11	3.0199E-11	3.0199E-11	3.0199E-11	3.0199E-11	3.0199E-11	2.2539E-04	3.0199E-11	8.8910E-10	2.2539E-04	3.0199E-11
	Rank	1	4	7	8	12	10	11	15	14	13	3	6	5	2	9
6	Mean	**6.0207E+02**	6.1075E+02	6.1031E+02	6.2161E+02	6.6458E+02	6.3159E+02	6.5881E+02	7.0573E+02	6.7010E+02	6.1172E+02	6.0407E+02	6.0662E+02	6.0530E+02	6.0982E+02	6.3669E+02
	std	7.9642E-01	2.7618E+00	5.9695E+00	3.7656E+00	1.7309E+00	4.8381E+00	2.9419E+00	5.1618E+00	9.2813E+00	2.6295E+00	1.2398E+00	1.0886E+00	2.2991E+00	3.3044E+00	1.1627E+01
	P-value	-	3.0199E-11	3.3384E-11	3.0199E-11	3.0199E-11	3.0199E-11	3.0199E-11	3.0199E-11	3.0199E-11	3.0199E-11	1.4294E-08	3.3384E-11	1.2870E-09	3.3384E-11	3.0199E-11
	Rank	1	7	5	9	13	10	12	15	14	8	2	4	3	6	11
7	Mean	**9.7854E+02**	1.1695E+03	1.4205E+03	1.4384E+03	3.2327E+03	1.8568E+03	3.0039E+03	3.8703E+03	2.3097E+03	1.7389E+03	1.1206E+03	1.2812E+03	1.5201E+03	1.1243E+03	2.1265E+03
	std	2.3299E+01	4.4410E+01	1.1952E+02	9.5523E+01	1.1999E+02	1.3769E+02	1.5101E+02	8.7072E+01	4.7995E+02	4.3610E+01	1.0903E+02	5.4921E+01	1.7924E+02	7.9382E+01	1.2726E+03
	P-value	-	3.0199E-11	3.0199E-11	3.0199E-11	3.0199E-11	3.0199E-11	3.0199E-11	3.0199E-11	3.0199E-11	3.0199E-11	6.7220E-10	3.0199E-11	3.0199E-11	2.8716E-10	3.0199E-11
	Rank	1	4	6	7	14	11	13	15	12	10	3	5	8	2	9
8	Mean	**1.0312E+03**	1.1342E+03	1.2428E+03	1.2418E+03	1.8318E+03	1.4184E+03	1.7311E+03	2.4682E+03	1.9878E+03	1.6467E+03	1.1234E+03	1.1819E+03	1.1633E+03	1.0902E+03	1.4098E+03
	std	4.3441E+01	5.7656E+01	6.6001E+01	5.9726E+01	5.0456E+01	1.0207E+02	8.0538E+01	8.9441E+01	1.6598E+02	5.0367E+01	8.5284E+01	2.9743E+01	5.0515E+01	6.9469E+01	1.6682E+02
	P-value	-	5.9673E-09	3.0199E-11	3.0199E-11	3.0199E-11	3.0199E-11	3.0199E-11	3.0199E-11	3.0199E-11	3.0199E-11	4.9426E-05	3.0199E-11	1.9568E-10	6.2027E-04	3.0199E-11
	Rank	1	3	7	8	13	10	12	15	14	11	4	6	5	2	9
9	Mean	**1.0325E+03**	7.6885E+03	1.8422E+04	1.4723E+04	2.3918E+04	2.6652E+04	2.4506E+04	6.3717E+04	4.9846E+04	1.4497E+04	2.5765E+03	3.7216E+03	1.5388E+04	2.6264E+03	2.1527E+04
	std	9.2496E+01	3.2225E+03	5.6233E+03	4.2441E+03	4.3198E+02	1.0064E+04	2.2728E+03	6.3930E+03	1.9677E+04	6.8685E+03	9.7603E+02	9.1705E+02	1.2313E+04	1.3294E+03	1.1986E+04
	P-value	-	3.0199E-11	3.0199E-11	3.0199E-11	3.0199E-11	3.0199E-11	3.0199E-11	3.0199E-11	3.0199E-11	3.0199E-11	3.3384E-11	3.0199E-11	3.0199E-11	3.3384E-11	3.0199E-11
	Rank	1	5	9	7	12	11	13	15	14	8	3	4	6	2	10
10	Mean	**1.2193E+04**	1.4473E+04	1.7005E+04	1.3975E+04	1.7296E+04	1.7912E+04	1.7190E+04	2.9574E+04	1.7813E+04	2.6368E+04	1.8951E+04	1.8788E+04	1.9564E+04	1.4321E+04	1.5114E+04
	std	8.1252E+02	1.3777E+03	2.4827E+03	8.6830E+02	1.3932E+03	5.6702E+03	1.8360E+03	1.3621E+03	1.9979E+03	5.7742E+02	1.0211E+03	1.5591E+03	4.8807E+03	1.2852E+03	2.0018E+03

	P-value	-	1.5581E-08	9.9186E-11	1.8500E-08	3.0199E-11	5.4941E-11	4.0772E-11	4.0772E-11	3.6897E-11	3.0199E-11	3.0199E-11	3.0199E-11	2.3715E-10	8.4848E-09	2.4386E-09
	Rank	1	4	7	2	9	6	8	15	10	14	13	12	11	3	5
11	Mean	**1.5873E+03**	1.8434E+03	8.2250E+03	2.7293E+03	1.6243E+04	4.7583E+04	1.0516E+04	1.7551E+05	1.0461E+05	1.1855E+04	1.2454E+04	3.3202E+03	1.0064E+04	5.0913E+03	1.2703E+04
	std	4.9251E+01	1.0946E+02	2.0524E+03	2.7119E+02	5.2569E+03	1.2817E+04	4.4588E+03	2.4744E+04	3.8956E+04	1.4821E+03	2.5614E+04	3.0827E+03	2.3449E+03	2.1836E+03	3.1570E+04
	P-value	-	1.2057E-10	3.0199E-11	3.0199E-11	3.0199E-11	3.0199E-11	3.0199E-11	3.0199E-11	3.0199E-11	3.0199E-11	3.0199E-11	3.0199E-11	3.0199E-11	3.0199E-11	3.0199E-11
	Rank	1	2	8	3	12	13	9	15	14	11	5	4	10	7	6
12	Mean	6.8233E+06	3.3505E+07	2.5294E+07	9.0147E+07	6.1269E+07	5.1367E+09	1.0528E+08	1.7597E+11	1.7230E+09	3.2781E+07	**4.9998E+06**	1.0009E+07	3.4358E+07	1.1084E+08	8.9609E+09
	std	2.2428E+06	1.2044E+07	1.2063E+07	5.2184E+07	2.0998E+07	2.7365E+09	5.0915E+07	2.0988E+10	8.8759E+08	1.2655E+07	3.2253E+06	6.2502E+06	1.5218E+07	8.6624E+07	2.3974E+10
	P-value	-	3.6897E-11	6.1210E-10	3.0199E-11	3.0199E-11	3.0199E-11	3.0199E-11	3.0199E-11	3.0199E-11	3.0199E-11	9.0688E-03	7.7272E-02	3.0199E-11	1.2023E-08	9.3341E-02
	Rank	2	6	4	11	9	14	12	15	13	7	1	3	8	10	5
13	Mean	6.3293E+03	7.5314E+03	1.7463E+04	6.8682E+04	2.0784E+04	4.6713E+08	6.2867E+04	4.1193E+10	8.9245E+07	**5.9949E+03**	1.0038E+04	1.3812E+04	6.9292E+03	1.4926E+04	1.0205E+09
	std	4.1186E+03	2.5036E+03	1.6898E+04	1.4613E+05	8.4670E+03	4.5836E+08	3.0487E+04	7.4573E+09	9.4244E+07	1.9020E+03	3.3759E+03	4.0150E+03	5.2854E+03	5.8426E+03	4.1836E+09
	P-value	-	4.8413E-02	6.2828E-06	3.0199E-11	5.5727E-10	3.0199E-11	3.0199E-11	3.0199E-11	3.0199E-11	5.0114E-01	3.0059E-04	2.0283E-07	7.8446E-01	1.4733E-07	1.0907E-05
	Rank	2	4	6	11	10	14	12	15	13	1	5	7	3	9	8
14	Mean	8.5569E+03	4.0519E+04	1.0236E+06	**2.2199E+03**	1.0338E+06	4.9624E+06	1.2871E+06	6.8377E+07	7.9957E+06	4.5827E+04	2.1904E+04	1.1235E+04	1.5988E+06	5.8234E+05	7.7146E+05
	std	6.5664E+03	3.8944E+04	3.2113E+05	2.7172E+02	3.7972E+05	3.9334E+06	5.2216E+05	2.5559E+07	7.9514E+06	1.5744E+04	1.5227E+04	8.7801E+03	6.9806E+05	4.2650E+05	1.0300E+06
	P-value	-	2.8314E-08	3.0199E-11	9.7555E-10	3.0199E-11	3.0199E-11	3.0199E-11	3.0199E-11	3.0199E-11	6.0658E-11	1.6351E-05	1.2235E-01	3.0199E-11	3.0199E-11	3.0199E-11
	Rank	2	5	9	1	10	14	11	15	13	6	4	3	12	7	8
15	Mean	3.3192E+03	**2.6655E+03**	5.9679E+03	2.4667E+04	2.1140E+04	7.2976E+07	6.6615E+04	1.9113E+10	7.7359E+06	6.0272E+03	3.5550E+03	5.1990E+03	4.5920E+03	7.1071E+03	1.2810E+09
	std	1.8350E+03	6.9741E+02	4.2709E+03	1.1105E+04	6.0205E+04	1.0825E+08	4.3588E+04	3.7389E+09	1.7433E+07	4.5043E+03	1.3813E+03	2.5498E+03	2.8074E+03	4.1777E+03	4.3775E+09
	P-value	-	9.5873E-01	6.5486E-04	3.0199E-11	1.1077E-06	3.0199E-11	3.0199E-11	3.0199E-11	3.0199E-11	5.5611E-04	1.2597E-01	9.0307E-04	1.7649E-02	9.5139E-06	1.3017E-03
	Rank	2	1	5	11	10	14	12	15	13	6	3	7	4	9	8
16	Mean	4.6672E+03	5.0164E+03	5.4465E+03	5.3035E+03	6.6457E+03	5.8356E+03	6.6127E+03	1.8840E+04	8.0065E+03	7.5641E+03	5.6012E+03	4.8812E+03	**4.2250E+03**	5.1669E+03	5.4145E+03
	std	6.3395E+02	5.6162E+02	8.0400E+02	6.3446E+02	7.8314E+02	6.8073E+02	7.1112E+02	2.7629E+03	1.1567E+03	5.0490E+02	1.0635E+03	3.9254E+02	6.9239E+02	8.3369E+02	8.5774E+02
	P-value	-	4.5146E-02	3.7704E-04	2.5306E-04	7.3891E-11	1.2541E-07	3.4742E-10	3.4742E-10	3.6897E-11	3.0199E-11	6.3560E-05	2.6433E-01	1.1228E-02	1.5638E-02	8.5641E-04
	Rank	2	4	8	6	12	10	11	15	13	14	9	3	1	5	7
17	Mean	**3.6858E+03**	4.1997E+03	4.9308E+03	4.3581E+03	5.9345E+03	4.6166E+03	6.0740E+03	3.7914E+06	7.8706E+03	4.9025E+03	4.7129E+03	4.1518E+03	4.1974E+03	4.5150E+03	6.5074E+04
	std	4.2715E+02	4.0827E+02	6.0258E+02	3.6518E+02	5.7481E+02	4.4766E+02	7.7668E+02	2.5636E+06	6.9384E+02	2.5022E+02	5.0774E+02	3.3095E+02	6.3077E+02	4.4706E+02	2.4854E+05
	P-value	-	6.3560E-05	2.6695E-09	1.8731E-07	3.0199E-11	6.5183E-09	3.0199E-11	3.0199E-11	3.0199E-01	3.3384E-11	1.5581E-08	3.5923E-05	9.0307E-04	2.1947E-08	3.3384E-11

	Rank	1	2	10	5	12	7	13	15	14	9	8	3	4	6	11
18	Mean	**5.4708E+04**	6.7093E+04	2.6784E+06	6.4620E+04	1.8816E+06	4.5289E+06	2.1382E+06	8.1832E+07	1.1499E+07	1.7752E+05	1.1225E+05	7.0259E+04	2.7516E+06	1.0783E+06	1.7551E+06
	std	3.1448E+04	3.9081E+04	1.6403E+06	1.4006E+04	1.1753E+06	2.7804E+06	1.1482E+06	4.3668E+07	7.6695E+06	4.9429E+04	5.6283E+04	3.1251E+04	1.1406E+06	8.1225E+05	2.8767E+06
	P-value	-	2.3399E-01	3.0199E-11	1.4412E-02	3.0199E-11	3.0199E-11	3.0199E-11	3.0199E-11	3.0199E-11	6.6955E-11	5.0912E-06	2.6077E-02	3.0199E-11	3.0199E-11	3.0199E-11
	Rank	1	3	11	2	9	13	10	15	14	6	5	4	12	7	8
19	Mean	4.6422E+03	**3.1904E+03**	4.9750E+03	2.3100E+04	6.9283E+03	1.2853E+08	1.2337E+05	1.8680E+10	1.9726E+07	3.5326E+03	4.0794E+03	4.1740E+03	5.7750E+03	5.4962E+03	5.0620E+08
	std	3.3119E+03	1.1834E+03	3.9370E+03	1.5116E+04	4.6906E+03	1.9477E+08	6.0431E+04	3.7014E+09	2.0987E+07	1.2307E+03	2.5626E+03	2.2410E+03	4.5988E+03	3.8659E+03	1.6496E+09
	P-value	-	9.6263E-02	9.3519E-01	4.1997E-10	8.6844E-03	3.0199E-11	3.0199E-11	3.0199E-11	3.0199E-11	4.9178E-01	4.2896E-01	1.0000E+00	5.9969E-01	4.2896E-01	6.1452E-02
	Rank	5	1	6	11	9	14	12	15	13	3	2	4	8	7	10
20	Mean	**3.6333E+03**	4.1424E+03	4.9759E+03	4.3914E+03	5.9201E+03	4.8501E+03	5.8688E+03	6.9573E+03	6.0173E+03	5.4415E+03	5.4094E+03	4.5442E+03	5.2902E+03	4.5513E+03	4.8998E+03
	std	4.7891E+02	3.8137E+02	5.7983E+02	3.3362E+02	5.0865E+02	7.6634E+02	5.2913E+02	3.2965E+02	5.6936E+02	2.7230E+02	5.0968E+02	2.3695E+02	1.1182E+03	5.7346E+02	5.7036E+02
	P-value	-	4.0840E-05	2.8716E-10	4.6856E-08	3.6897E-11	8.1014E-10	3.0199E-11	3.0199E-11	3.0199E-11	3.0199E-11	1.7769E-10	9.7555E-10	3.1967E-09	2.3768E-07	2.9215E-09
	Rank	1	2	8	3	13	6	12	15	14	11	10	4	9	5	7
21	Mean	**2.5139E+03**	2.5921E+03	2.6825E+03	2.6538E+03	3.7451E+03	2.9277E+03	3.5197E+03	4.5153E+03	3.5507E+03	3.0982E+03	2.7126E+03	2.7110E+03	2.6858E+03	2.6017E+03	2.9884E+03
	std	3.2900E+01	3.8818E+01	6.1981E+01	5.5330E+01	2.5425E+02	1.1921E+02	1.6484E+02	1.8060E+02	1.3295E+02	3.5867E+01	6.4378E+01	4.4395E+01	8.1648E+01	6.0327E+01	2.0613E+02
	P-value	-	1.5465E-09	3.6897E-11	1.3289E-10	3.0199E-11	3.0199E-11	3.0199E-11	3.0199E-11	3.0199E-11	3.0199E-11	5.5727E-10	3.0199E-11	3.3384E-11	1.6980E-08	3.0199E-11
	Rank	1	2	6	4	14	9	12	15	13	11	8	7	5	3	10
22	Mean	**4.6908E+03**	1.6882E+04	2.0418E+04	1.5258E+04	2.0097E+04	1.8993E+04	1.9586E+04	3.2399E+04	2.0981E+04	2.8808E+04	2.1048E+04	2.1198E+04	2.1134E+04	1.8219E+04	1.8476E+04
	std	5.4564E+03	1.5741E+03	2.3094E+03	5.2827E+03	1.3522E+03	2.9507E+03	1.5698E+03	7.8077E+02	2.0001E+03	1.4475E+03	2.5926E+03	1.5444E+03	4.0126E+03	1.9774E+03	3.1902E+03
	P-value	-	1.6980E-08	1.0937E-10	1.6980E-08	4.5043E-11	3.4742E-10	8.1527E-11	8.1527E-11	4.9752E-11	3.0199E-11	6.6955E-11	3.0199E-11	1.7769E-10	1.5465E-09	1.5465E-09
	Rank	1	3	9	2	10	6	7	15	11	14	13	12	8	5	4
23	Mean	**3.0319E+03**	3.0942E+03	3.1698E+03	3.1928E+03	4.2919E+03	3.4767E+03	4.0182E+03	6.8519E+03	4.2536E+03	3.3331E+03	3.1619E+03	3.2194E+03	3.1941E+03	3.3117E+03	3.7418E+03
	std	3.4673E+01	3.0369E+01	5.7181E+01	5.4446E+01	2.2490E+02	8.5979E+01	1.8708E+02	4.6005E+02	1.3524E+02	1.6643E+02	2.5555E+01	4.0550E+01	5.5179E+01	1.3873E+02	6.8207E+02
	P-value	-	6.0104E-08	1.9568E-10	3.0199E-11	3.0199E-11	3.0199E-11	3.0199E-11	3.0199E-11	3.0199E-11	3.0199E-11	3.0199E-11	3.0199E-11	3.0199E-11	3.0199E-11	3.0199E-11
	Rank	1	2	4	5	14	10	12	15	13	8	3	7	6	9	11
24	Mean	**3.4647E+03**	3.5558E+03	3.6287E+03	3.7290E+03	5.1271E+03	4.0261E+03	5.0360E+03	1.0612E+04	5.0252E+03	4.0748E+03	3.7076E+03	3.6737E+03	3.5605E+03	3.9123E+03	4.2766E+03
	std	3.2767E+01	4.8449E+01	7.0050E+01	5.1934E+01	3.2213E+02	1.4841E+02	3.1289E+02	6.7915E+02	2.2524E+02	5.1901E+01	6.0126E+01	9.9966E+01	6.1445E+01	1.6861E+02	3.7187E+02
	P-value	-	2.0338E-09	5.4941E-11	3.0199E-11	3.0199E-11	3.0199E-11	3.0199E-11	3.0199E-11	3.0199E-11	3.0199E-11	3.0199E-11	5.4941E-11	5.4617E-09	3.0199E-11	3.0199E-11
	Rank	1	3	4	7	14	9	13	15	12	10	6	5	2	8	11

25	Mean	**3.3434E+03**	3.4095E+03	3.4370E+03	3.5312E+03	3.4847E+03	5.4212E+03	3.5074E+03	2.6980E+04	6.1423E+03	3.5339E+03	3.3512E+03	3.3513E+03	3.3966E+03	3.9530E+03	6.2136E+03
	std	4.3649E+01	5.0936E+01	5.4958E+01	6.2378E+01	5.4865E+01	4.9686E+02	7.0608E+01	3.1049E+03	3.1655E+03	4.3633E+01	6.1081E+01	4.4645E+01	4.0706E+01	1.5689E+02	5.2714E+03
	P-value	-	6.7362E-06	4.6856E-08	4.9752E-11	1.6132E-10	3.0199E-11	3.1589E-10	3.1589E-10	3.0199E-11	3.0199E-11	5.9969E-01	4.5530E-01	1.5292E-05	3.0199E-11	1.0188E-05
	Rank	1	5	6	9	7	14	8	15	13	10	2	3	4	12	11
26	Mean	**7.8733E+03**	1.0216E+04	1.0074E+04	1.0018E+04	2.3581E+04	1.3166E+04	2.2606E+04	5.1491E+04	2.4215E+04	1.4611E+04	9.7197E+03	9.9365E+03	8.3367E+03	8.5536E+03	1.6183E+04
	std	1.7175E+03	2.8694E+03	1.0985E+03	9.5918E+02	4.4854E+03	1.0739E+03	2.5935E+03	3.7778E+03	2.3950E+03	8.1389E+02	8.4603E+02	6.9610E+02	1.5687E+03	1.9980E+03	8.7701E+03
	P-value	-	8.4848E-09	6.1210E-10	7.1186E-09	1.3289E-10	5.0723E-10	3.0199E-11	3.0199E-11	3.0199E-11	3.4742E-10	1.3111E-08	2.2273E-09	4.3531E-05	8.1465E-05	3.2555E-07
	Rank	1	4	6	8	13	9	12	15	14	11	5	7	2	3	10
27	Mean	**3.4218E+03**	3.4724E+03	3.4717E+03	3.5560E+03	3.9489E+03	3.9223E+03	4.0369E+03	1.2186E+04	3.9767E+03	3.8314E+03	3.4858E+03	3.4820E+03	3.4694E+03	3.8369E+03	3.9926E+03
	std	4.6547E+01	4.8566E+01	5.2188E+01	7.3284E+01	3.2576E+02	1.3638E+02	1.9029E+02	1.2029E+03	2.1022E+02	1.1932E+02	6.4604E+01	6.5670E+01	4.2447E+01	8.5274E+01	9.6363E+02
	P-value	-	8.1465E-05	8.6634E-05	1.5465E-09	3.0199E-11	3.0199E-11	3.0199E-11	3.0199E-11	3.3384E-11	3.0199E-11	2.1327E-05	2.2539E-04	1.1058E-04	3.0199E-11	1.6132E-10
	Rank	1	3	4	7	11	12	14	15	13	9	5	6	2	10	8
28	Mean	3.4675E+03	3.5168E+03	3.5494E+03	3.5813E+03	3.5732E+03	6.9059E+03	3.5981E+03	3.2821E+04	1.9252E+04	3.6906E+03	**3.4576E+03**	3.4729E+03	3.4964E+03	4.0872E+03	8.5301E+03
	std	3.2379E+01	4.2564E+01	3.6733E+01	3.7550E+01	5.0062E+01	8.9409E+02	4.6117E+01	2.5274E+03	3.3084E+03	3.7310E+01	3.1310E+01	3.6404E+01	3.3493E+01	1.6999E+02	7.3854E+03
	P-value	-	9.5139E-06	5.0723E-10	3.6897E-11	2.1544E-10	3.0199E-11	4.9752E-11	4.9752E-11	3.0199E-11	3.0199E-11	2.5188E-01	4.7335E-01	1.6798E-03	3.0199E-11	5.0912E-06
	Rank	2	5	6	8	7	13	9	15	14	11	1	3	4	12	10
F29	Mean	**5.3670E+03**	6.2457E+03	6.0420E+03	6.6188E+03	7.9240E+03	7.8590E+03	8.1815E+03	3.2389E+05	1.0244E+04	8.2828E+03	6.0795E+03	5.8702E+03	6.3067E+03	6.8687E+03	8.7943E+03
	std	3.9098E+02	5.6792E+02	5.9624E+02	4.5021E+02	7.0903E+02	5.4729E+02	6.4753E+02	1.6250E+05	1.4365E+03	3.0325E+02	6.1240E+02	4.7455E+02	6.7453E+02	5.7090E+02	6.4154E+03
	P-value	-	5.0922E-08	1.1674E-05	1.4643E-10	3.0199E-11	3.0199E-11	3.0199E-11	3.0199E-11	3.0199E-11	3.0199E-11	9.5139E-06	7.1988E-05	5.1857E-07	6.6955E-11	4.9752E-11
	Rank	1	5	3	7	11	10	12	15	14	13	4	2	6	8	9
30	Mean	**1.3147E+04**	2.2078E+04	4.7162E+04	1.3696E+06	2.8988E+05	4.9454E+08	2.4094E+06	3.4777E+10	5.6891E+07	3.8716E+05	2.0885E+04	5.8213E+04	9.4163E+04	1.1156E+06	1.3459E+09
	std	3.8169E+03	4.4376E+03	3.5447E+04	5.7201E+05	1.5074E+05	3.2740E+08	9.9940E+05	6.1187E+09	9.1657E+07	1.6509E+05	1.0355E+04	2.8969E+04	5.5256E+04	1.5436E+06	5.6451E+09
	P-value	-	7.1186E-09	8.8910E-10	3.0199E-11	3.0199E-11	3.0199E-11	3.0199E-11	3.0199E-11	3.0199E-11	3.0199E-11	1.4423E-03	3.6897E-11	3.0199E-11	3.0199E-11	1.0702E-09
	Rank	1	3	4	11	8	14	12	15	13	9	2	5	6	10	7
MeanRank		**1.4828**	3.6897	6.4828	6.7241	10.9310	11.0345	10.8276	14.9310	13.2069	9.3103	4.7931	5.0345	6.1724	6.6207	8.7586
Rank		**1**	2	6	8	12	13	11	15	14	10	3	4	5	7	9

Table S4 AROA variants based on four strategies

Srategy	IAROA	AROA	AROA1	AROA2	AROA3	AROA4	AROA5	AROA6	AROA7	AROA8	AROA9	AROA10	AROA11	AROA12	AROA13	AROA14
Strategy1	1	0	1	0	0	0	1	1	1	0	0	0	1	1	1	0
Strategy2	1	0	0	1	0	0	1	0	0	1	1	0	1	1	0	1
Strategy3	1	0	0	0	1	0	0	1	0	1	0	1	1	0	1	1
Strategy4	1	0	0	0	0	1	0	0	1	0	1	1	0	1	1	1

Table S5 Results of AROA ablation experiments based on four strategies

F	IAROA	AROA	AROA1	AROA2	IAROA3	AROA4	AROA5	AROA6	AROA7	AROA8	AROA9	AROA10	AROA11	AROA12	AROA13	AROA14
1	1.9036E+02	4.4300E+03	3.0988E+03	2.0061E+03	2.4546E+03	2.3875E+03	1.6777E+03	1.2714E+03	5.5840E+03	4.0681E+02	6.3349E+02	1.6118E+03	2.5643E+02	1.2413E+03	3.2532E+03	1.5547E+02
3	3.0000E+02	3.0009E+02	3.0010E+02	3.0000E+02	3.0001E+02	3.0019E+02	3.0000E+02	3.0001E+02	3.0008E+02	3.0000E+02	3.0000E+02	3.0001E+02	3.0000E+02	3.0000E+02	3.0002E+02	3.0000E+02
4	4.1647E+02	4.4394E+02	4.2802E+02	4.2697E+02	4.3684E+02	4.4844E+02	4.0244E+02	4.2974E+02	4.5459E+02	4.0624E+02	4.0635E+02	4.4470E+02	4.0320E+02	4.2924E+02	4.2098E+02	4.2512E+02
5	5.2965E+02	5.4119E+02	5.4955E+02	5.4517E+02	5.2925E+02	5.3323E+02	5.4040E+02	5.4199E+02	5.2905E+02	5.3045E+02	5.2726E+02	5.2567E+02	5.3642E+02	5.2249E+02	5.2865E+02	5.2209E+02
6	6.0000E+02	6.0002E+02	6.0002E+02	6.0000E+02	6.0002E+02	6.0001E+02	6.0000E+02	6.0002E+02	6.0002E+02	6.0000E+02	6.0000E+02	6.0001E+02	6.0000E+02	6.0000E+02	6.0001E+02	6.0000E+02
7	7.5042E+02	7.6311E+02	7.6951E+02	7.6084E+02	7.6494E+02	7.5608E+02	7.5788E+02	7.6289E+02	7.5175E+02	7.6386E+02	7.5169E+02	7.5373E+02	7.7010E+02	7.5312E+02	7.5969E+02	7.5771E+02
8	8.2527E+02	8.4557E+02	8.4020E+02	8.4059E+02	8.4955E+02	8.3144E+02	8.3900E+02	8.4000E+02	8.1851E+02	8.4716E+02	8.2965E+02	8.2766E+02	8.3801E+02	8.3244E+02	8.3239E+02	8.2566E+02
9	9.0007E+02	9.0574E+02	9.0267E+02	9.0078E+02	9.0121E+02	9.0052E+02	9.0064E+02	9.0102E+02	9.0078E+02	9.0082E+02	9.0024E+02	9.0025E+02	9.0051E+02	9.0053E+02	9.0052E+02	9.0009E+02
10	3.3184E+03	3.4701E+03	3.3410E+03	3.7461E+03	3.1560E+03	3.2299E+03	3.5202E+03	3.3350E+03	3.4416E+03	3.4104E+03	3.1757E+03	2.9626E+03	3.5207E+03	3.0159E+03	2.8218E+03	2.9789E+03
11	1.1094E+03	1.1245E+03	1.1384E+03	1.1228E+03	1.1461E+03	1.1343E+03	1.1287E+03	1.1149E+03	1.1087E+03	1.1239E+03	1.1316E+03	1.1232E+03	1.1155E+03	1.1333E+03	1.1135E+03	1.1233E+03
12	1.0976E+04	4.6813E+04	2.7766E+04	4.2119E+04	2.0979E+04	2.6585E+04	2.5055E+04	1.7235E+04	5.0065E+04	1.9530E+04	2.2540E+04	9.4609E+03	1.4924E+04	2.2817E+04	1.3413E+04	1.2073E+04
13	1.4150E+03	8.1544E+03	2.4369E+03	1.7845E+03	1.4127E+03	1.4092E+03	2.8686E+03	1.4398E+03	2.1967E+03	1.6983E+03	1.7267E+03	1.3660E+03	1.3344E+03	4.9505E+03	2.0677E+03	1.4308E+03
14	1.4127E+03	1.4370E+03	1.4199E+03	1.4210E+03	1.4182E+03	1.4260E+03	1.4250E+03	1.4191E+03	1.4174E+03	1.4263E+03	1.4254E+03	1.4198E+03	1.4267E+03	1.4120E+03	1.4251E+03	1.4196E+03
15	1.5185E+03	1.5991E+03	1.5322E+03	1.5221E+03	1.5305E+03	1.5117E+03	1.5203E+03	1.5243E+03	1.5135E+03	1.5148E+03	1.5150E+03	1.5098E+03	1.5181E+03	1.5158E+03	1.5091E+03	1.5119E+03
16	1.9239E+03	2.0489E+03	2.1557E+03	1.9145E+03	1.9167E+03	1.9091E+03	1.9958E+03	1.9767E+03	2.1670E+03	1.9316E+03	1.9353E+03	2.0307E+03	2.0347E+03	2.0653E+03	2.0099E+03	1.8568E+03
17	1.7419E+03	1.8383E+03	1.7636E+03	1.7798E+03	1.7829E+03	1.7820E+03	1.7665E+03	1.7641E+03	1.7482E+03	1.7732E+03	1.7683E+03	1.7564E+03	1.7618E+03	1.7404E+03	1.7853E+03	1.7447E+03
18	1.8161E+03	1.8522E+03	1.8283E+03	1.8270E+03	1.8351E+03	1.8322E+03	1.8356E+03	1.8389E+03	1.8250E+03	1.8335E+03	1.8298E+03	1.8306E+03	1.8275E+03	1.8248E+03	1.8240E+03	1.8162E+03
19	1.9059E+03	1.9198E+03	1.9096E+03	1.9101E+03	1.9106E+03	1.9072E+03	1.9143E+03	1.9103E+03	1.9081E+03	1.9115E+03	1.9073E+03	1.9062E+03	1.9133E+03	1.9056E+03	1.9073E+03	1.9083E+03
20	2.0511E+03	2.1349E+03	2.1880E+03	2.0795E+03	2.1380E+03	2.0513E+03	2.1035E+03	2.1236E+03	2.1150E+03	2.1522E+03	2.0819E+03	2.0560E+03	2.0878E+03	2.0959E+03	2.0340E+03	2.1031E+03
21	2.3217E+03	2.3465E+03	2.3484E+03	2.3315E+03	2.3334E+03	2.3295E+03	2.3057E+03	2.3366E+03	2.3250E+03	2.3383E+03	2.3226E+03	2.3299E+03	2.3322E+03	2.3224E+03	2.3212E+03	2.3217E+03
22	2.3000E+03	2.3000E+03	2.3018E+03	2.3000E+03	2.3005E+03	2.3005E+03	2.3000E+03	2.3005E+03	2.3001E+03	2.3000E+03	2.3000E+03	2.3007E+03	2.3000E+03	2.3000E+03	2.3012E+03	2.3000E+03
23	2.6762E+03	2.6910E+03	2.6881E+03	2.6829E+03	2.6817E+03	2.6815E+03	2.6191E+03	2.6806E+03	2.6761E+03	2.6818E+03	2.6695E+03	2.6788E+03	2.6794E+03	2.6728E+03	2.6837E+03	2.6715E+03
24	2.8475E+03	2.8588E+03	2.8514E+03	2.8539E+03	2.8473E+03	2.8492E+03	2.8524E+03	2.8630E+03	2.8528E+03	2.8685E+03	2.8520E+03	2.8494E+03	2.8473E+03	2.8452E+03	2.8600E+03	2.8550E+03

25	2.8861E+03	2.8872E+03	2.8865E+03	2.8864E+03	2.8864E+03	2.8868E+03	2.8862E+03	2.8866E+03	2.8874E+03	2.8873E+03	2.8855E+03	2.8871E+03	2.8841E+03	2.8862E+03	2.8870E+03	2.8863E+03
26	3.1160E+03	3.9861E+03	3.5244E+03	3.9691E+03	3.7698E+03	3.9088E+03	2.9000E+03	3.4002E+03	3.3252E+03	3.7438E+03	3.8656E+03	3.9755E+03	3.1255E+03	3.4555E+03	3.8326E+03	3.8850E+03
27	3.1984E+03	3.1983E+03	3.1990E+03	3.1986E+03	3.1982E+03	3.2008E+03	3.2036E+03	3.2000E+03	3.2049E+03	3.1997E+03	3.2077E+03	3.2075E+03	3.1968E+03	3.2075E+03	3.1970E+03	3.1979E+03
28	3.1413E+03	3.1392E+03	3.1000E+03	3.1000E+03	3.1738E+03	3.1213E+03	3.1308E+03	3.1000E+03	3.1039E+03	3.1228E+03	3.1308E+03	3.1435E+03	3.1000E+03	3.1413E+03	3.1662E+03	3.1229E+03
29	3.3316E+03	3.3930E+03	3.3617E+03	3.3412E+03	3.3713E+03	3.3380E+03	3.3792E+03	3.3854E+03	3.3379E+03	3.3421E+03	3.3643E+03	3.3358E+03	3.3407E+03	3.3495E+03	3.3343E+03	3.3280E+03
30	5.0955E+03	5.3523E+03	5.1449E+03	5.1003E+03	5.3974E+03	5.0834E+03	5.2196E+03	5.2764E+03	5.1292E+03	5.1381E+03	5.1245E+03	5.0172E+03	5.1496E+03	5.0602E+03	5.1262E+03	5.2000E+03
Rank	1	16	15	8	13	10	11	14	12	9	4	5	6	3	7	2

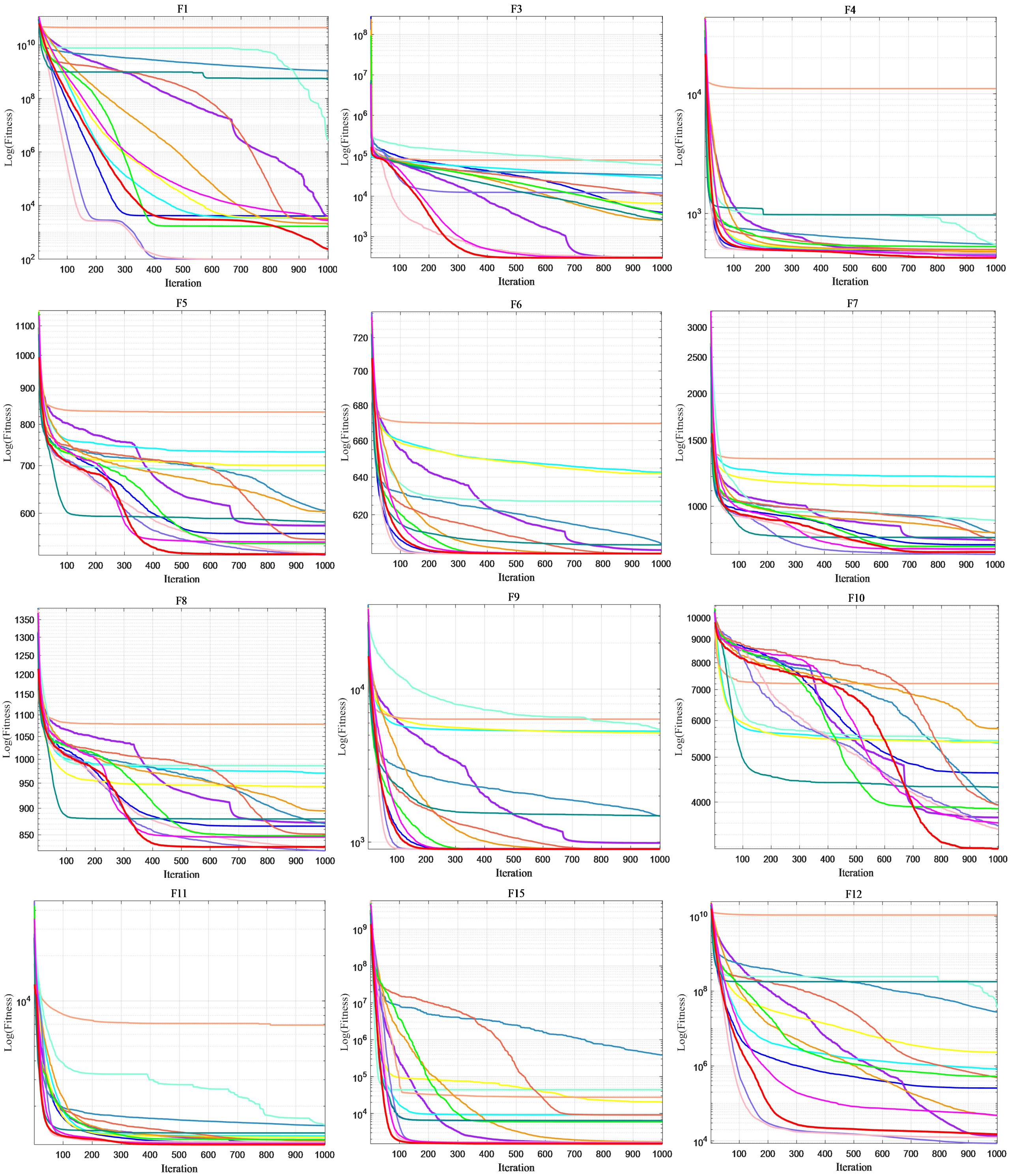
F1
F3
F4
F5
F6
F7
F8
F9
F10
F11
F15
F12
Log(Fitness)
Iteration
100 200 300 400 500 600 700 800 900 1000

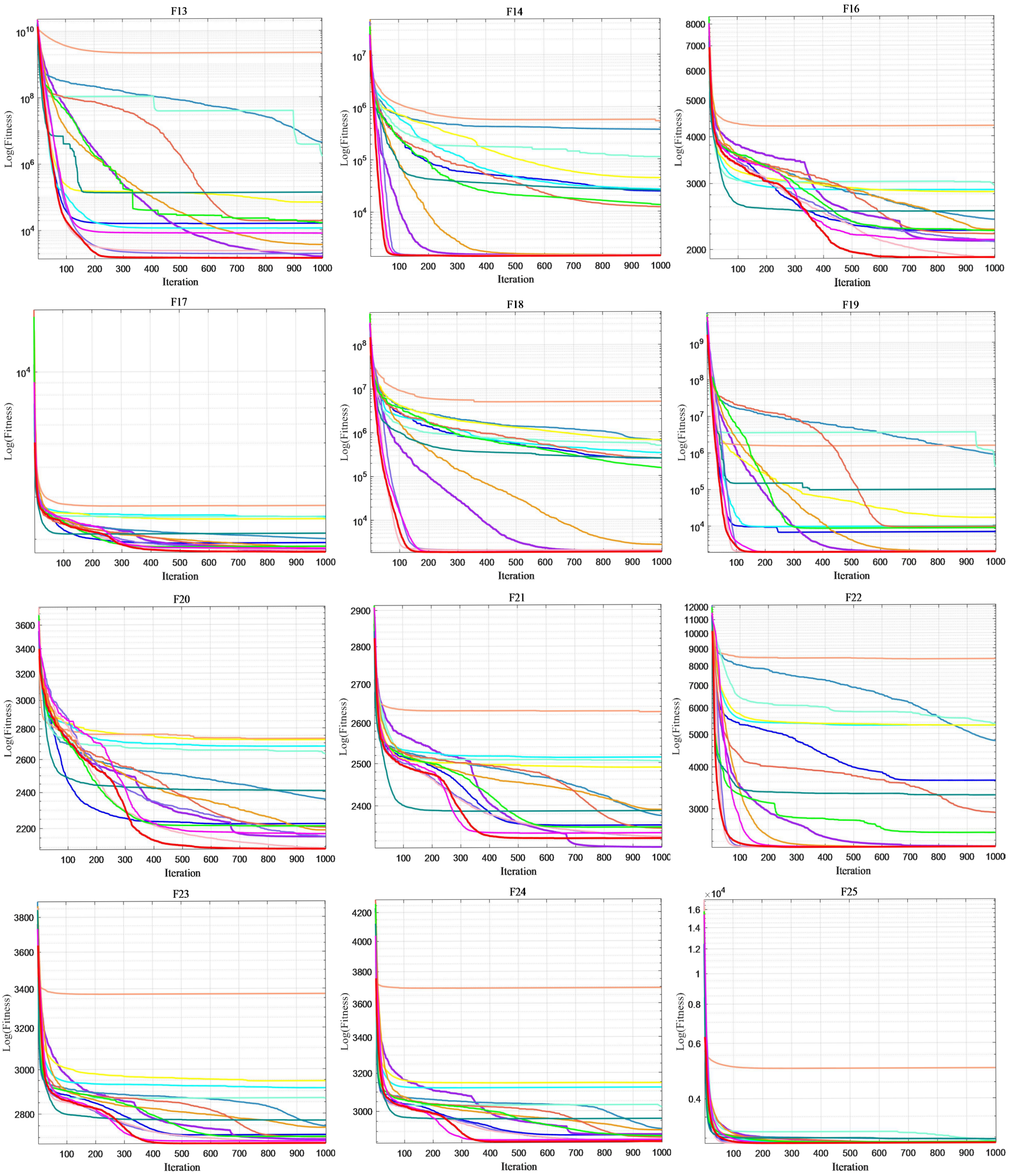
F13
F14
F16
F17
F18
F19
F20
F21
F22
F23
F24
F25
Log(Fitness)
Iteration
×10^4

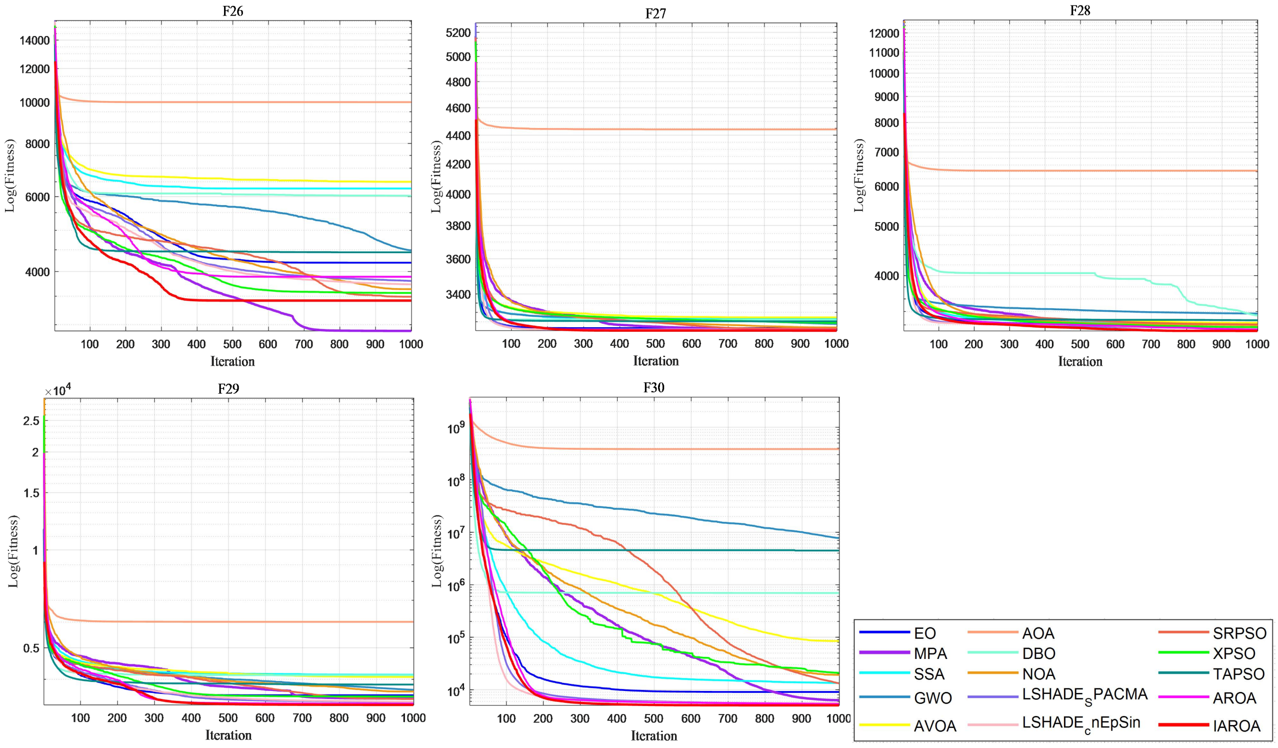

Figure S1. Convergence curve of IAROA with other algorithms for solving 30D of CEC2017.

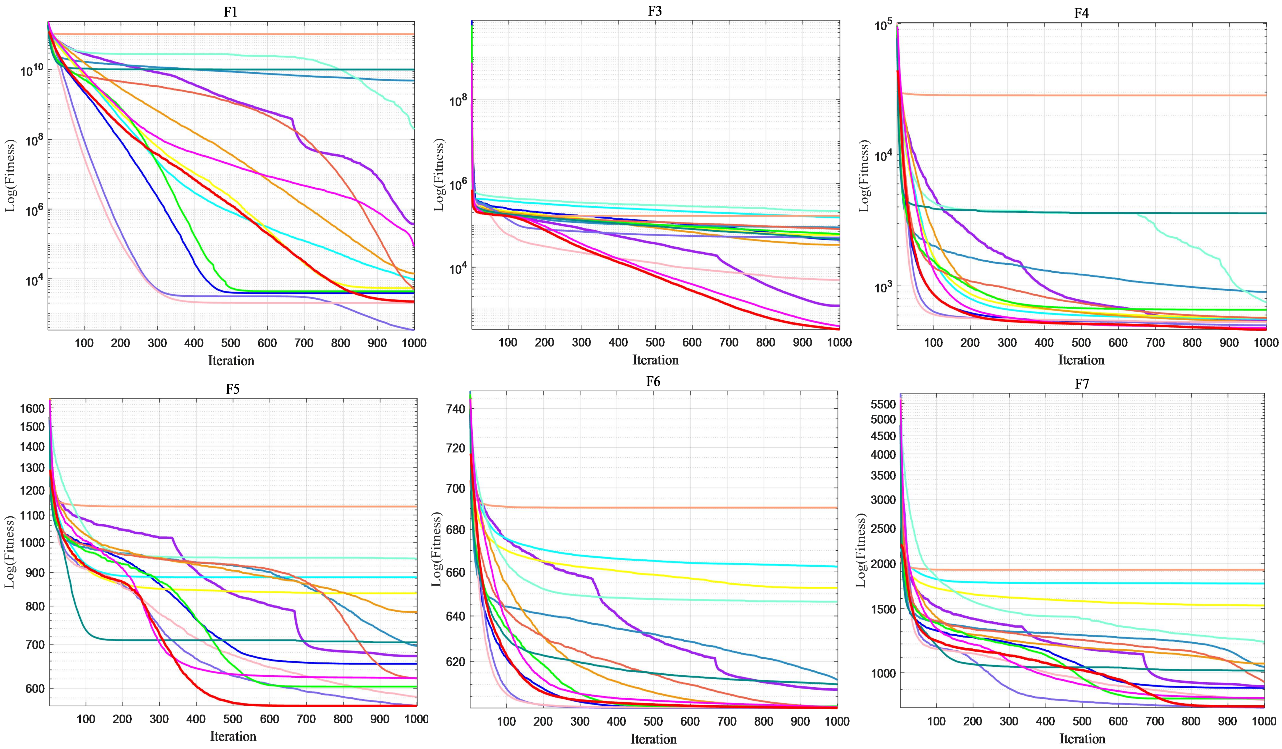

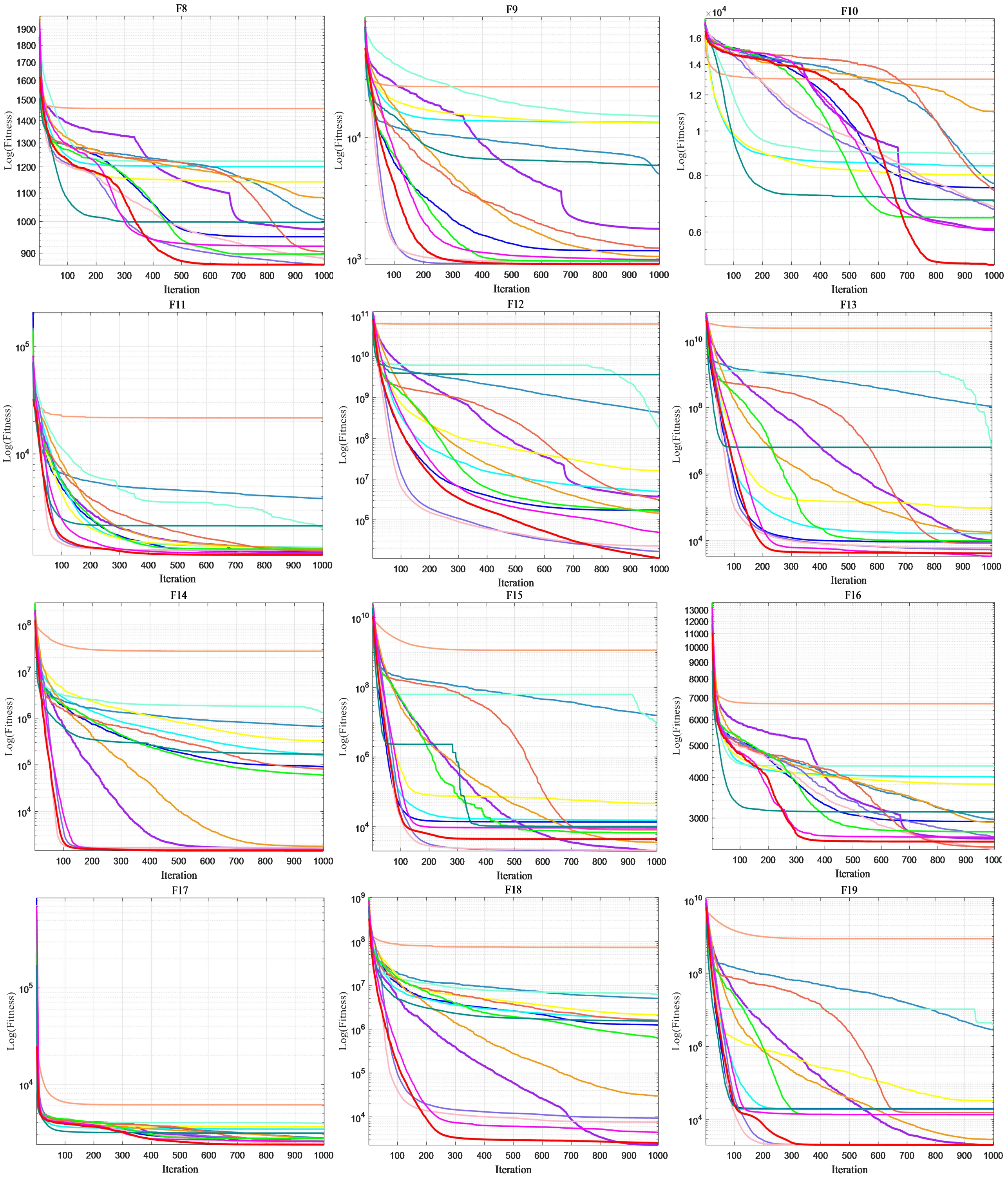
F8
F9
F10
F11
F12
F13
F14
F15
F16
F17
F18
F19
Log(Fitness)
Iteration

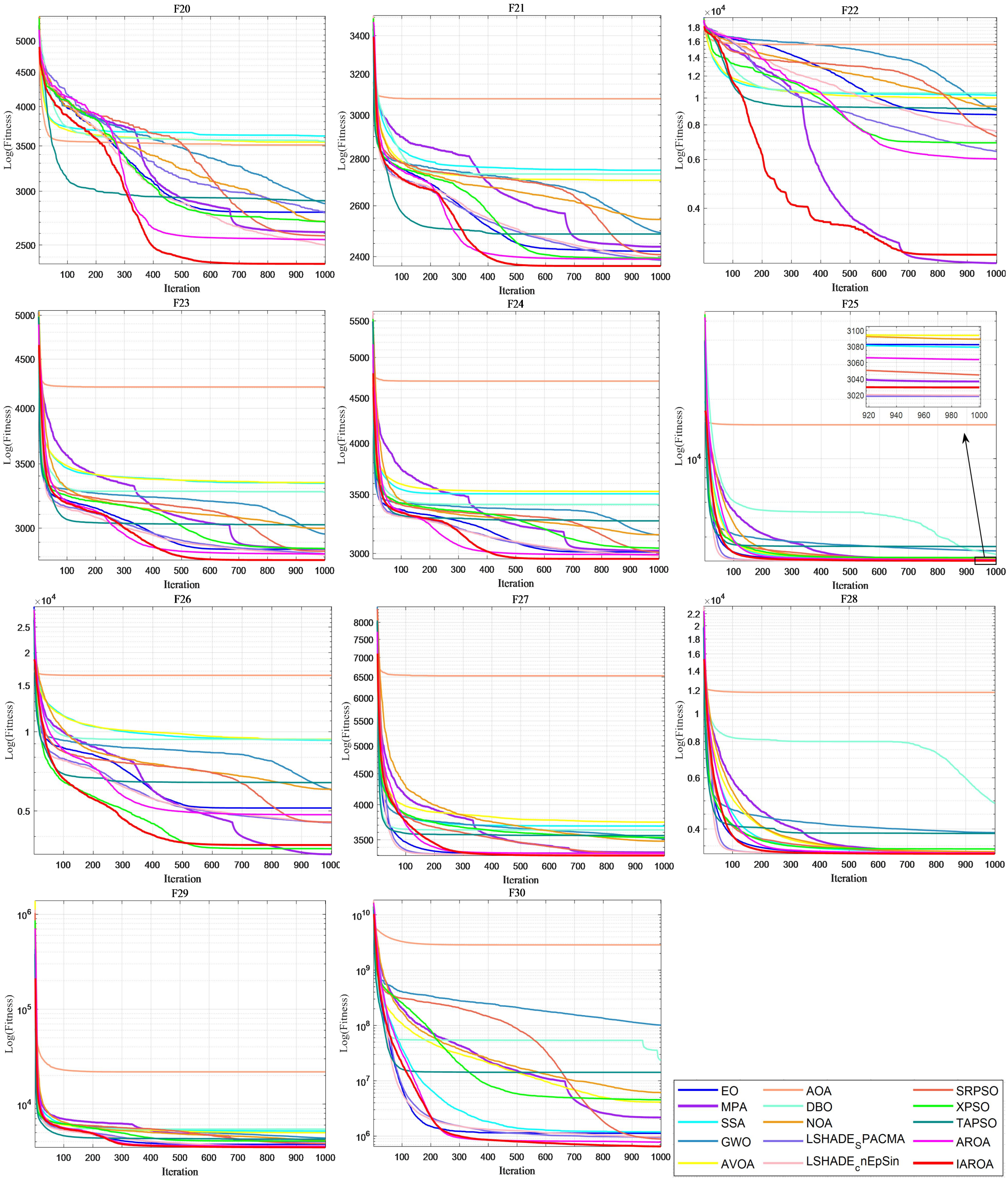

Figure S2. Convergence curve of IAROA with other algorithms for solving 50D of CEC2017.

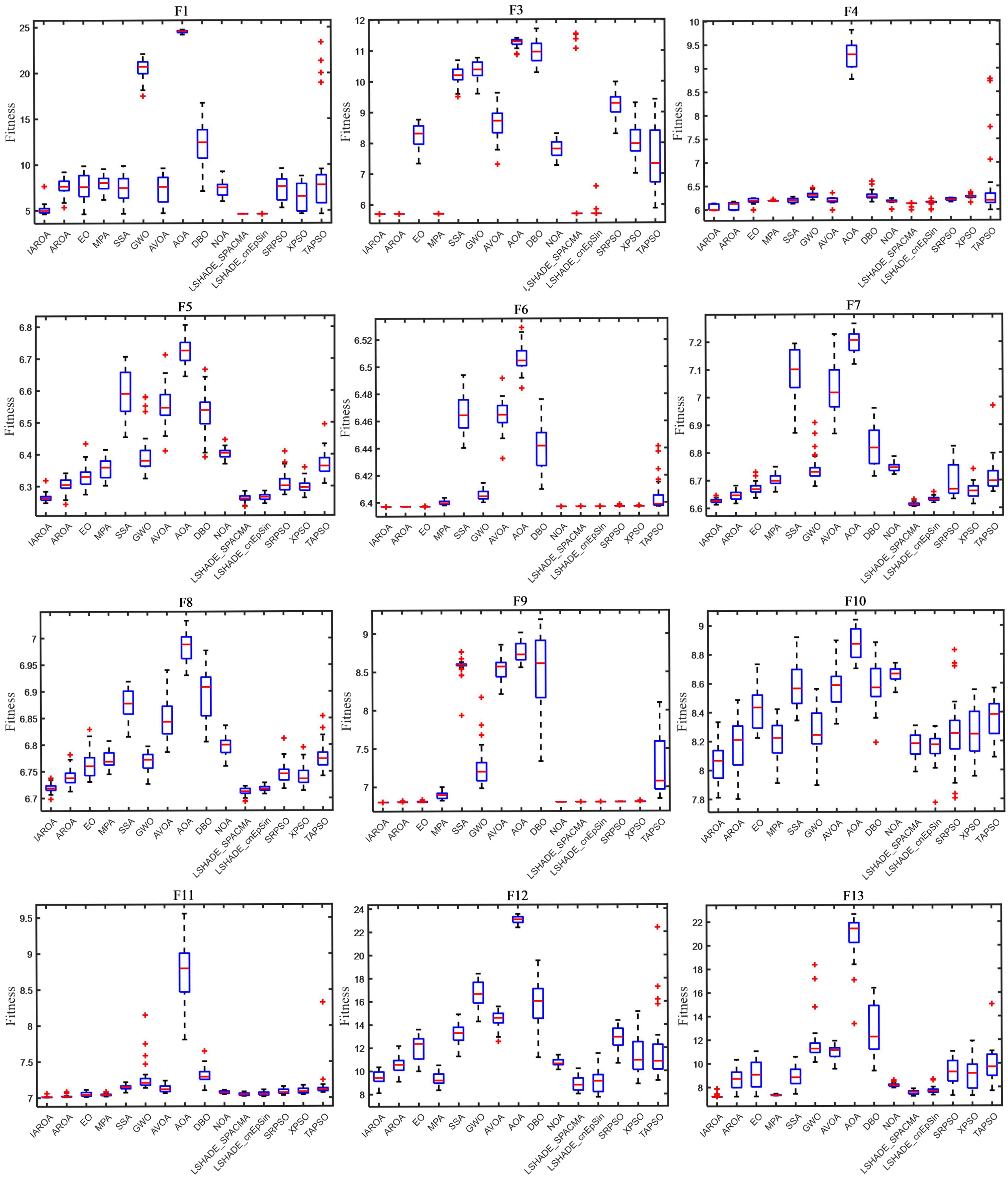
F1
F3
F4
F5
F6
F7
F8
F9
F10
F11
F12
F13
Fitness
IAROA
AROA
EO
MPA
SSA
GWO
AVOA
AOA
DBO
NOA
LSHADE_SPACMA
LSHADE_cnEpSin
SRPSO
XPSO
TAPSO

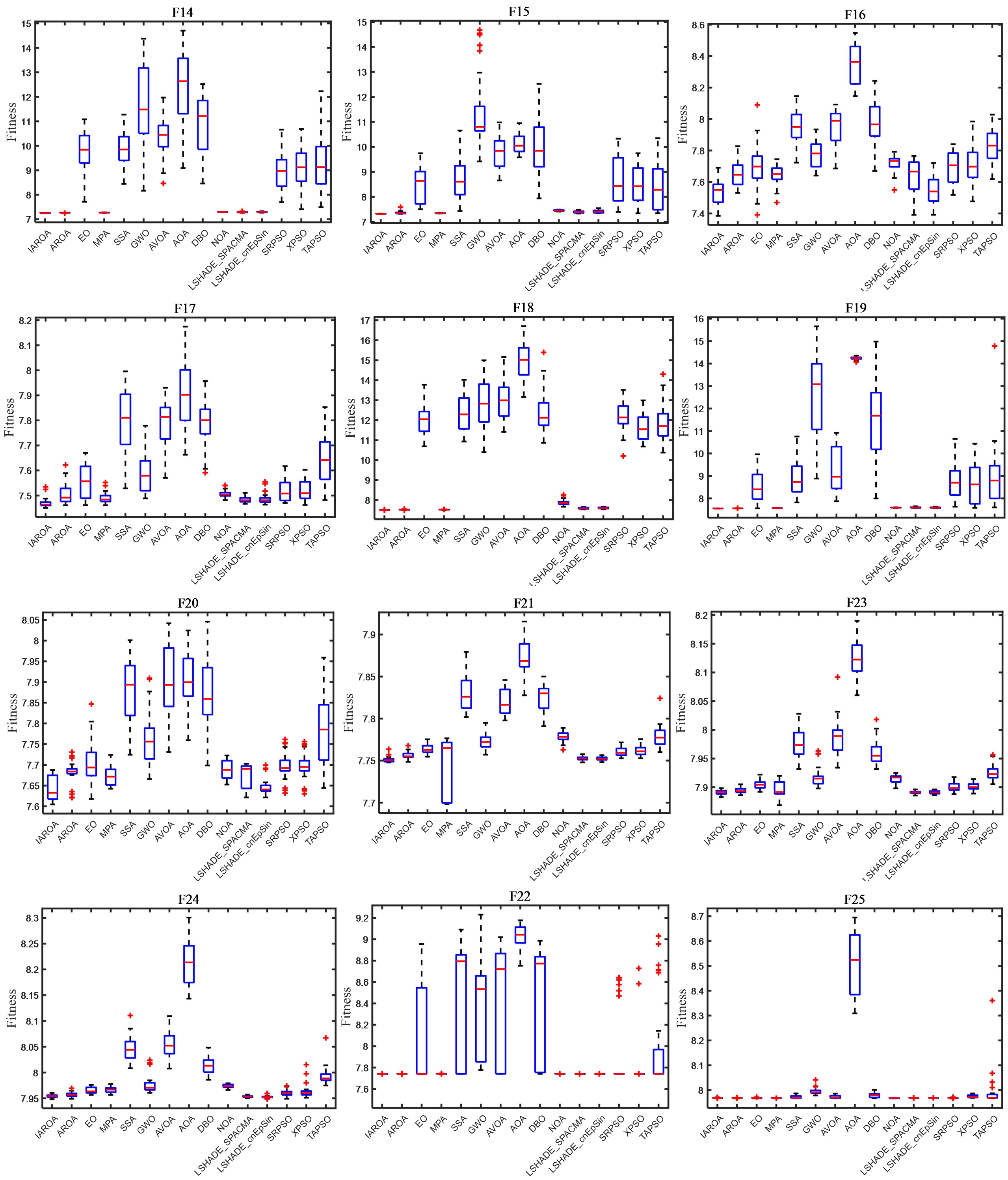
F14
F15
F16
F17
F18
F19
F20
F21
F23
F24
F22
F25
Fitness
IAROA
AROA
EO
MPA
SSA
GWO
AVOA
AOA
DBO
NOA
LSHADE_SPACMA
LSHADE_cnEpSin
SRPSO
XPSO
TAPSO

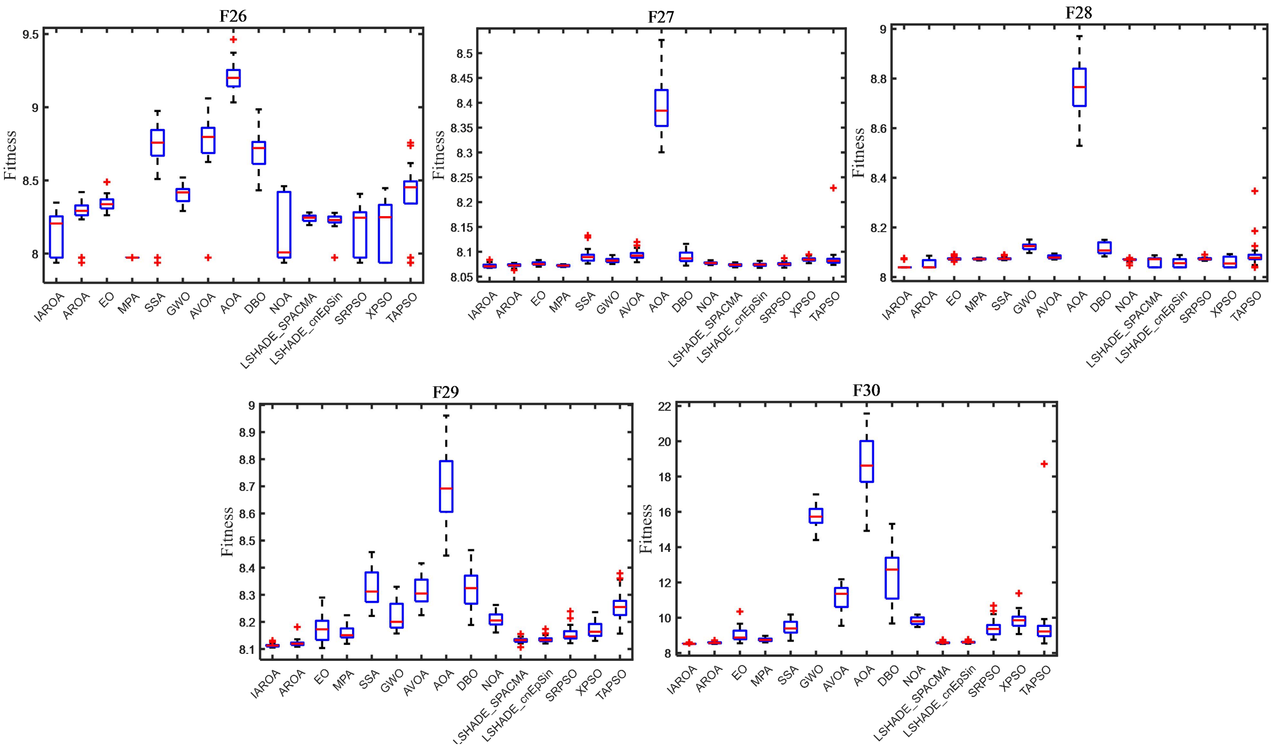

Figure S3. Boxplot of IAROA versus other algorithms for solving the 30D of CEC2017.

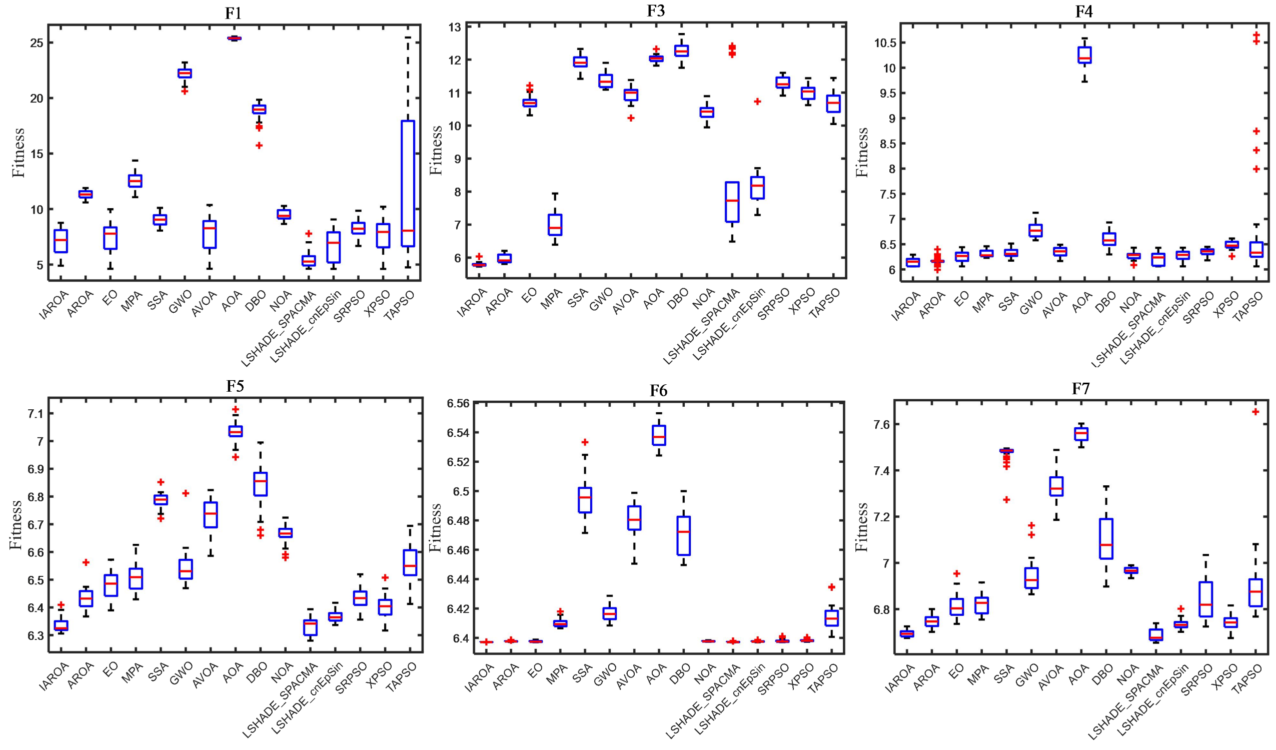

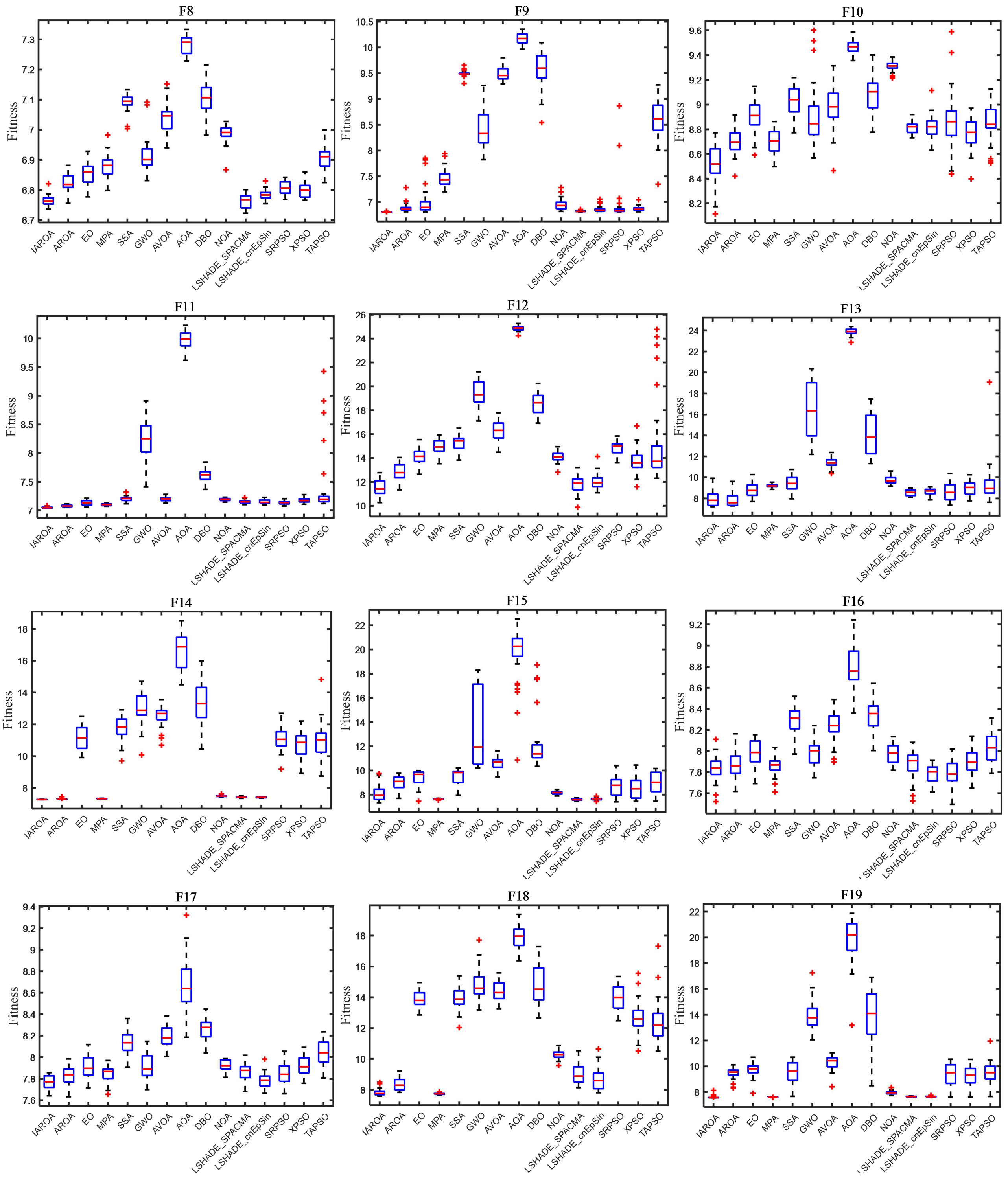
F8
F9
F10
F11
F12
F13
F14
F15
F16
F17
F18
F19
Fitness
IAROA
AROA
EO
MPA
SSA
GWO
AVOA
AOA
DBO
NOA
LSHADE_SPACMA
LSHADE_cnEpSin
SRPSO
XPSO
TAPSO

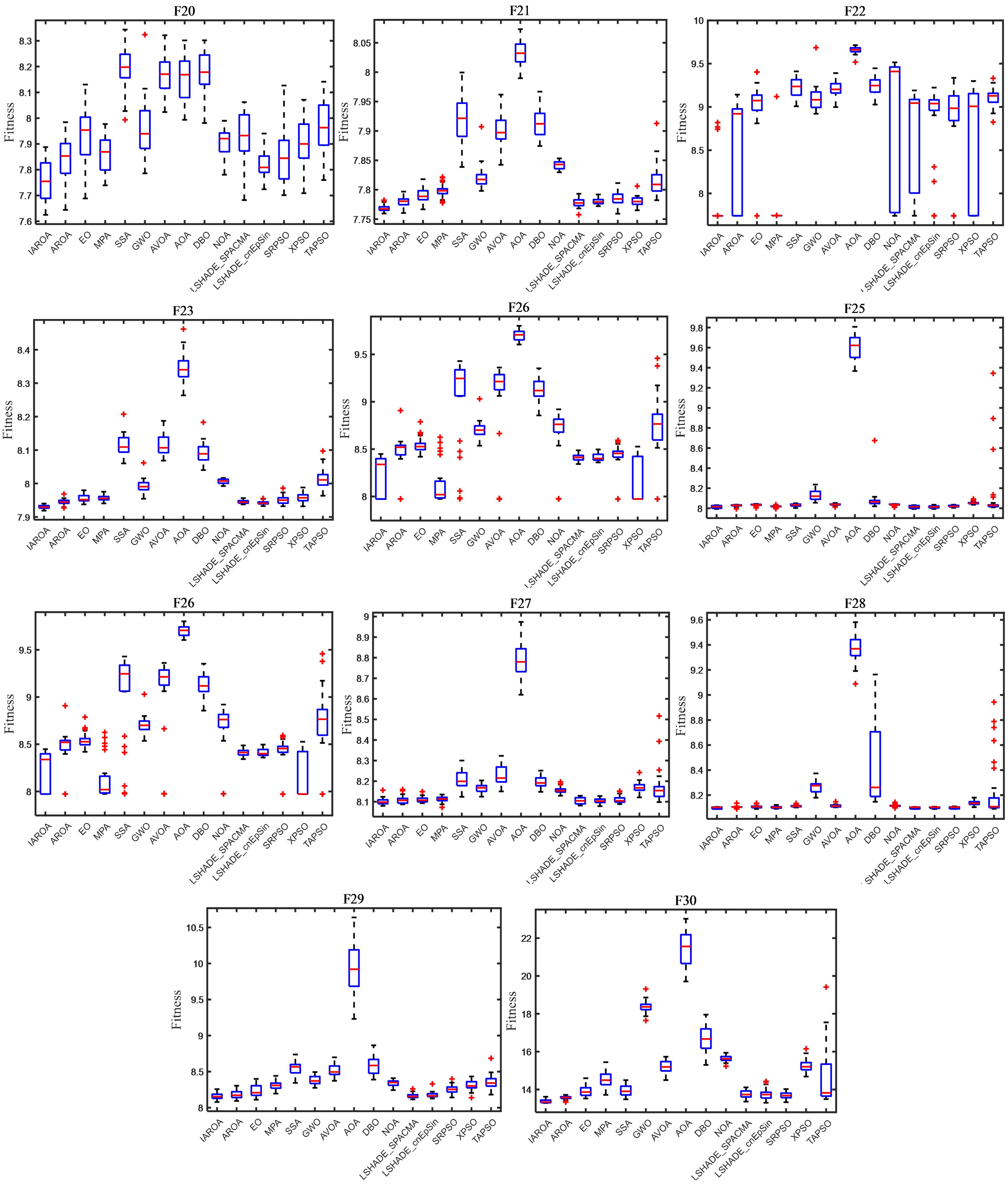

Figure S4. Boxplot of IAROA versus other algorithms solving the 50D CEC2017.

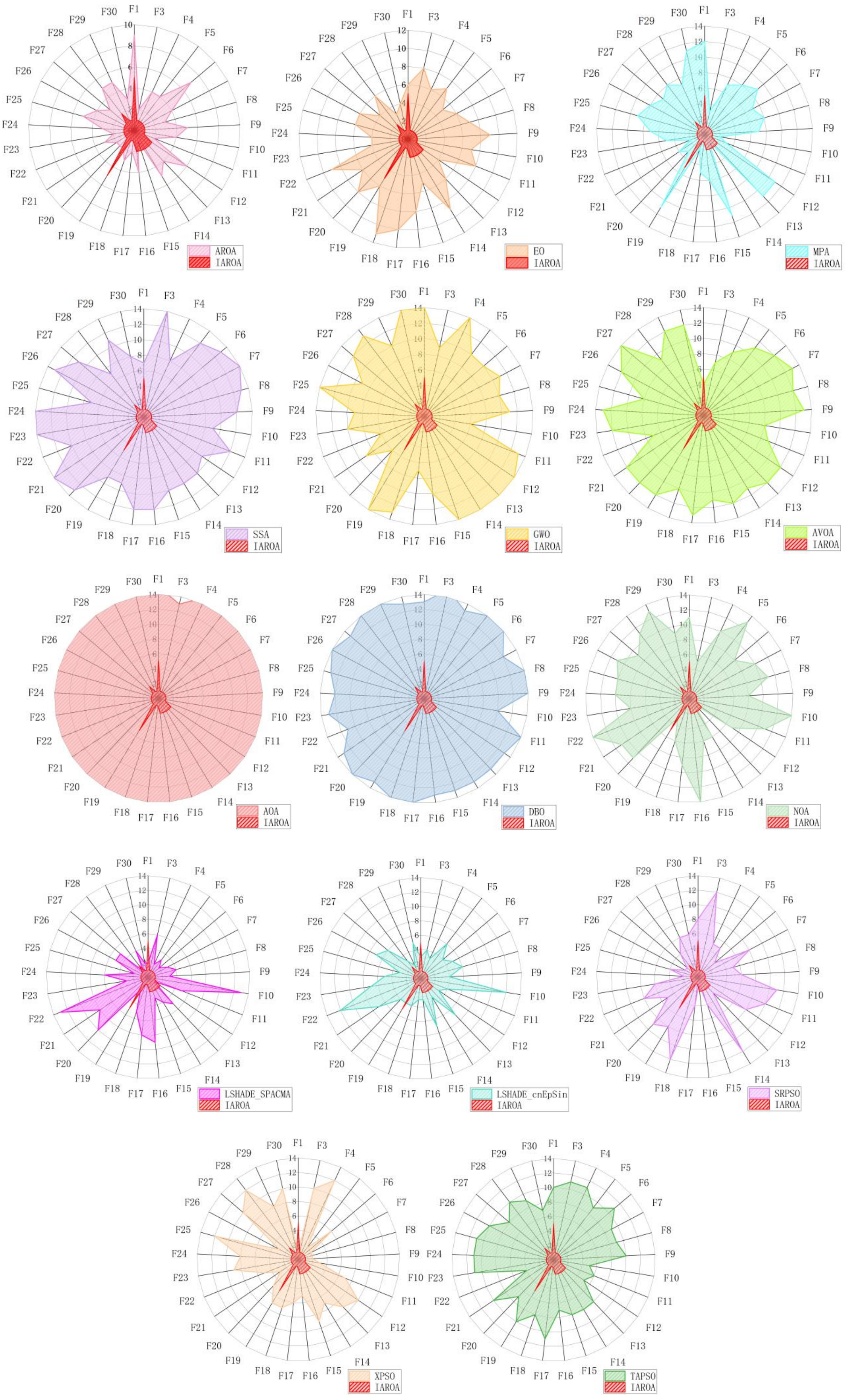
AROA
IAROA
EO
IAROA
MPA
IAROA
SSA
IAROA
GWO
IAROA
AVOA
IAROA
AOA
IAROA
DBO
IAROA
NOA
IAROA
LSHADE_SPACMA
IAROA
LSHADE_cnEpSin
IAROA
SRPSO
IAROA
XPSO
IAROA
TAPSO
IAROA

Figure S5 Radargram of the 100 dimensions CEC2017.
